# Effect of Pyridinecarboxaldehyde Functionalization on Reactivity and N-Terminal Protein Modification

**DOI:** 10.1021/jacsau.5c00238

**Published:** 2025-04-04

**Authors:** Lydia J. Barber, Ksenia S. Stankevich, Christopher D. Spicer

**Affiliations:** Department of Chemistry and York Biomedical Research Institute, University of York, Heslington, York YO10 5DD, U.K.

**Keywords:** bioconjugation, protein, aldehyde, imine, reversible reaction, aqueous chemistry

## Abstract

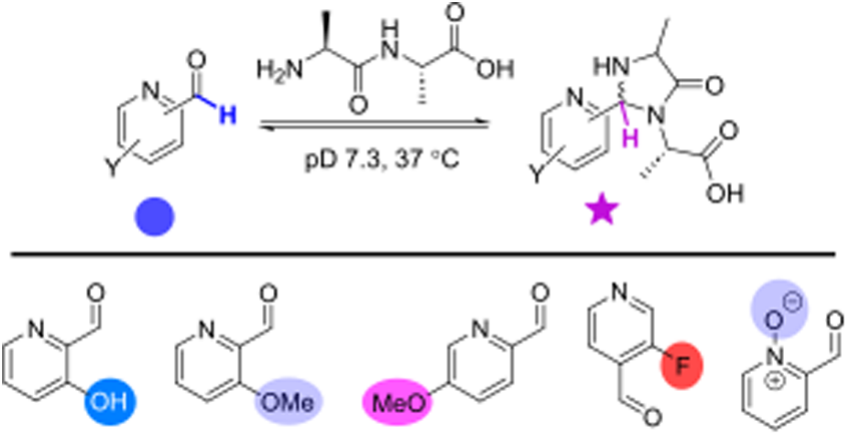

The site-selective modification of protein N-termini represents a powerful strategy for producing homogeneous bioconjugates. 2-Pyridinecarboxaldehydes have emerged as a leading reagent class in this area. However, these conjugations suffer from relatively slow rates and a degree of reversibility. In this work, we therefore studied the effects of pyridinecarboxaldehyde functionalization on N-terminal modification. This allowed us to provide insight into the factors governing relative contributions from competing reaction pathways and design criteria for second generation reagents for protein labeling. Importantly, 3-methoxy-2-pyridinecarboxaldehydes were identified as providing both accelerated and more stable protein labeling, enabling further applications of this powerful technology.

## Introduction

The N-termini of proteins offer unique handles for site-selective protein modification. In eukaryotes a large proportion of the proteome is post-translationally modified at the N-terminus, but in most bacterial and secreted proteins, including antibodies, the α-amine is chemically and sterically accessible. In recent years this has led to a surge in interest in technologies targeting this α-amine for modification. Applications ranging from N-terminal proteomics,^1,2^ to the development of protein-based therapeutics^3,4^ have been reported.

**Scheme 1:**
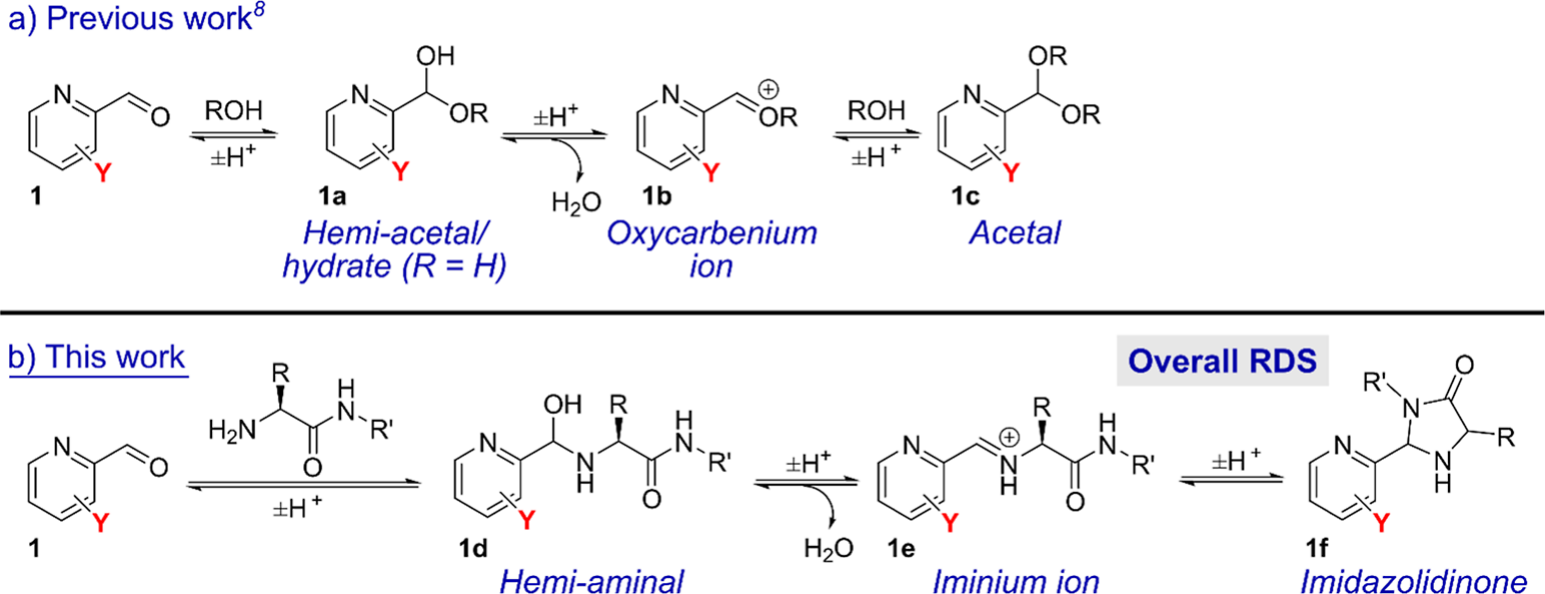
Reactions Studied in This Work, Governing Modification of Protein N-Termini with Pyridinecarboxaldehydes (PCAs) to form Imidazolidinone Conjugates

Foremost among these technologies has been the development of 2-pyridinecarboxaldehydes, **1**, as first reported by MacDonald et al. in 2015.^5^ 2-PCAs first form intermediate imines with N-terminal α-amines, which then subsequently cyclize with the adjacent primary amide of the protein backbone to form imidazolidinones (Scheme 1b). These reactions are complicated by the ability of PCAs to form hydrates in the aqueous media used for protein modification. The nature of PCA functionalization can significantly alter both the rate and reversibility of each of these steps.^6^ However, in recent work we have demonstrated that even improved 2-PCA derivatives undergo significant levels of dissociative cleavage in the presence of competitive peptides. This in turn limits applications where long-term stability is required.^7^ Moreover, imidazolidinones form with relatively slow kinetics, necessitating long reaction times and high reagent loadings. This may be detrimental for some protein targets, with cyclization of a protonated iminium ion being the rate-determining step.

In this work, we therefore set out to better understand the factors governing the complex, multistep equilibria that ultimately lead to N-terminal modification, with a view to designing improved PCA reagents for protein labeling.

## Results and Discussion

### Reagent Design

**Figure 1:**
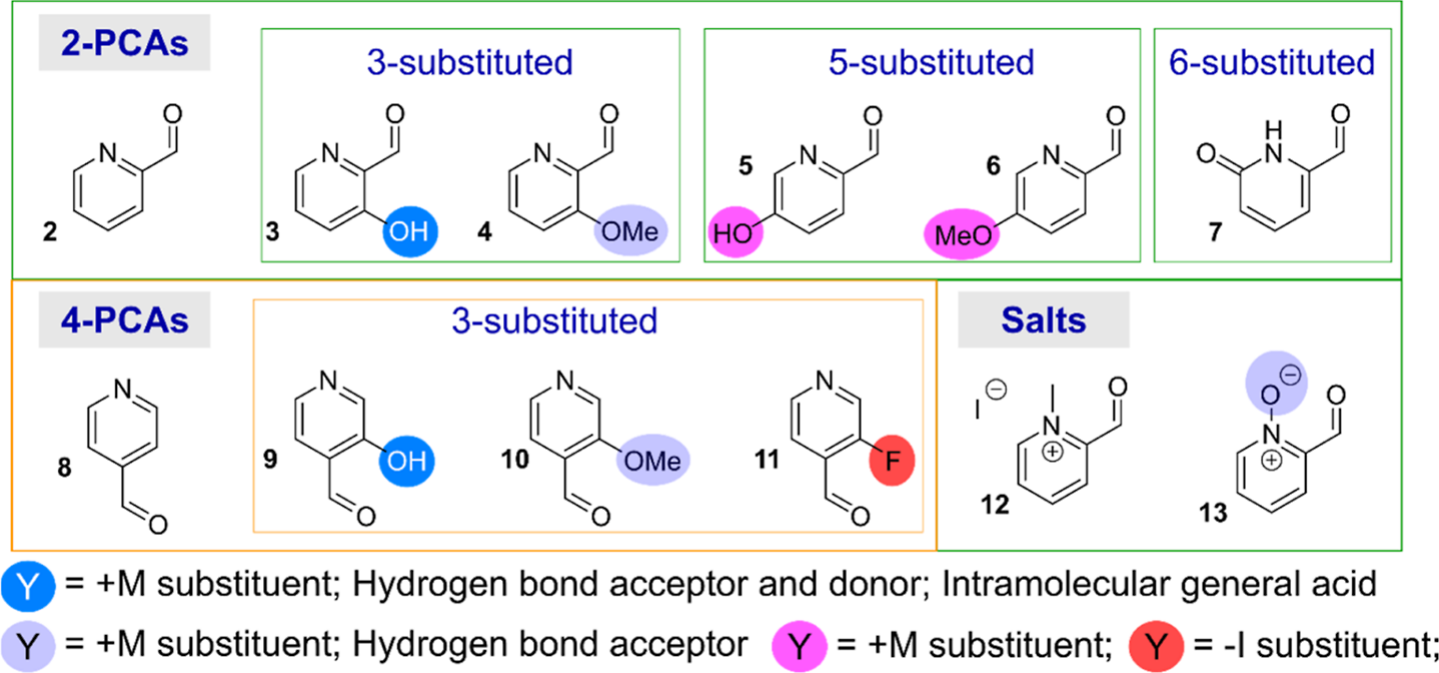
PCA substrates **2**-**13** used in this study.

We identified 12 PCA-based reagents that would provide diverse substrates for studying N-terminal modification (Figure 1). This choice was partially inspired by the work of Barman et al., who previously studied the effects of PCA substitution on hydration and acetal formation (Scheme 1a).^8^ Their work highlighted the complex interplay between various factors, including the activation of the ring by electronic contributions, intramolecular acid–base catalysis/hydrogen bonding effects, and steric factors, even within the relatively simple confines of a single reversible attack of water on the electrophilic aldehyde. We anticipated that these factors would have an amplified effect when hydrate formation was coupled to imine formation and subsequent cyclization to form an imidazolidinone (Scheme 1b). The substrates could be broadly separated into 2-PCAs (**2**-**7**), 4-PCAs (**8–11**), and 2-PCA salts (**12–13**) (Figure 1). In an aqueous environment, **7** is expected to be found predominantly as its pyridone tautomer, rather than the hydroxypyridine form, as shown.^9^

We chose to study both 2- and 4-PCAs to allow the contributions from electronic effects within the pyridine ring to be distinguished from the potential roles of the nitrogen as a general base or hydrogen-bond acceptor. Notably, in their initial report on N-terminal protein modification MacDonald et al. found 2-PCA **2** to be more efficient for the modification of angiotensin than 4-PCA **8** (84% vs 28% conversion).^5^ However, we have found the relative reactivities of different PCA derivatives to be protein dependent, and so further investigation was warranted.^7^

Hydroxyl functionalization of PCAs has been previously shown to accelerate imine formation in organic solvents,^10^ while Barman et al. showed that hydrate formation at neutral pH is reduced.^8^ Depending on the substitution pattern, we expected hydroxyl groups to have the potential to serve as (a) resonance contributors to aldehyde electrophilicity (*ortho*- and *para*-hydroxy, **3**, **5**, **9**); (b) hydrogen bond acceptors or donors (*ortho*-hydroxy only, **3** and **9**); and/or (c) general acid catalysts. In contrast, corresponding methoxy derivatives (**4** and **10**) would serve only as hydrogen bond acceptors, while also making a weaker resonance contribution to the pyridine ring and enhancing steric congestion. Similarly, we expected fluoro substitution (**11**) to inductively activate the aldehyde to nucleophilic attack, though the magnitude of this effect might be reduced by the resonance donating capabilities of the fluorine lone pair. Methylated pyridinium **12** would be expected to have similar properties, while negating the basicity and H-bonding properties of the nitrogen. Pyridine N-oxide **13** would be similarly electrophilic but would retain hydrogen bond acceptor potential. As a distinct analogue, the pyridone nitrogen of **7** would reverse the hydrogen bonding capabilities of the nitrogen, from acceptor to donor, while also negating any role played by basicity in dictating reaction outcome. However, the possibility of reactions shifting the pyridine–pyridone equilibrium in favor of the pyridine tautomer could not be discounted.

### Hydrate Formation

**Figure 2:**
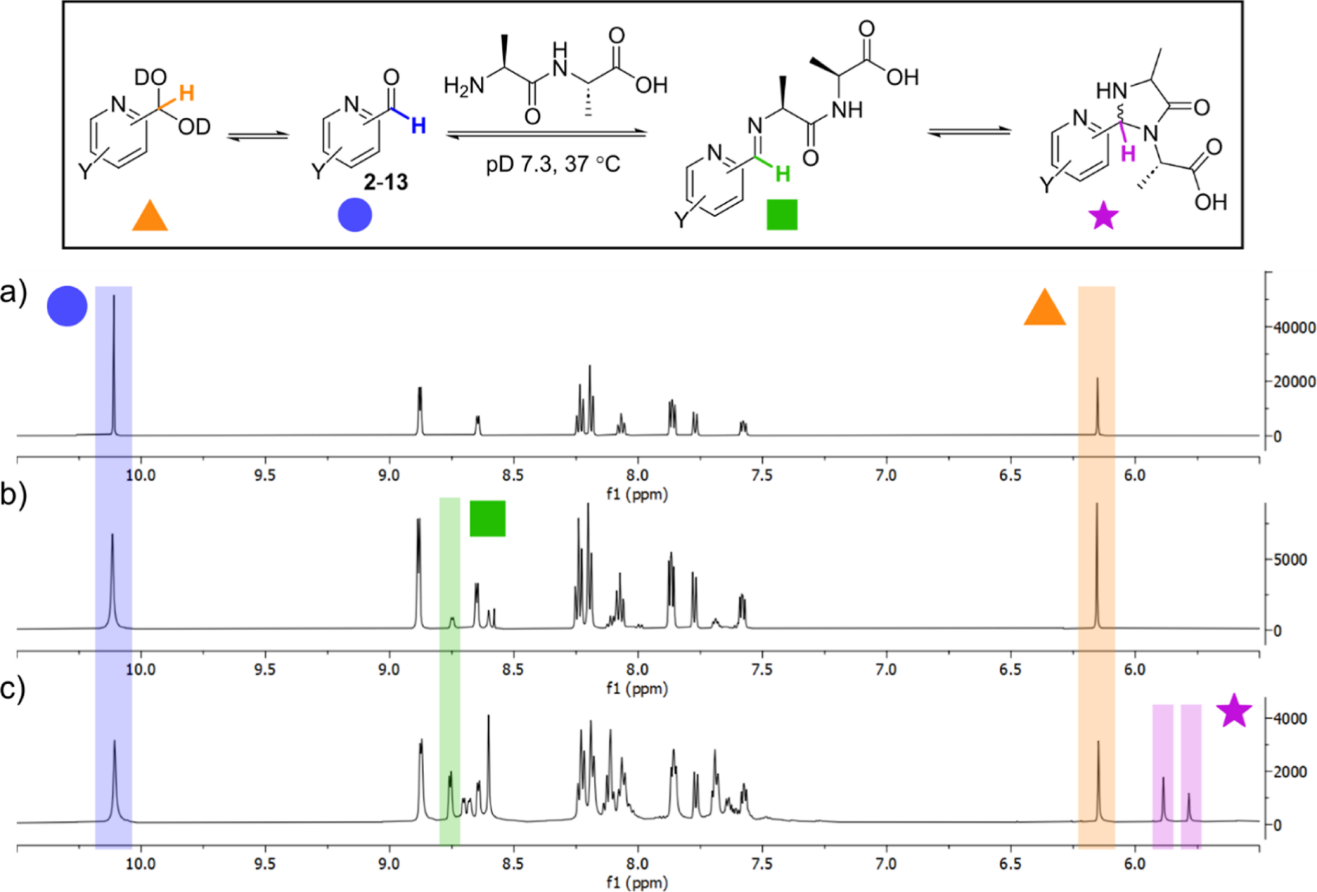
Representative ^1^H NMR spectra for (a) hydrate; (b) imine; and (c) imidazolidinone formation with 2-PCA **3**, with the diagnostic C–H peak of all 4 species highlighted.

We first set out to study the degree of hydration for each reagent under conditions relevant to N-terminal protein modification (**1** → **1a**). Though these reactions are tolerant of a range of conditions, most commonly they are performed at near neutral pH at 37 °C in a phosphate or similar buffer. Under these conditions, the pyridine will be deprotonated (pyridinium p*K*_a_ ∼ 3–5)^11^ while the hydroxyls may be partially deprotonated, as discussed further below. It is important to note that this buffering distinguishes these experiments from those previously performed by Barman, whereby hydrate formation was studied in pure water at 25 °C.^8^ Each reagent was incubated at a concentration of 50 mM in a deuterated sodium phosphate buffer (pD 7.3) at 37 °C. This allowed the equilibration of hydrate formation, which proceeds on a fast time-scale relative to N-terminal imidazolidinone formation.^7,8^ Integrations of the key aldehyde and hydrate –CH peaks in the ^1^H NMR spectra were used to calculate the ratio of the two species (Figure 2a, see Supporting Information for full analysis of all substrates).

**Figure 3:**
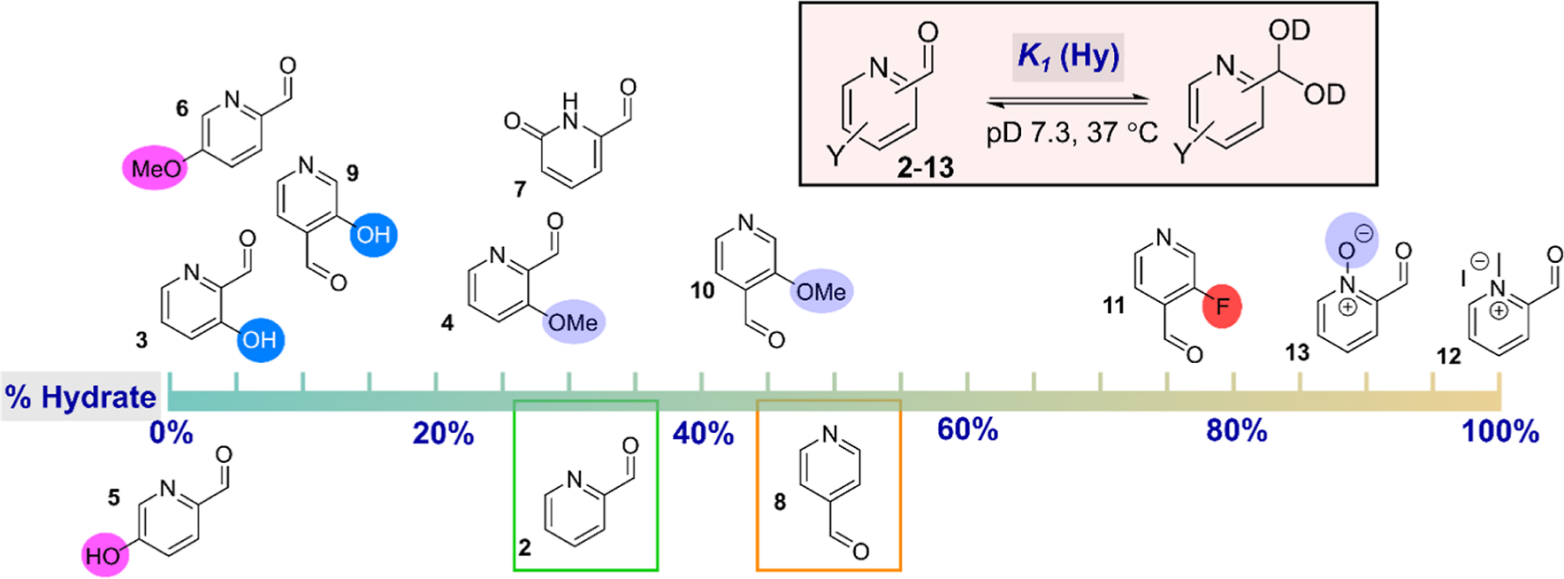
Percentage hydration of PCAs **2**-**13** at 37 °C in pD 7.3 phosphate buffer.

The degree of hydration ranged from 1% to >99% depending on the substrate (Figure 3). The distribution of reagents was largely consistent with the predicted electrophilicity of the aldehyde, with fluorinated (**11**), N-oxide (**13**), and *N*-methylated (**12**) PCAs exhibiting increased hydrate formation relative to the parent compounds **2** and **8**. For fluoro-PCA **11**, this demonstrated that inductive activation of the aldehyde dominated over any contribution from resonance stabilization. In contrast, electron-donating substituents decreased hydrate formation in all cases. This effect was far more pronounced for *ortho*-hydroxy PCAs (**3** and **9**, 28–37% decrease) than the *ortho*-methoxy analogues (**4** and **10**, 4–7% decrease). This result is consistent with the observations of Barman et al., where differences were found to be more significant than would be predicted based solely on differences between Hammet sigma values of a hydroxy or methoxy substituent. They postulated that contributions from hydroxyl anions resulting from partial deprotonation may have amplified deactivation of the carbonyl. We therefore calculated p*K*_a_ values for the hydroxyl groups of **3** (6.5), **5** (6.1), and **9** (6.9), which indicate that these compounds will be significantly deprotonated under the experimental conditions. The effects of the anion therefore dominate as predicted. Indeed, the degree of hydrate formation between the three analogues was consistent with the small differences in the hydroxyl p*K*_a_s. However, the aldehyde of *para*-methoxy analogue **6** was similarly deactivated, potentially indicating steric factors may also be contributing to the surprisingly small decrease in hydrate formation seen for the *ortho*-methoxy derivatives.

When comparing 2- and 4-substituted PCAs, 4-substituted analogues exhibited higher levels of hydrate formation in all cases (7–19% increase), again consistent with the observations of Barman et al.^8^ Interestingly, pyridone **7** also exhibited comparable behavior to 2-PCA **2**.

### Imine Formation

We next looked to investigate the formation of the key imine intermediate on the pathway to N-terminal labeling. Formation of this imine goes through the hemiaminal intermediate **1d**, which subsequently undergoes dehydration to form iminium ion **1e**. Hemiaminal formation is competitive with hydrate formation, and expected to be promoted by electron-withdrawing substituents in the same manner. Similarly, electron-donating substituents would be expected to better stabilize the iminium species. The p*K*_a_ of this iminium is strongly influenced by the substitution of the pyridine. Crugeiras et al. previously reported that an iminium formed from 4-PCA **8** had a p*K*_a_ ∼ 5, while the *ortho*-hydroxy analogue **9** had a p*K*_a_ ∼ 9, and so at neutral pH the relative protonation degree of the iminium, and thus propensity to undergo either hydrolysis or imidazolidinone formation, is likely to differ greatly.^12^ The authors demonstrated that the significant increase in iminium p*K*_a_ for **9** was due to a combination of resonance activation of the imine by the *ortho*-hydroxyl substituent, and intramolecular hydrogen bonding, in an analogous fashion to the contribution of hydrogen bonding to the stabilization of the oxocarbenium intermediate necessary for acetal formation reported by Barman et al.^8^

The α-amine of protein N-termini is less basic than the ε-amine of lysine side chains (ammonium p*K*_a_ of 6–8, vs 10 for ε-amines) due to the electron-withdrawing effects of the proximal amide. This thus affects protonation state and in turn activity at neutral pH. To best simulate this p*K*_a_, while also preventing imidazolidinone formation, we chose to use alanine dimethyl amide **14** as a reaction partner. While the use of a tertiary, rather than secondary, amide will influence both imine formation and protonation, we expected the impact to be small when compared to the use of the corresponding carboxylic acid as a model substrate.

PCAs **2**-**13** were mixed at a 1:1 ratio with alanine dimethyl amide **14** at a concentration of 25 mM in deuterated sodium phosphate buffer (pD 7.3) and incubated at 37 °C to allow equilibration. Integrations of characteristic aldehyde, hydrate, and imine –CH peaks in the ^1^H spectra were again used to calculate the ratios of the three species (Figure 2b, see Supporting Information for further details). Notably, we did not observe any signals originating from a hemiaminal species in any experiment.

**Figure 4:**
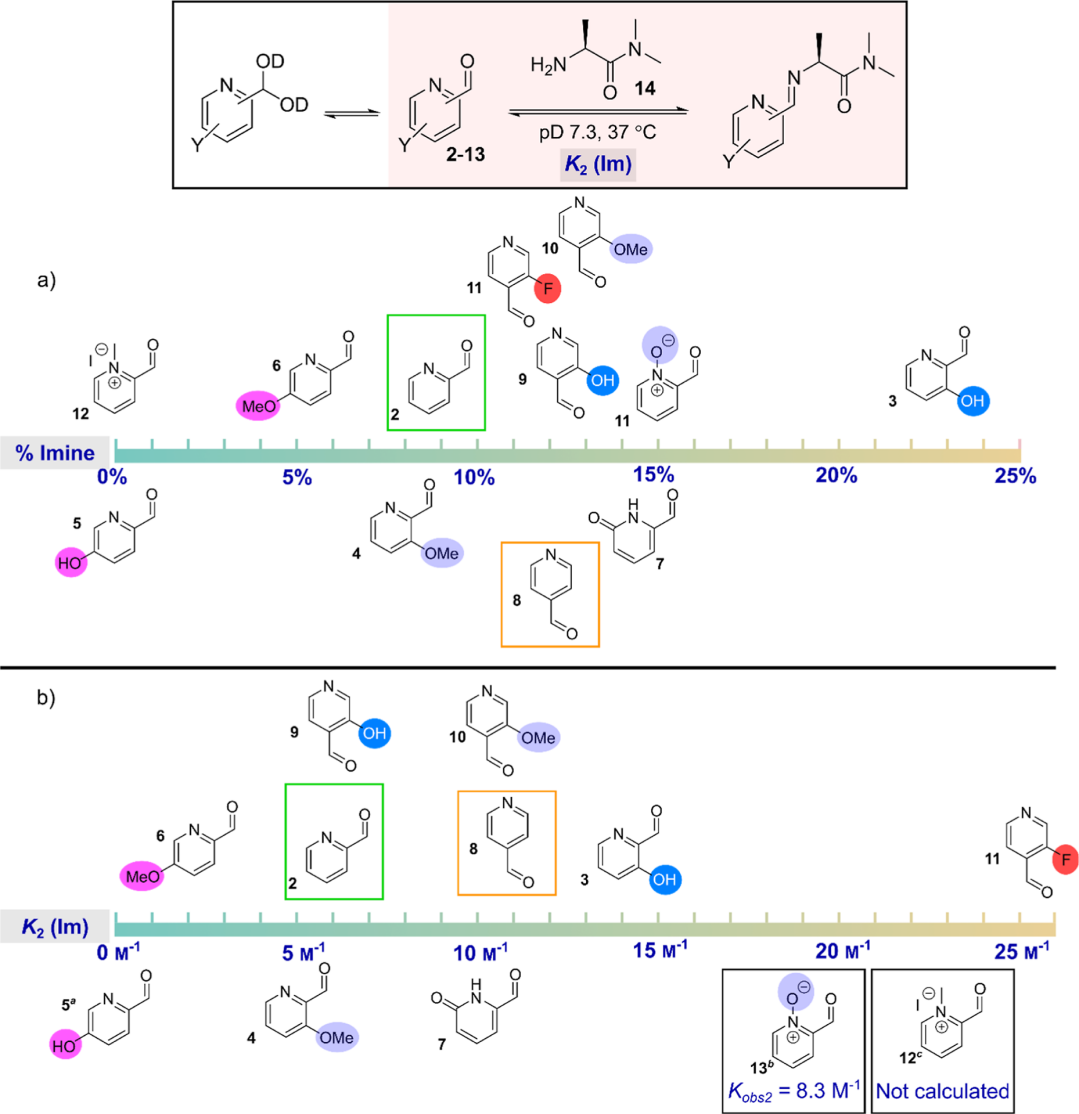
Reaction of PCAs **2**-**13** (25 mM) with alanine dimethylamide **14** (25 mM) at 37 °C in pD 7.3 buffer. (a) Percentage conversion of the PCA to the corresponding imine; (b) calculated *K*_2_ (Im) values for the equilibrium between aldehyde and imine; ^*a*^No imine was observed for **5** so *K*_2_ (Im) could not be calculated but approaches 0 m^–1^; ^*b*^No aldehyde was observed for **13** and so *K*_obs2_ for hydrate–imine equilibrium is given; ^*c*^No aldehyde or imine was observed for **12**, and so *K*_2_ could not be calculated.

From the data obtained, the equilibrium constant, *K*_2_, for imine formation could be calculated (Figure 4b, see Supporting Information for further details). Considering first the electron-deficient derivatives, *ortho*-fluoro PCA **11** displayed the highest *K*_2_ of all derivatives tested, driven by favorable amine addition. For pyridinium oxide **13**, high levels of imine formation were also observed, to the extent that no aldehyde was observed in the experiment. As a result, *K*_2_ could not be calculated, and the observed equilibrium constant, *K*_obs2_, between hydrate and imine was instead calculated (but cannot be directly compared to *K*_2_ calculated for the other derivatives). In contrast, no imine was observed for methylated pyridinium **12**, in this case because of the high levels of competitive hydration which depleted aldehyde availability for imine formation.

Within the electron-rich derivatives, *para*-hydroxy PCA **5** was notable in failing to generate detectable imine, dictated by the reduced electrophilicity of the aldehyde and less favorable hemiaminal formation. In contrast, *ortho*-hydroxy 2-PCA **3** had the highest levels of imine formation (22%) among all the derivatives tested. This contrasting behavior between the *ortho*- and *para*-hydroxy isomers indicates the hydroxy group of **3** is able to act as a hydrogen bond acceptor and stabilize the iminium ion formed. For the *ortho*-methoxy derivatives **4** and **10**, similar *K*_2_ values were obtained to the parent PCAs. These results are analogous to the results obtained by Barman et al. for acetal formation, and indicate a delicate balance between electronic contributions slowing amine attack, but subsequent dehydration being favored by the hydrogen bonding capacity of these groups, ultimately dictating the overall equilibrium.^8^ However, in contrast to the results found for acetal formation, we surprisingly found that *ortho*-hydroxy 4-PCA **9** exhibited a lower *K*_2_ than the parent 4-PCA **8**.

### Imidazolidinone Formation

Having studied hydrate and imine formation, and determined equilibrium constants for each step, we were now in a position to analyze the factors governing imidazolidinone formation. In an analogous experiment to those described above, each PCA derivative was incubated with 1 equiv of the model dipeptide Ala–Ala at 37 °C. In our previous study, imidazolidinone formation from 2-PCAs was found to be relatively slow, and ^1^H NMR spectra were therefore recorded at regular intervals over a 16 h time period.^7^ MacDonald et al. previously reported the presence of two characteristic singlets at ∼6 ppm originating from the two imidazolidinone diastereomers as being characteristic of cyclization. This region is free from other peaks in our experiments other than the hydrate –CH.^5^ These peaks could therefore be used as diagnostic of imidazolidinone formation and integrated relative to the aldehyde, imine, and hydrate –CH peaks to give a complete picture of the reaction (Figure 2c, see Supporting Information for full details). The data obtained allowed us to analyze imidazolidinone formation at two levels, each providing complementary insights into the reaction.

**Figure 5:**
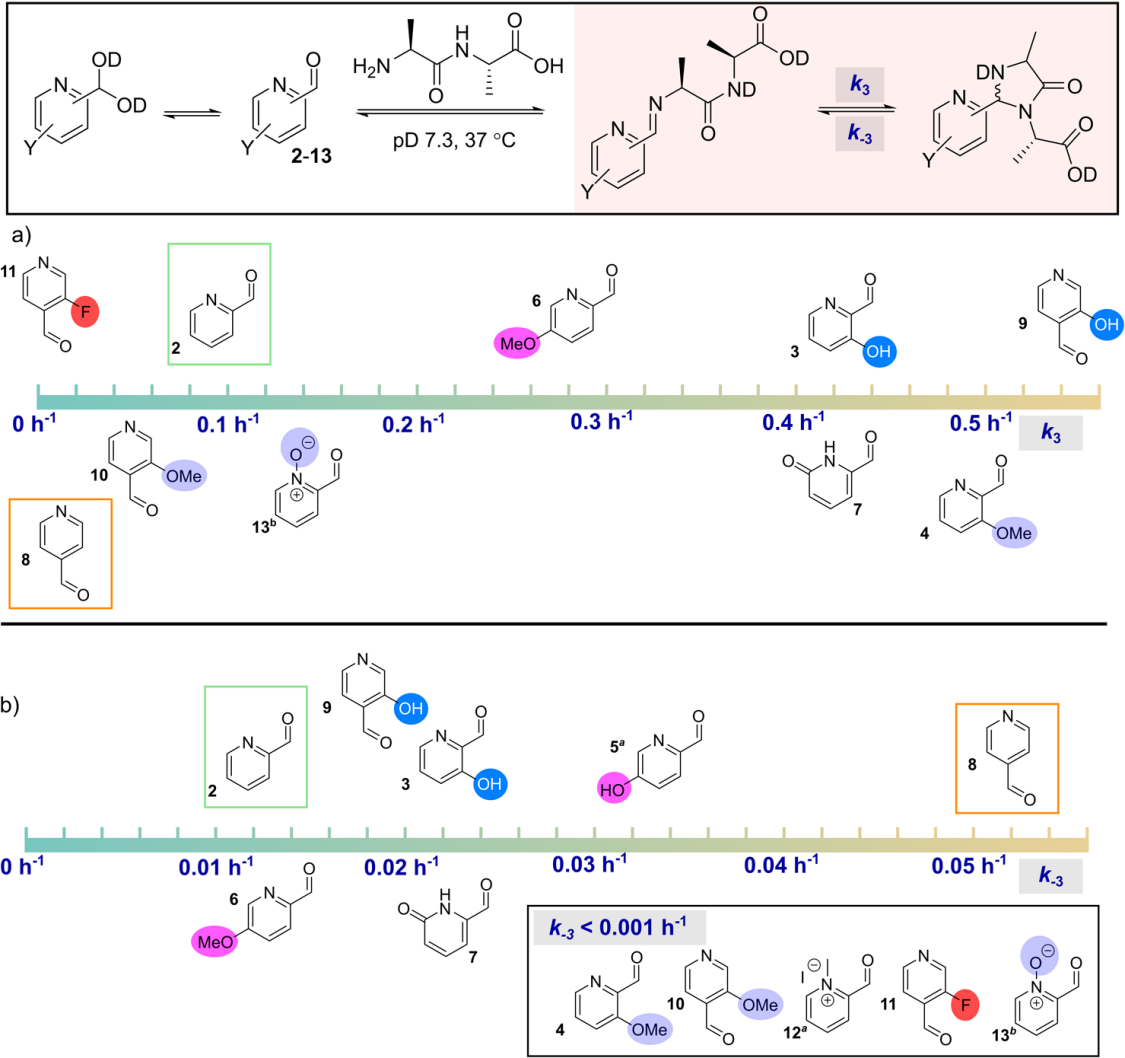
Reaction of PCAs **2**-**13** (50 mM) with dialanine (50 mM) at 37 °C in pD 7.3 buffer. (a) Calculated forward rate constants, *k*_3_, for the cyclization of the imine into an imidazolidinone. Nb. For PCAs **5** and **12** no imine was shown in previous experiments and so no *k*_3_ could be calculated. Steady-state approximations could be applied to give the second-order observed rate constants *k*_obs4_ = 0.44 M^–1^ h^–1^ and *k*_obs5_ = 0.18 M^–1^ h^–1^ respectively, as described in the Supporting Information; (b) calculated reverse rate constants, *k*_–3_, for the ring-opening of imidazolidinones to the corresponding imine; ^*a*^For **5** and **12** reverse reaction rates, *k*_obs-4_ and *k*_obs-5_ respectively, were based on calculations encompassing the steady state approximation detailed above and in the Supporting Information; ^*b*^For **13** values of both *k*_3_ and *k*_–3_ were derived based on *K*_obs2_ as described in Figure 2 and the Supporting Information.

A first analysis considered solely the cyclization of the imine to form the imidazolidinone product. Since cyclization is rate-limiting and several orders of magnitude slower than imine and hydrate formation, *K*_1_ and *K*_2_ could be used to build and fit a kinetic model to the NMR data. This allowed us to obtain both forward and reverse first-order rate constants for cyclization, *k*_3_ and *k*_–3_ respectively, as well as the equilibrium constant for cyclization, *K*_3_, where relevant (Figure 5, see Supporting Information for details). For PCAs **5** and **12**, imidazolidinone formation was observed despite the lack of imine formation seen in the previous experiment. In this scenario, a steady-state approximation was applied to calculate the observed rate constants, *k*_obs4_ and *k*_obs5_ (second-order), and *k*_obs-4_ and *k*_obs-5_ (first-order), for reversible formation of the imidazolidinone products from the aldehydes or hydrates, respectively. However, these constants could not be directly compared to *k*_3_ and *k*_–3_, and so interpretation of the reactivity of these analogues could not be undertaken during this first analysis.

On the other hand, we could consider the overall picture of total imidazolidinone formation as a function of time, encompassing the overall complex balance between hydrate, imine, and imidazolidinone formation (see Supporting Information Section 2). This analysis allowed a more qualitative comparison of the data to be performed, including integrating the behavior of **5** and **12**.

In all cases, electron-donating substituents accelerated imidazolidinone formation. Since electron-donation is expected to decrease imine electrophilicity, this effect is most likely due to the increased p*K*_a_ of the iminium. Higher degrees of protonation relative to the parent PCA, would in turn favor nucleophilic attack, as described above.^12^ This effect would be expected to be highest for *ortho*-substituted analogues where hydrogen bonding would serve to increase the p*K*_a_ further, and the lower *k*_3_ of *para*-methoxy PCA **6** would appear to support this. Though a comparable *k*_3_ value for **5** could not be obtained, overall conversion to the imidazolidinone product was found to be slow (∼20% after 16 h), as it was for **6**, lending further support to this hypothesis. An exception to this general behavior was *ortho*-methoxy 4-PCA **10** which exhibited a surprisingly low *k*_3_.

Considering the reverse reaction, the favorable acceleration of ring closing induced by electron-donating substituents was partially tempered by an analogous increase in ring opening for hydroxy-substituted PCAs. Though *K*_3_ was higher overall than for the parent PCAs, and total imidazolidinone formation was therefore both accelerated and increased, the net result of these factors was that incomplete cyclization was achieved at equilibrium. In contrast, for the *ortho*-methoxy analogues **4** and **10**, the data fit an irreversible kinetic model indicating that *k*_–3_ was below the threshold of detection within these experiments (<1 × 10^–3^ h^–1^). The net effect of this on imidazolidinone formation was most significant for **4**, with equilibrium having not been reached by the end-point of the experiment. When combined with the observed high *k*_3_ these data highlighted reagent **4** in particular as a highly promising reagent for N-terminal modification, if these observations could be translated to the more complex setting of protein scaffolds.

A similar decrease in *k*_–3_ was observed for the electron-deficient derivatives **11**-**13** for which *k*_–3_ could not be measured (*k*_obs5_ in the case of **12**). Fluoro-PCA **11** exhibited a very slow rate of cyclization (notably an order of magnitude lower than any other derivative tested, ∼10^–3^ h^–1^ vs ∼10^–2^ h^–1^ for the next slowest derivative), and overall accumulation of imidazolidinone was slow for both **11** and methylpyridinium **12**. This is indicative of the lower degree of imine protonation reducing susceptibility to ring-closing. However, interestingly pyridine N-oxide **13** exhibited a small increase in *k*_3_ over the parent PCA **2**, while also fitting to an irreversible kinetic model.

### Protein Modification

Each of the PCA derivatives **2**-**13** was screened in the modification of four proteins: RNase A (N-terminal Lys) and myoglobin (N-terminal Gly), as model proteins that we previously showed to give variable levels of labeling depending on the reagent used,^7^*E. coli* thioredoxin (N-terminal Ser), and the antiprostate specific membrane antigen nanobody JVZ-007 (N-terminal Ser).^13^ The nature of the N-terminal residue significantly influences both labeling kinetics and stability. For example, peptides bearing N-terminal glycines were previously found to exhibit greatly reduced reactivity, as well as undergoing a small degree of dimodification.^5^ In our previous work, this effect manifested as a low degree of modification for the N-terminal glycine of myoglobin,^7^ however we considered that other PCA derivatives may be able to alter this performance. Moreover, local protein environment will also strongly influence labeling, due to differences in steric congestion, α-amine p*K*_a_, and contributions from neighboring side chains. Therefore, a greater degree of variability was expected in terms of reagent performance, relative to our tightly controlled small molecule studies.

**Figure 6:**
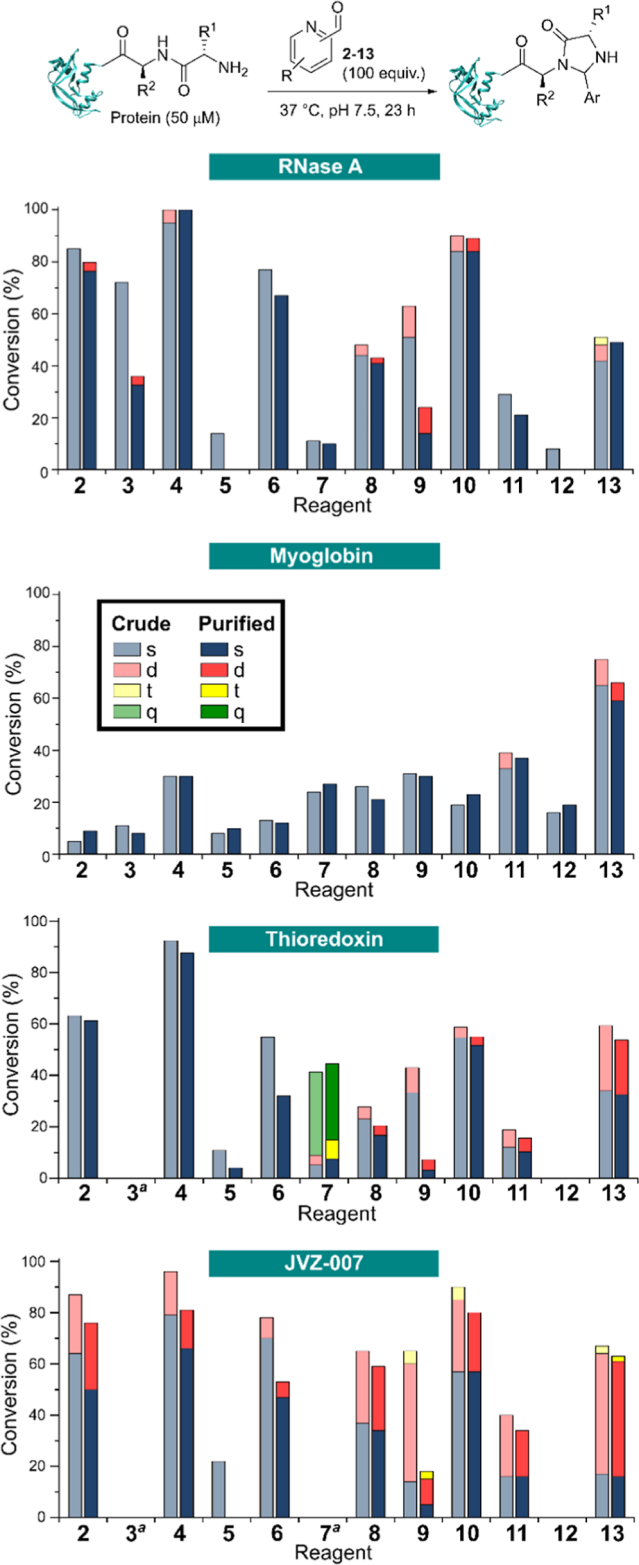
Conversions for the modification of RNase A, myoglobin, thioredoxin, and JVZ-007 with PCAs **2**-**13** before (crude) and after (purified) overnight dialysis at 4 °C; s = single, d = double, t = triple modification, q = quadruple modification. ^a^No protein was detected by LC–MS.

Conversions were determined on both crude reaction mixtures and after dialysis overnight to remove excess reagent. If imidazolidinone formation was unstable, this dialysis would be expected to lead to a reduction in conversion, as well as the hydrolysis of any transient imines formed at lysine residues (Figure 6).

For RNase A, thioredoxin, and JVZ-007, *ortho*-methoxy-PCA **4** was found to give the most effective protein labeling, in line with the small molecule studies. Focusing on RNase A first, **4** had the highest single labeling efficiency, with labeling found to be stable after overnight dialysis as would be expected based on the previous discussion. In contrast, hydroxy-PCAs **3** and **9** exhibited moderate labeling efficiencies after 24 h, but were found to be highly unstable after overnight incubation (39 and 37% reduction in labeling, respectively). Similarly, *para*-hydroxy-PCA **5** gave low and unstable modification, in line with the high *k*_–3_ calculated in the small molecule studies.

Analogous behavior of the electron-rich methoxy- and hydroxy-substituted PCAs was observed for thioredoxin and JVZ-007. In both cases reagent **4** gave the highest yield of labeled protein, with consistent levels of singly labeled species both before and after analysis further highlighting the superior stability of labeling. These effects can be partially attributed to the hydrogen-bonding capacity of the *ortho*-methoxy group, as observed in the small molecule labeling studies, with *para*-methoxy derivative **6** displaying inferior labeling efficiency and undergoing cleavage upon dialysis with both proteins (23 and 28% reduction in labeling with thioredoxin and JVZ-007, respectively).

Further evidence supporting the beneficial effects of *ortho*-methoxylation were found through the use of methoxy-4-PCA **10**, which provided an improvement in labeling efficiency over the parent 4-PCA **8** for all three proteins, in line with the small molecule studies. However, for both **8** and **10** significantly higher protein labeling efficiencies were observed than would have been predicted from our small molecule studies. For **8** in particular, conversions were higher than would have been predicted given the low value of *k*_3_ and high value of *k*_–3_ obtained from our studies of imidazolidinone formation (with a calculated equilibrium constant *K*_3_ = 0.5). Given that **8** and **10** share a common 4-PCA core it is likely that these discrepancies have a common origin. Although small molecule studies are a vital tool in bioconjugation research that can provide important insights such as those obtained for the behavior of the other derivatives tested, this result emphasizes that they do not always correlate fully with protein data, and there can be complicating effects that go beyond protein-to-protein variability. However, despite this discrepancy, the expected improvement in labeling from methoxy-substituted **10** over parent PCA-**8** is consistent with our wider observations and analysis, and still provides important insight into reagent design. Notably, this improvement only served to provide comparable labeling behavior to unfunctionalized 2-PCA **2**. This validates the previous observations of MacDonald et al. that 2-PCAs generally provide superior protein labeling than their 4-substituted analogues.^5^

Overall, these results highlight *ortho*-methoxy-2-PCAs as highly promising scaffolds for producing the next generation of N-terminal targeting reagents, with greatly accelerated and stabilized imidazolidinone formation. In contrast, for both thioredoxin and JVZ-007, labeling with *ortho*-hydroxy-PCA **9** was again highly unstable, while **5** gave low and unstable labeling. This is in line with the high rate of observed imidazolidinone ring-opening, *k*_–3_, found in our small molecule studies for all hydroxylated-PCAs, which negates the benefits of high *k*_3_ under the dilute conditions used for protein labeling. Collectively, these results demonstrate that hydroxy-PCAs are unsuitable reagents for stable protein labeling. Notably, when *ortho*-hydroxy-PCA **3** was used for labeling, no protein signal could be detected by LC–MS for both thioredoxin and JVZ-007, with pyridone **7** having a similar effect on JVZ-007 and leading to greatly reduced MS signal intensity for both thioredoxin and RNase A. On thioredoxin, **7** also led to high levels of off-target modification, with up to quadruple modification being observed both before and after purification.

Within the electron-deficient PCAs studied, observations were largely in line with the small molecule studies described above. Methylated pyridinium **12** had been observed to be predominantly hydrated and imine formation had not been observed in the small molecule studies. This would in turn limit availability of the key imine for imidazolidinone formation, despite a small quantity of this product being formed in reactions with DiAla (see Supporting Information Table S3). As a result, only very minimal levels of protein labeling were observed. Labeling with fluoro-PCA **11** was slightly improved, but still poor. This is in line with the very low *k*_3_ value observed for imidazolidinone formation, which was at least an order of magnitude lower than that obtained for all other derivatives (∼10^–3^ h^–1^; nb. A comparison cannot be made to **12**, since *k*_3_ could not be calculated). In contrast, pyridine N-oxide **13** proved to be a more effective probe, in line with its far greater *k*_3_ in comparison to fluoro-PCA **11** (∼10^–1^ vs 10^–3^ h^–1^).

In line with our previous observation of the N-terminus of myoglobin being less reactive than other proteins, reagents **2**-**10** all gave low conversions, albeit with methoxy-PCA **4** being the most effective again. However, surprisingly the electron-deficient PCAs proved more effective, with pyridine N-oxide **13** in particular providing 66% conversion to a singly labeled species after purification. Similarly, fluoro-PCA **11** gave 37% labeling, which while moderate still made it the second most effective of all of the reagents screened. This observation emphasizes the variability between different substrates, and while general trends such as our finding that *ortho*-methoxy-2-PCAs provide an advantageous scaffold for reagent design, the screening of a library of reagents against a particular protein of interest is still warranted at an early stage. In this particular scenario, there are a number of possible explanations for this altered preference for electron-deficient PCAs over the electron-rich analogues that provided highest labeling efficiency on the other three model proteins. One possibility is that the presence of an N-terminal glycine, which was shown by MacDonald to be less reactive than other amino acids within a peptide screen against 2-PCA **2**, may alter reactivity. This could result from increased conformational freedom or increased nucleophilicity of the α-amine.^5^ Alternatively, the altered reactivity may simply be a result of the local environment at the N-terminus of myoglobin, for example through promoting imine protonation and thus ring-closing to form the imidazolidinone product that was disfavored in our small molecule studies. However, further studies studying multiple N-terminal variants of the same protein or comparisons of proteins sharing similar overall structures but varied N-terminal domain properties are necessary to elucidate the true origins of such variability.

## Conclusions

In this paper we have studied the effects of PCA functionalization on the key reaction steps governing the modification of protein N-termini. Competing aldehyde hydration, iminium formation, and finally cyclization of this intermediate iminium to form the key imidazolidinone species all contribute to N-terminal labeling. Our results indicate a complex and delicate balance exists between factors governing each individual step, and that a consideration of each is important to understand the relative performance of different reagent classes in N-terminal functionalization.

In particular, we identify 3-methoxy-2-PCAs, such as **4**, as leading candidates for next-generation N-terminal labeling probes. These derivatives enhance and accelerate imidazolidinone formation, while at the same time, imidazolidinone hydrolysis is also prevented, leading to more stable labeling and addressing one of the major drawbacks of previously reported reagents. This in turn will enhance the utility of N-terminal labeling technologies in the future.

Notably, while strong indicative trends exist across the derivatives tested, the variability in protein N-terminal amino acid and local environment still impact labeling efficiency and the optimal reagent for a given target. The results presented here advocate for the screening of a small library of simple PCA derivatives against a new protein target to maximize compatibility prior to the synthesis of a functional probe. Our results indicate that in a majority of cases 3-methoxy-2-PCAs will emerge as a preferred reagent scaffold in such a screen, enabling the full potential of N-terminal selective labeling to be realized.

## Methods

### Hydrate Formation

^1^H NMR spectra of PCAs **2**-**13** (50 mM in 100 mM deuterated Na phosphate buffer, pD 7.3) were recorded at 37 °C to determine the ratio between aldehyde and hydrate forms. It was assumed that equilibria were reached quickly before NMR spectra were recorded. Note: due to reduced quantities of material available, the data for 2-PCA **12** was collected at a reduced concentration of 15 mM. The concentration of aldehyde and hydrate were calculated via the relative integrals of diagnostic ^1^H NMR signals outlined in the Supporting Information.

### Imine Formation

An aliquot of each PCA/hydrate solution (300 μL, 50 mM, 15 μmol, 1 equiv, in 100 mM deuterated Na phosphate buffer, pD 7.3) was added to a solution of dimethyl amide **14** (300 μL, 50 mM, 15 μmol, 1 equiv, in 100 mM deuterated Na phosphate buffer, pD 7.3) and the reaction mixtures were incubated at 37 °C until equilibrium had been reached. The concentration of aldehyde, hydrate, and imine were calculated via the relative integrals of diagnostic ^1^H NMR signals outlined in the Supporting Information.

### Imidazolidinone Formation

A solution of DiAla (150 μL, 100 mM, 15 μmol, 1 equiv) was added to solutions of reagents **2**-**13** (150 μL, 100 mM, 15 μmol, 1 equiv), both in deuterated sodium phosphate buffer (100 mM, pD 7.3). The reactions were incubated at 37 °C for 16 h and conversion was followed by ^1^H NMR spectroscopy at 30 min intervals. At each time point, the concentration of aldehyde, hydrate, imine, and sum of the imidazolidinone diastereomers were calculated via the relative integrals of diagnostic ^1^H NMR signals outlined in the Supporting Information.

### Protein Modification

*Conditions for the modification of proteins with PCAs **2**-**13** were adapted from those reported by MacDonald et al*.^5^ A stock solution of PCA **2**-**13** (85 μL, 10 mM, 850 nmol, 100 equiv, in 50 mM pH 7.5 sodium phosphate buffer) was added to a solution of protein (85 μL, 100 μM, 8.5 nmol, 1 equiv, in 50 mM pH 7.5 sodium phosphate buffer), and the mixture incubated at 37 °C for 23 h with agitation (1000 rpm). Conversion was determined by LC–MS analysis without purification (crude). Protein conjugates were then purified by dialysis to remove excess reagent (4 °C, 3.5 kDa MWCO; 1 × 50 mM pH 7 sodium phosphate buffer, 2 h; 1 × water, 3 h; 1 × water, 16 h; 1 × water, 4 h), and conversion was again determined by LC–MS analysis (purified).

## Supporting Information Available

Contains details of all experiments, including small molecule synthesis, NMR studies, and protein labeling (PDF)
