# Supplementary material for: Effect of Pyridinecarboxaldehyde Functionalization on Reactivity and N-Terminal Protein Modification
Source: JACS Au. 2025 Apr 4;5(4):1983–91. doi: 10.1021/jacsau.5c00238 (PMC12042018; doi:10.1021/jacsau.5c00238)
Supplement: Supplementary file 1 — au5c00238_si_001.pdf [file au5c00238_si_001.pdf]

The effect of pyridinecarboxaldehyde functionalisation on reactivity and N-terminal protein modification

Lydia J. Barber,[a] Ksenia S. Stankevich,[a] and Christopher D. Spicer*[a]

[a]Department of Chemistry and York Biomedical Research Institute, University of York, Heslington, YO10 5DD, UK.

Table of contents

S2 General considerations

S3 Reagent synthesis

S6 Hydrate, imine, and imidazolidinone formation

S12 Representative ^{1}H NMR Spectra

S25 pK_a calculation

S25 Protein modification

S56 References

General considerations

Proton nuclear magnetic resonance (^{1}H NMR) spectra were recorded on a Jeol ECX-400 (400 MHz) or Bruker AVIIIHD (600 MHz) spectrometer. NMR shifts were assigned using COSY, HSQC and HMBC spectra. All chemical shifts are quoted on the δ scale in ppm using residual solvent as the internal standard (^{1}H NMR: $CDCl_3$ = 7.26; D_2O = 4.79). Coupling constants (*J*) are reported in Hz with the following splitting abbreviations: s = singlet, d = doublet, t = triplet, q = quartet, m = multiplet, br = broad. Melting points (m.p.) were recorded on a Gallenkamp melting point apparatus.

Thin layer chromatography (TLC) was carried out using aluminium backed sheets coated with 60 F_{254} silica gel (Merck). Visualization of the silica plates was achieved using a UV lamp (λ_{max} = 254 nm), or ninhydrin (1.5% ninhydrin, 3% AcOH in *n*-butanol). Flash column chromatography was carried out using Geduran Si 60 (40-63 µm) (Merck). Mobile phases are reported as % volume of more polar solvent in less polar solvent. Deionized water was used for chemical reactions and Milli-Q purified water for protein manipulations. All other solvents were used as supplied (Analytical or HPLC grade), without prior purification. Reagents were purchased from Sigma-Aldrich and used as supplied, unless otherwise indicated. Brine refers to a saturated solution of sodium chloride. Petrol refers to the fraction of petroleum ether boiling in the range 40-60 °C. Anhydrous magnesium sulfate ($MgSO_4$) was used as the drying agent after reaction workup unless otherwise stated. RNase A from bovine pancreas (powder, 50 units/mg protein), myoglobin from equine skeletal muscle (powder, 95-100%), and recombinant thioredoxin from *E. coli* were purchased from Sigma Aldrich. JVZ-007 was expressed recombinantly in *E. coli* as previously reported.[1] Protein figures were built using structural data obtained by Chatani *et al* (RNase A, PDB 1FS3),[2] Maurus *et al* (Myoglobin, PDB 1WLA),[3] or Andersen *et al* (Thioredoxin, PDB 1AIU)[4]. The structure of JVZ-007 was predicted using AlphaFold.

Liquid chromatography-mass spectrometry (LC-MS) was performed on a HCTultra ETD II ion trap spectrometer, coupled to an Ultimate300 HPLC using an Accucore C18 column (150 × 2.1 mm, 2.6 µm particle size) or Accucore C4 (100 × 4.6 mm, 5 µm particle size). Water (solvent A) and acetonitrile (solvent B), both containing 0.1% formic acid, were used as the mobile phase at a flow rate of 0.3 mL min^{-1}. LC traces were measured via UV absorption at 220, 270, and 280 nm. The gradient was programmed as shown below:

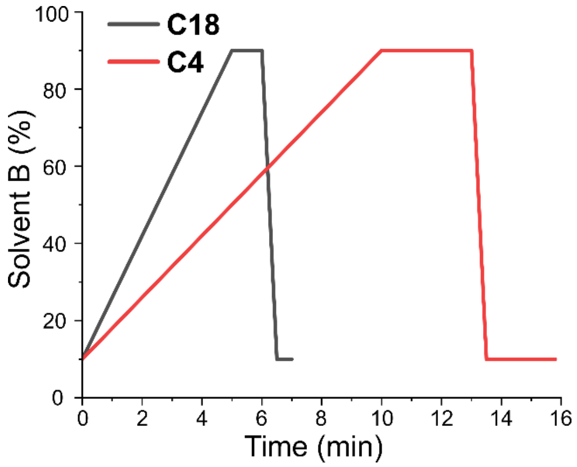

Spectra were analysed using the Bruker Data Analysis 4.4 software. Spectra were charge deconvoluted using ESI Compass 1.3 for RNase A (13000-16000 Da), myoglobin (16000-20000 Da), thioredoxin (10000-15000 Da) and JVZ-007 (8000-15000 Da). Data are presented showing the raw ion series MS data on the left, and the deconvoluted spectra on the right. Expected masses were calculated relative to reported [**SM**+H]$^+$ values of 13681 Da (RNase A)[5], 16951 Da (myoglobin)[6], 11674 (thioredoxin)[7] and 12124 (JVZ-007)[1]. **SM** = unmodified starting material, **P** = modified product, s = single modification, d = double modification, t = triple, q = quadruple modification. Nominal and exact *m/z* values are reported in Daltons.

1. Reagent synthesis

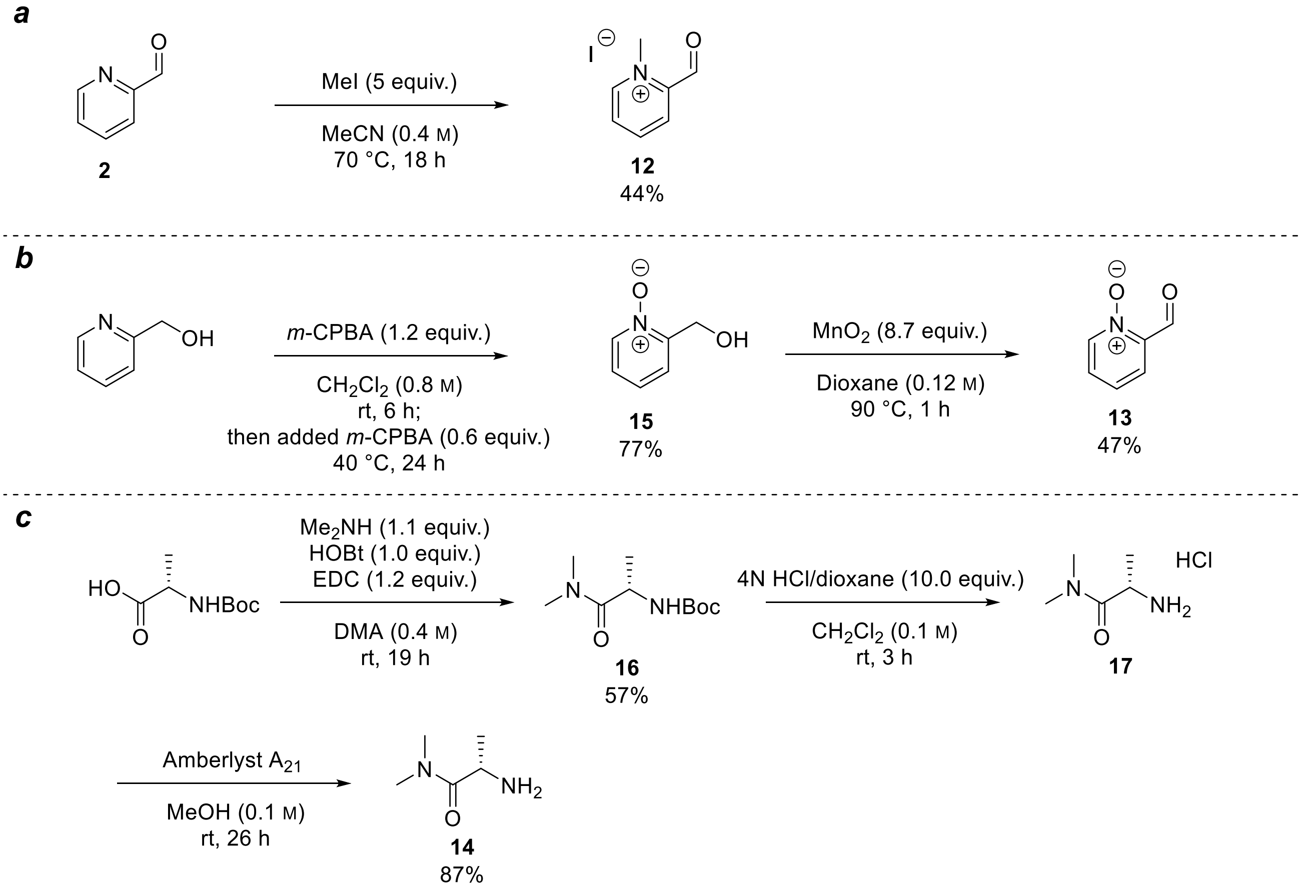

Scheme S1. Synthetic route for reagents: (*a*) 2-PCA **12**; (*b*) 2-PCA **13**; and (*c*) amino acid **14**.

*(a) 2-PCA **12***

2-formyl-1-methylpyridin-1-ium iodide (12)

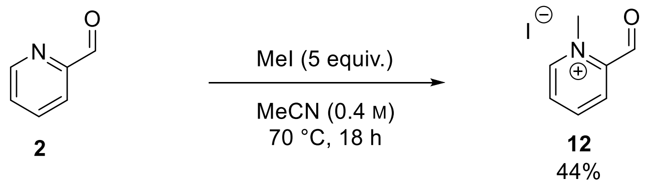

Methyl iodide (0.62 mL, 10 mmol, 5 equiv.) was added to a solution of 2-PCA **2** (0.19 mL, 2.0 mmol, 1.0 equiv.) in CH_2Cl_2 (5 mL, 0.4 M), and the reaction mixture was stirred at 70 °C for 18 h. After cooling to room temperature, the reaction mixture was filtered and the precipitate washed with diethyl ether (2 × 10 mL), to obtain the title compound as a yellow solid (220 mg, 0.88 mmol, 44%), which formed the hydrate on storage with spectroscopic data in accordance with the literature.[8] **^{1}H NMR** (400 MHz, D_2O) $\delta_{H:}$ 4.42 (3H, s, Me), 6.42 (1H, s, ArCH(OH)$_2$), 8.00 (1H, ddd, *J* = 7.8, 6.1, 1.6 Hz, ArH5), 8.35 (1H, dd, *J* = 8.1, 1.6 Hz, ArH3), 8.59 (1H, ddd, *J* = 8.1, 7.8, 1.4 Hz, ArH4), 8.80 (1H, dd, *J* = 6.2, 1.4 Hz, ArH6).

(b) *2-PCA* ***13***

2-(Hydroxymethyl)pyridine-1-oxide (15)

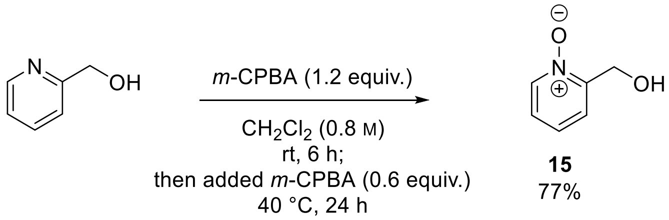

15
77%

m-CPBA (0.96 g, 5.5 mmol, 1.2 equiv.) was added to a solution of 2-(hydroxymethyl)pyridine (0.44 mL, 4.6 mmol, 1.0 equiv.) in CH_2Cl_2 (6 mL, 0.8 M) and the reaction mixture was stirred at rt for 6 h. At this point, TLC analysis indicated that the reaction was incomplete, so a further portion of *m*-CPBA (0.47 g, 2.7 mmol, 0.59 equiv.) was added and the reaction mixture was stirred at 40 °C for 24 h. The reaction was then allowed to cool to rt, and concentrated under reduced pressure. The resulting off-white solid obtained was washed with Et_2O (100 mL) and purified by flash column chromatography (0-7.5% MeOH:CH_2Cl_2, R_f 0.46 in 10% MeOH:CH_2Cl_2). Pure fractions were concentrated under reduced pressure to afford the title compound (0.44 g, 3.5 mmol, 77%) as a white solid with spectroscopic data in accordance with the literature.[9] **^{1}H NMR** (400 MHz, $CDCl_3$) $\delta_{H:}$ 4.81 (2H, s, -CH$_2$), 7.24-7.42 (3H, m, ArH), 8.25 (1H, ddd, *J* = 6.3, 0.9, 0.9 Hz, ArH); **mp** 115-119 °C {Lit.[9] 130-132 °C}.

2-formylpyridine 1-oxide (13)

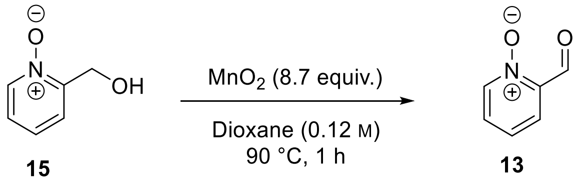

15

13
47%

MnO_2 (0.91 g, 10.4 mmol, 8.7 equiv.) was added to a solution of compound **15** (0.15 g, 1.2 mmol, 1.0 equiv.) in 1,4-dioxane (10 mL, 0.12 M) and the reaction mixture was stirred at 90 °C for 1 h. The still hot reaction mixture was filtered through Celite and washed with hot 1,4-dioxane (100 mL). The filtrate was concentrated under reduced pressure and the resulting yellow solid was purified by flash column chromatography (0-5% MeOH:CH_2Cl_2, R_f 0.33 in 10% MeOH:CH_2Cl_2). Pure fractions were concentrated under reduced pressure to afford the title compound (69 mg, 0.6 mmol, 47%) as a pale-yellow solid with spectroscopic data in

accordance with the literature.[10] **^{1}H NMR** (400 MHz, $CDCl_3$) $\delta_{H:}$ 7.33 (1H, dd, $J_1 = J_2$ = 7.7 Hz, ArH5), 7.46 (1H, ddd, J = 7.7, 6.5, 2.2 Hz, ArH4), 7.82 (1H, dd, J = 7.7, 2.2 Hz, ArH6), 8.21 (1H, d, J = 6.5 Hz, ArH3), 10.64 (1H, s, COH); **mp** 54-57 °C {Lit.[11] 63-65 °C}.

(c) *Amino acid* ***14***

***Tert*-butyl-(*S*)-(1-(dimethylamino)-1-oxopropan-2-yl)carbamate (16)**

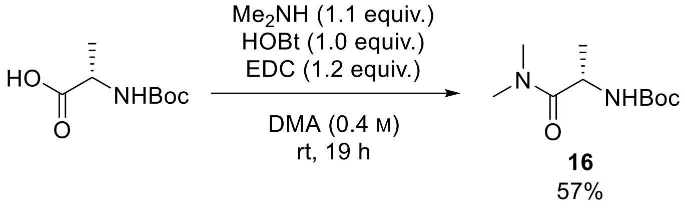

Dimethylamine (1.1 mL, 8.7 mmol, 1.1 equiv., 40 wt.% in H_2O) was added to a solution of Boc-Ala-OH (1.50 g, 7.9 mmol, 1.0 equiv.) in DMA (20 mL, 0.4 M). HOBt (1.07 g, 7.9 mmol, 1.0 equiv.) was added and the reaction mixture was cooled to 0 °C. EDAC (1.52 g, 9.8 mmol, 1.2 equiv.) was then added at 0 °C, and the reaction mixture was allowed to warm to rt and stirred for 19 h. The reaction mixture was then added to H_2O (20 mL) and extracted with EtOAc (3 × 40 mL). The organic layers were combined, washed with H_2O (20 mL) and saturated brine (20 mL), dried over $MgSO_4$, filtered, and concentrated. The resulting colourless liquid was purified by flash column chromatography (10-50% EtOAc in Petrol, R_f 0.24, visualised using ninhydrin stain) and pure fractions were concentrated under reduced pressure to obtain the title compound as a colourless liquid (0.98 g, 4.5 mmol, 57%) with spectroscopic data in accordance with the literature.[12] **^{1}H NMR** (400 MHz, $CDCl_3$) $\delta_{H:}$ 1.26 (3H, d, J = 7.0 Hz, CHMe), 1.39 (9H, s, Boc), 2.93 (3H, s, NMe), 3.02 (3H, s, NMe), 4.59 (1H, dq, $J_1 = J_2$ = 7.0 Hz, CHMe), 5.49 (1H, d, J = 7.0 Hz, NHBoc).

(*S*)-2-amino-*N*,*N*-dimethylpropanamide hydrogen chloride (17)

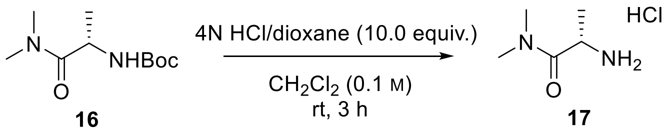

Hydrochloric acid (5.8 mL, 23.1 mmol, 10.0 equiv., 4N in dioxane) was added to a solution of compound **16** (0.50 g, 2.3 mmol, 1.0 equiv.) in CH_2Cl_2 (23 mL, 0.1 M) and the reaction mixture was stirred at rt for 3 h. The solvent was removed under reduced pressure and the resulting residue was azeotroped with CH_2Cl_2 (25 mL). The white solid obtained (0.38 g) was used immediately in the subsequent reaction without characterisation.

(*S*)-2-amino-*N*,*N*-dimethylpropanamide (14)

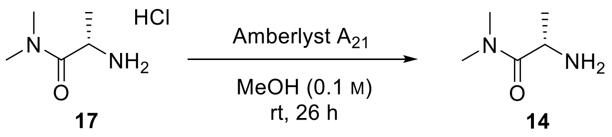

Amberlyst A_{21} (2.00 g) was added to a solution of compound **17** (0.21 g, 1.4 mmol, 1.0 equiv.) in MeOH (10 mL, 0.1 M) and the reaction mixture was stirred at rt for 26 h, followed by filtration through celite. To remove dissolved celite, the filtrate was concentrated under reduced pressure, dissolved in CH_2Cl_2, and filtered. The resulting filtrate was concentrated under reduced pressure to obtain the title compound (0.13 g, 1.1 mmol, 87%) as a yellow liquid. **^{1}H NMR** (400 MHz, $CDCl_3$) δ_H: 1.36 (3H, d, *J* = 6.8 Hz, CHMe), 2.95 (3H, s, NMe), 3.04 (3H, s, NMe), 4.10 (1H, br s, CHMe), 4.35 (2H, br s, NH).

2. Hydrate, imine and imidazolidinone formation

(a) Hydrate formation

^{1}H NMR spectra of PCAs **2-13** (50 mM in 100 mM deuterated Na phosphate buffer, pD 7.3) were recorded at 37 °C to determine the ratio between aldehyde and hydrate forms. It was assumed that equilibria were reached quickly before NMR spectra were recorded. Note: due to reduced quantities of material available, the data for 2-PCA **12** was collected at a reduced concentration of 15 mM. The concentration of aldehyde [**PCA**] and hydrate [**Hy**] were calculated *via* the relative integrals of diagnostic ^{1}H NMR signals outlined in **Fig. S3*a***. The equilibrium constant for hydrate formation (**K_1 (Hy)**) was calculated using **Equation S1**:

$$K_1\ (\mathbf{Hy}) = \frac{[\mathbf{Hy}]}{[\mathbf{PCA}]} \qquad \textbf{(Equation S1)}$$

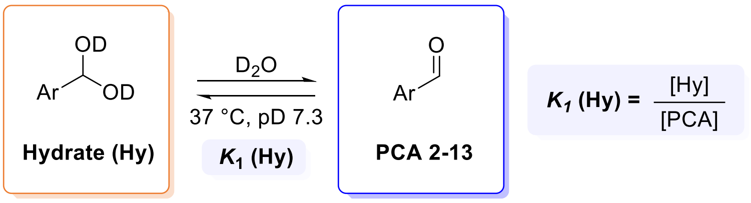

PCA	Aldehyde	Hydrate	*K₁* (Hy)
**2**	69%	31%	0.44
**3**	97%	3%	0.03
**4**	76%	24%	0.31
**5**	99%	1%	0.01
**6**	94%	6%	0.06
**7**	71%	29%	0.41
**8**	53%	47%	0.90
**9**	90%	10%	0.11

**10**	57%	43%	0.75
**11**	24%	76%	3.17
**12**	<1%	100%	>1000
**13**	12%	88%	7.08

Table S1. Hydrate/aldehyde equilibria (initial PCA concentration: 50 mM; 15 mM for PCA **12**).

(b) *Imine formation*

An aliquot of each PCA/hydrate solution (300 μL, 50 mM, 15 μmol, 1 equiv., in 100 mM deuterated Na phosphate buffer, pD 7.3) was added to a solution of dimethyl amide **14** (300 μL, 50 mM, 15 μmol, 1 equiv., in 100 mM deuterated Na phosphate buffer, pD 7.3) and the reaction mixtures were incubated at 37 °C for up to 48 h. ^{1}H NMR spectra were recorded at 37 °C, at the time-points indicated in **Table S2**. At this point, equilibrium had been reached in all cases. Note: due to the limited quantity of material available, the data for 2-PCA **12** was collected at a reduced concentration; an aliquot of the PCA **12**/hydrate solution (150 μL, 15 mM, 2.3 μmol, 1 equiv., in 100 mM deuterated Na phosphate buffer, pD 7.3) was added to dimethyl amide **14** (150 μL, 15 mM, 2.3 μmol, 1 equiv., in 100 mM deuterated Na phosphate buffer, pD 7.3).

The concentration of aldehyde $[\mathbf{PCA}]$, hydrate $[\mathbf{Hy}]$ and imine $[\mathbf{Im}]$ species were calculated *via* the relative integrals of diagnostic ^{1}H NMR signals outlined in **Fig. S3*b***. The concentration of unreacted amino acid **14** was calculated using **Equation S2**, where $[\mathbf{13}]_{t=0}$ is the starting concentration of **14**.

$$[\mathbf{14}] = [\mathbf{14}]_{\boldsymbol{t=0}} - [\mathbf{Im}] \qquad \textbf{(Equation S2)}$$

The equilibrium constant for imine formation (***K*₂ (Im)**) was then calculated using **Equation S3**:

$$\boldsymbol{K_2}\ (\mathbf{Im}) = \frac{[\mathbf{Im}]}{[\mathbf{PCA}][\mathbf{14}]} \qquad \textbf{(Equation S3)}$$

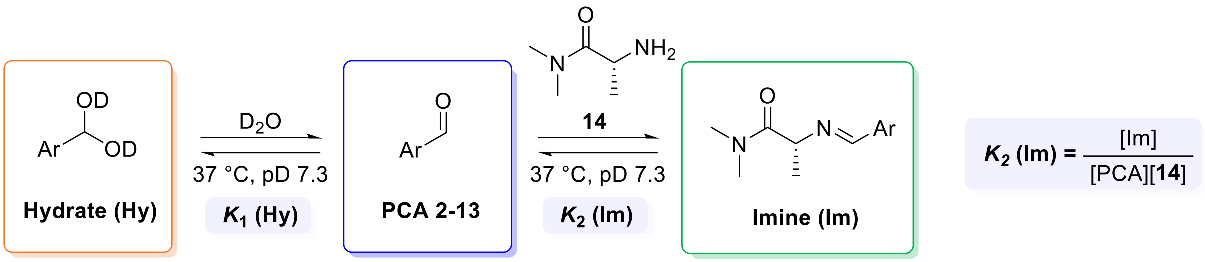

PCA	Aldehyde	Hydrate	Imine	K_2 (Im) / M^{-1}
**2**	63%	28%	9%	6.28
**3**	76%	2%	22%	14.65
**4**	69%	23%	8%	5.04
**5**	99%	1%	<1%[b]	-
**6**	90%	5%	5%	2.37
**7**	61%	25%	14%	10.67
**8**	48%	40%	12%	11.36
**9**	82%	6%	12%	6.65
**10**	51%	37%	12%	10.70
**11**	19%	70%	11%	26.02
**12**	<1%[b]	100%	<1%[b]	-
**13**[a]	<1%[b]	85%	15%	8.30[a]

Table S2. Hydrate/aldehyde equilibria (initial PCA/amino acid **14** concentration: 25 mM; 7.5 mM for PCA **12**). [a] For PCA **13**, the absence of aldehyde made it impossible to calculate $\boldsymbol{K_2}$ directly. Instead, $\boldsymbol{K_{obs}}$ is given for the equilibrium between hydrate and imine. [b] Levels were below the detection sensitivity of the measurement.

(c) Imidazolidinone formation

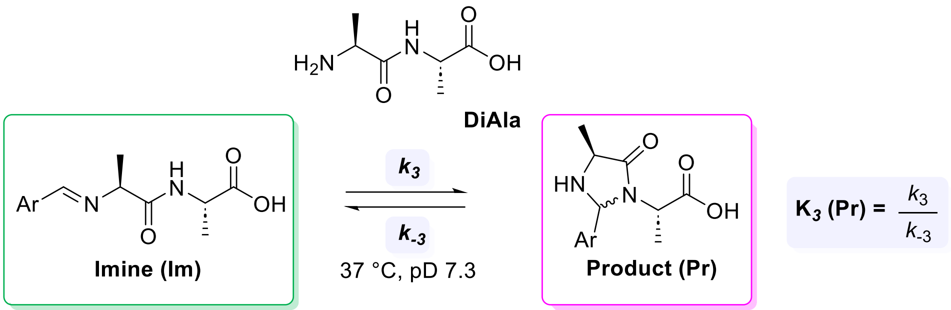

A solution of DiAla (150 µL, 100 mM, 15 µmol, 1 equiv.) was added to solutions of reagents **2-13** (150 µL, 100 mM, 15 µmol, 1 equiv.), both in deuterated sodium phosphate buffer (100 mM, pD 7.3). The reactions were incubated at 37 °C for 16 h and conversion was followed by ^{1}H NMR spectroscopy at 30 min intervals.

At each timepoint ($t = x\ h$), the concentration of aldehyde $[\mathbf{PCA}]$, hydrate $[\mathbf{Hy}]$, imine $[\mathbf{Im}]$ and sum of imidazolidinone product $[\mathbf{Pr}]$ species were calculated *via* the relative integral ratios of the diagnostic ^{1}H NMR signals outlined in **Fig. S3c**. Concentration values were normalised using a correction factor (CF) in **Equation S5** to remove background noise from the imidazolidinone signals, based on the assumption that the initial rate was linear over the first 3 time-points collected, and that $[\mathbf{Pr}]_{t=0\ h} = 0$. Correction factors were calculated

as the y-intercept of the linear regression line of the plot of the sum of integrals of product diastereomers over time (for the first 3 time-points collected), as demonstrated in **Fig. S1** for PCA **5**.

$$\mathbf{Conversion} = \mathbf{100} \times \frac{\mathbf{Pr} - \mathbf{CF}}{\mathbf{PCA} + \mathbf{Hy} + \mathbf{Im} + \mathbf{Pr} - \mathbf{CF}}$$ **(Equation S5)**

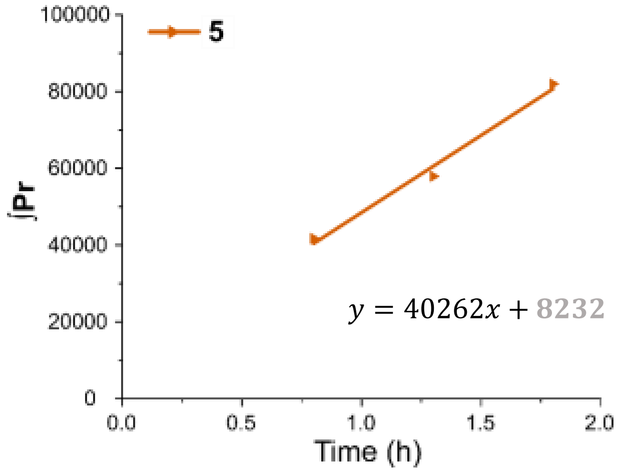

Figure S1. Example calculation of correction factor (CF) for PCA **5**. Fit is a linear regression, based on the initial rates model.

Data were fit to a multi-step reversible kinetic model in Copasi 4.34.251, encompassing hydrate, imine, and imidazolidinone formation, to estimate values of k_3 and k_{-3}.

$$\text{Hydrate} \overset{K_1\ (\text{Hy})}{\rightleftharpoons} \text{Aldehyde} \underset{\text{Amine}}{\overset{K_2\ (\text{Im})}{\rightleftharpoons}} \text{Imine} \underset{k_{-3}}{\overset{k_3}{\rightleftharpoons}} \text{Imidazolidinone}$$

Since the rates of reversible hydrate and imine formation (k_1/k_{-1} and k_2/k_{-2}, respectively) were >4-orders of magnitude greater than k_3 and k_{-3}, the exact values of these parameters do not affect imidazolidinone formation. Instead, the values of K_1 (Hy) and K_2 (Im) calculated above could be used to fix the ratio of the forward and back rates for each step since $K_x = k_x/k_{-x}$.

k_3 and k_{-3} were estimated using the evolutionary programming method built into the software, with 200 generations and a population size of 20. Parameters were restricted within the confines of: k_3 10^{-12}-10^{5} s^{-1}; k_{-3} 10^{-12}-10^{5} s^{-1}.

For PCA **13**, where no aldehyde was observed in the experiment outlined in section *2.(b)*, K_{obs} was calculated as a pseudo-equilibrium constant for the conversion of hydrate to imine. K_{obs} was built into the multi-step kinetic model described above, giving k_3 and k_{-3} values that could be compared to the values obtained for the other PCA derivatives.

$$\text{Hydrate} \underset{\text{Amine}}{\overset{K_{\text{obs}}}{\rightleftharpoons}} \text{Imine} \underset{k_{-3}}{\overset{k_3}{\rightleftharpoons}} \text{Imidazolidinone}$$

For PCA **5**, where no imine was observed in the experiment outlined in section *2.(b)*, a steady-state approximation was applied to the reaction whereby the concentration of imine was assumed to be constant throughout the reaction. The data was therefore fitted to give the *observed* second-order rate constants k_{obs4} and $k_{\text{obs-4}}$ which cannot be compared to the other rate constants generated.

$$\text{Hydrate} \overset{K_1\ (\text{Hy})}{\rightleftharpoons} \text{Aldehyde} \underset{k_{obs-4}}{\overset{\text{Amine}\ k_{obs4}}{\rightleftharpoons}} \text{Imidazolidinone}$$

For PCA **12**, where no aldehyde or imine was observed in the experiments outlined in sections *2.(a)* and *2.(b)*, a steady-state approximation was applied to the reaction whereby the concentrations of aldehyde and imine were assumed to be constant throughout the reaction. The data was therefore fitted to give the *observed* second-order rate constants k_{obs5} and $k_{\text{obs-5}}$ which cannot be compared to the other rate constants generated.

$$\text{Hydrate} \underset{k_{obs-5}}{\overset{\text{Amine}\ k_{obs5}}{\rightleftharpoons}} \text{Imidazolidinone}$$

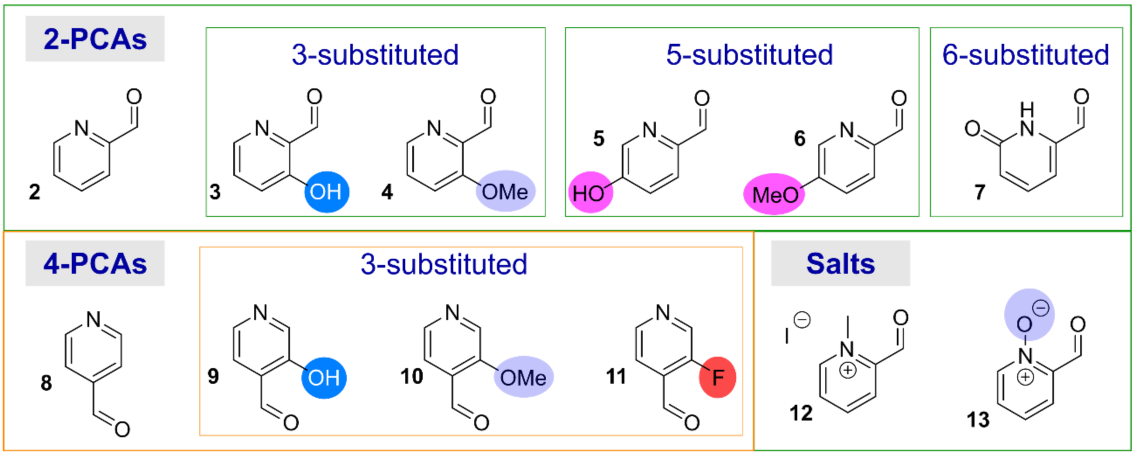

Y = +M substituent; Hydrogen bond acceptor and donor; Intramolecular general acid

Y = +M substituent; Hydrogen bond acceptor   Y = +M substituent;   Y = -I substituent;

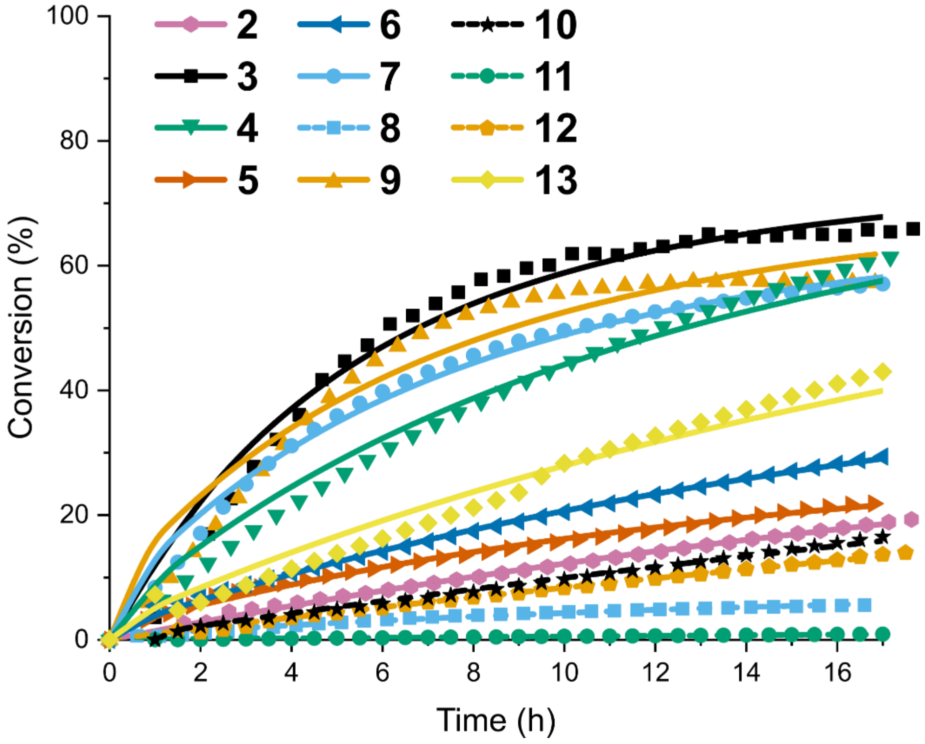

PCA	k_3 / h^{-1}	k_{-3} / h^{-1}	***K_3***
**2**	$9.3 \pm 0.09 \times 10^{-2}$	$1.2 \pm 0.1 \times 10^{-2}$	7.7
**3**	0.43 ± 0.01	$2.1 \pm 0.3 \times 10^{-2}$	19.5
**4**	0.49 ± 0.02	*	-
**5**[a]	$k_{obs4} = 0.44 \pm 0.005$ M^{-1} h^{-1}	$k_{obs\text{-}4} = 3.4 \pm 0.1 \times 10^{-2}$	12.9 M^{-1}
**6**	0.28 ± 0.02	$1.3 \pm 0.1 \times 10^{-2}$	21.6
**7**	0.42 ± 0.01	$2.2 \pm 0.2 \times 10^{-2}$	18.8
**8**	$2.8 \pm 0.07 \times 10^{-2}$	$5.4 \pm 0.4 \times 10^{-2}$	0.5
**9**	0.54 ± 0.02	$1.7 \pm 0.4 \times 10^{-2}$	31.4
**10**	$5.4 \pm 0.06 \times 10^{-2}$	*	-
**11**	$2.5 \pm 0.06 \times 10^{-3}$	*	-
**12**[a]	$k_{obs5} = 0.18 \pm 0.002$ M^{-1} h^{-1}	*	-
**13**[b]	0.14 ± 0.005	*	-

Table S3. Imidazolidinone formation (initial PCA/DiAla concentration: 50 mM). **Fitted* $k_{-3} < 10^{-12}\ s^{-1}$; [a] For PCAs **5** and **12**, data were fitted to give second-order rate constants as detailed above; [b] For PCA **13** data were fitted to a simplified model as detailed above.

3. Representative ^{1}H NMR Spectra

(a)

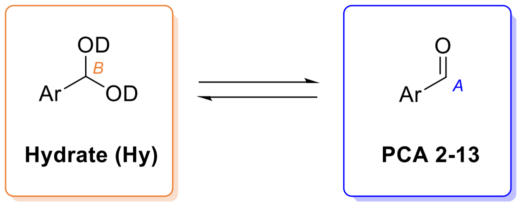

(b)

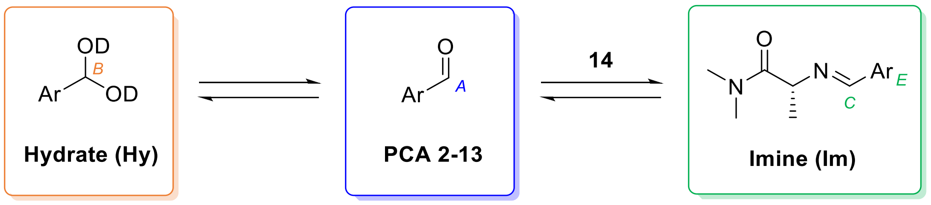

(c)

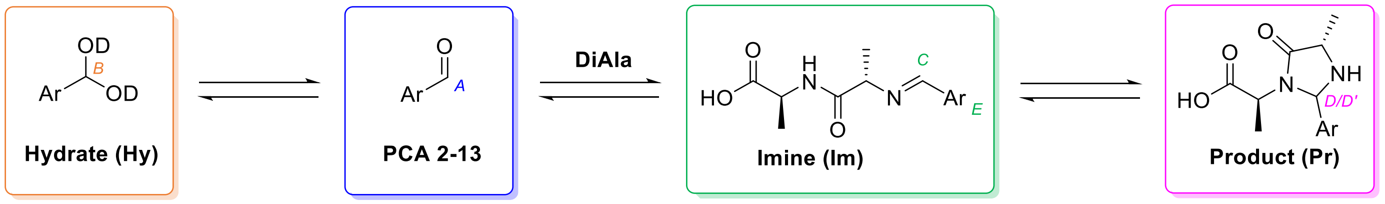

Figure S2. Overview of the diagnostic protons used to calculate: ***(a)*** the equilibrium constant for hydrate formation; ***(b)*** the equilibrium constant for imine formation; ***(c)*** rate constants for imidazolidinone formation.

PCA 2

(a)

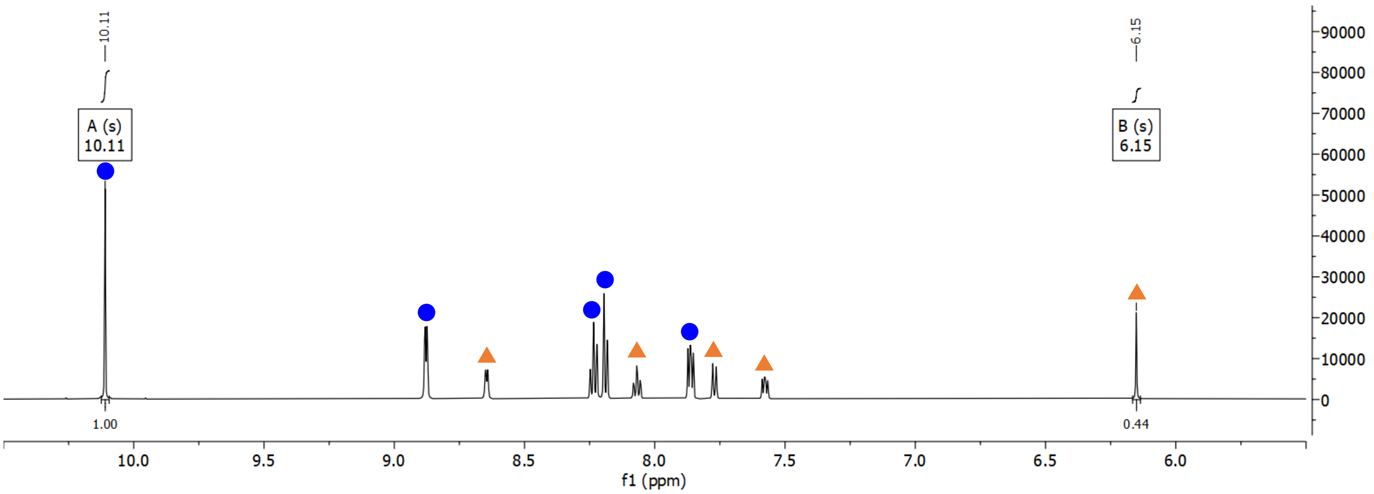

(b) (t = 1 h)

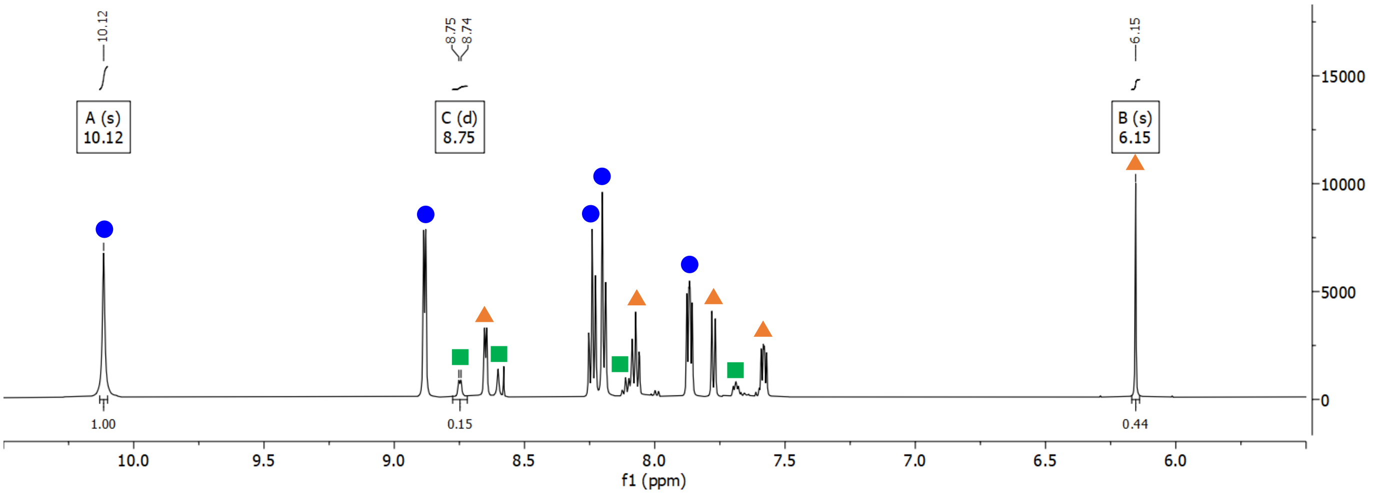

(c) (t = 17 h)

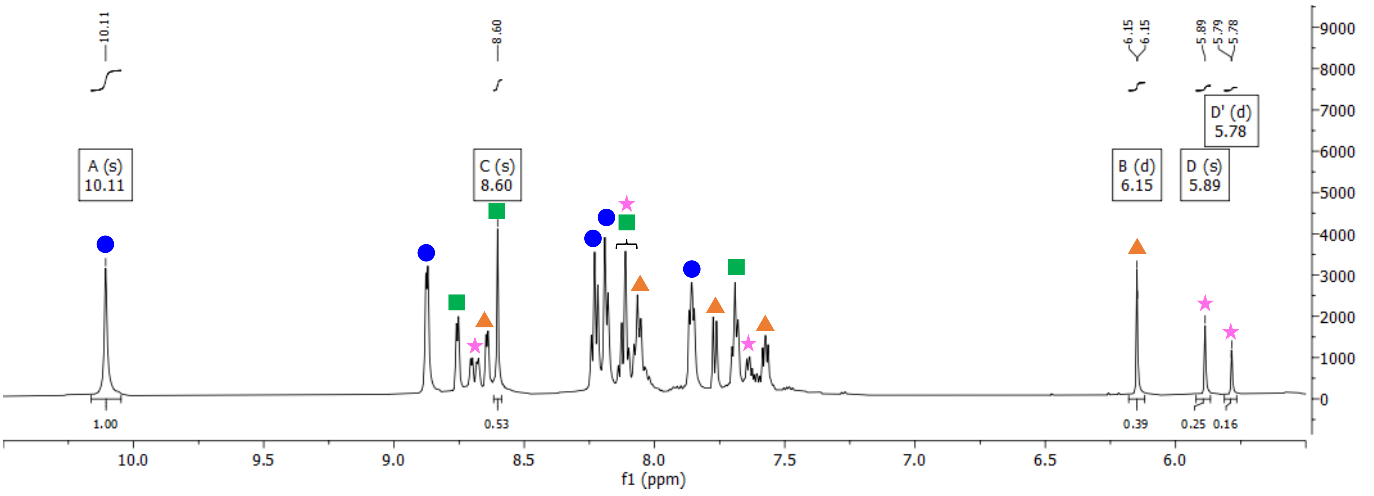

PCA 3

(a)

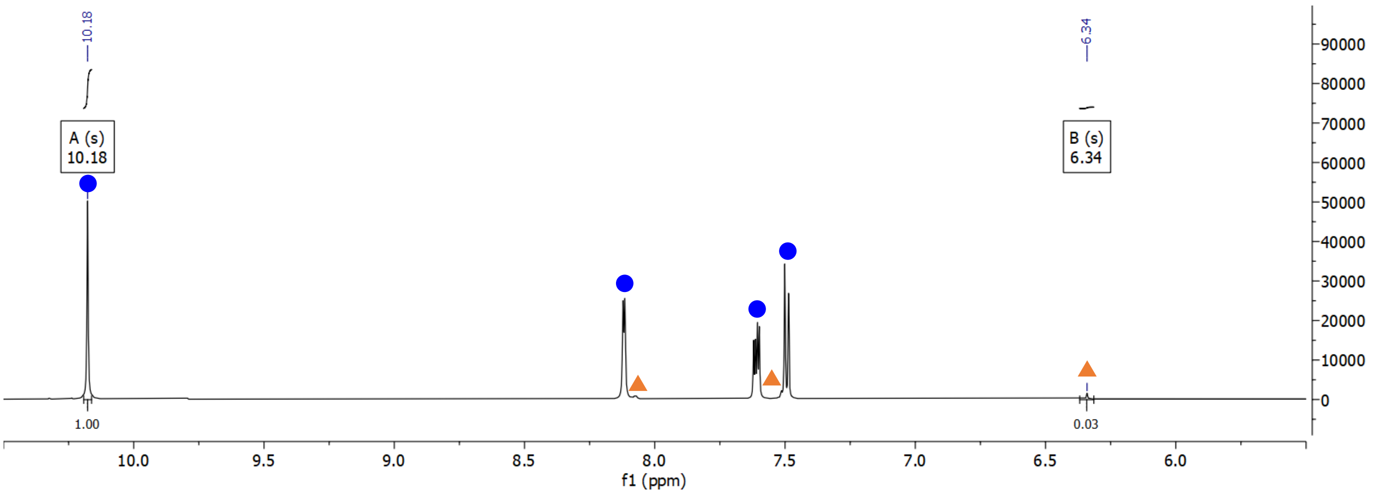

(b) (t = 1 h)

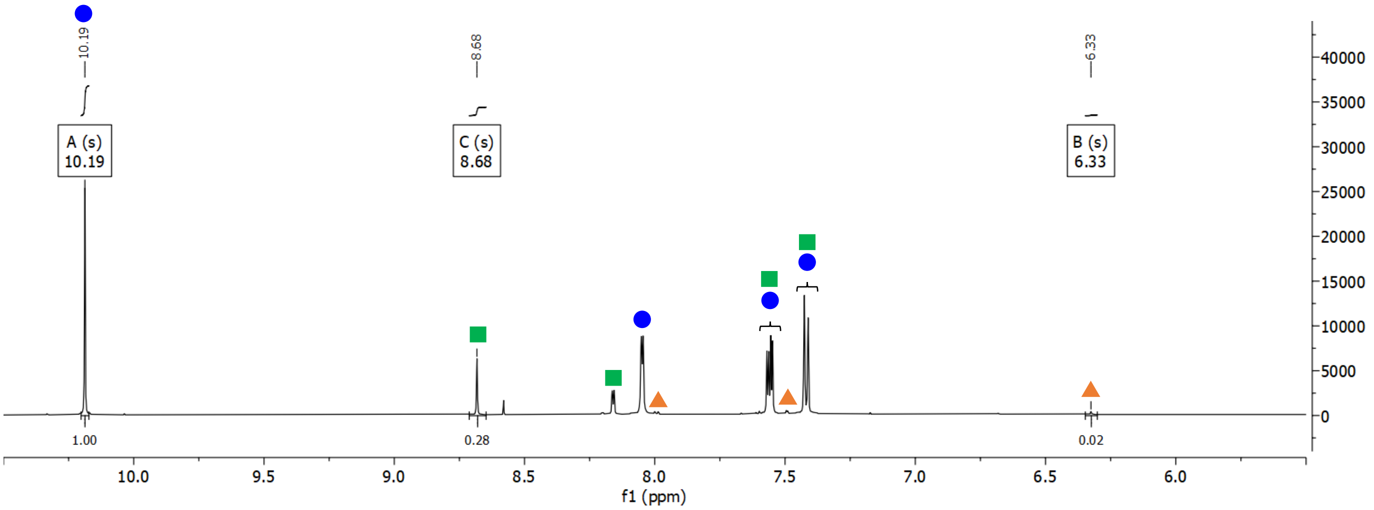

(c) (t = 17 h)

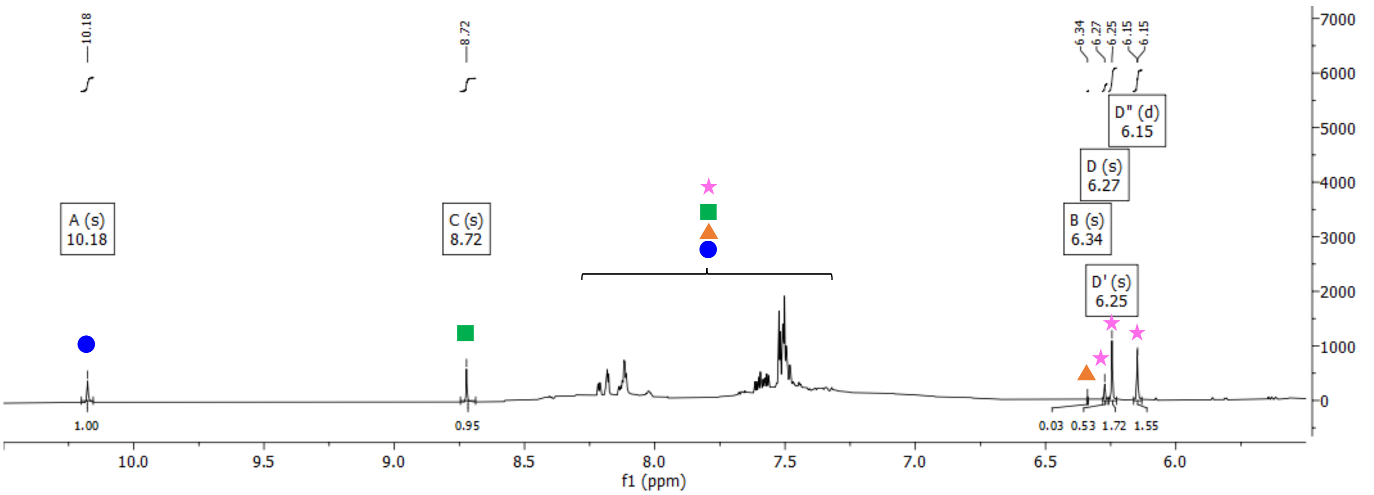

PCA 4

(a)

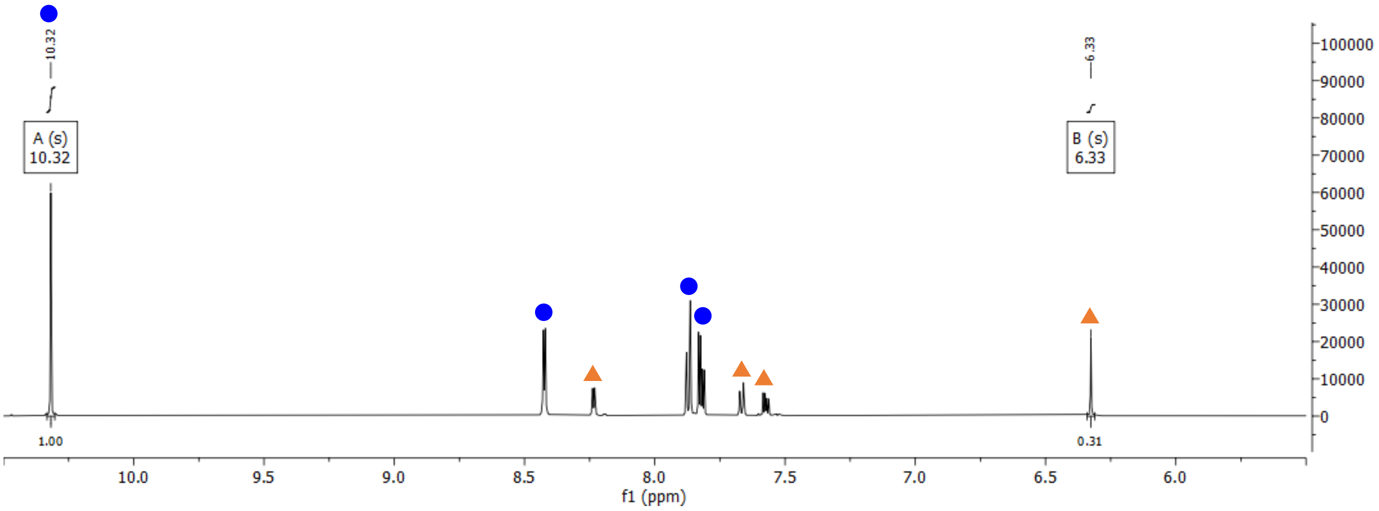

(b) (t = 1 h)

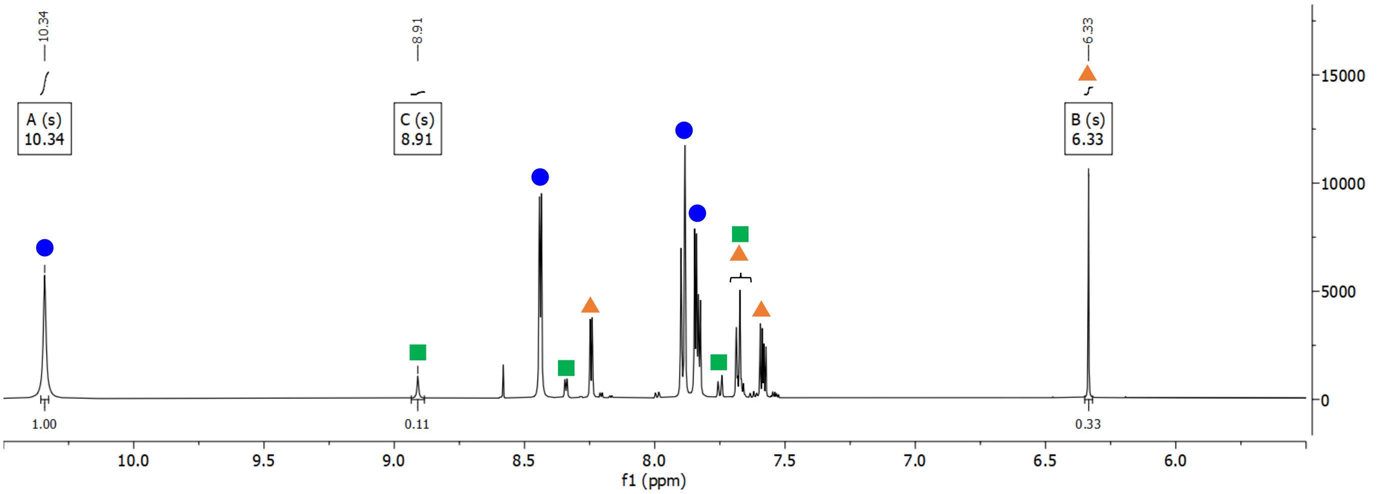

(c) (t = 17 h)

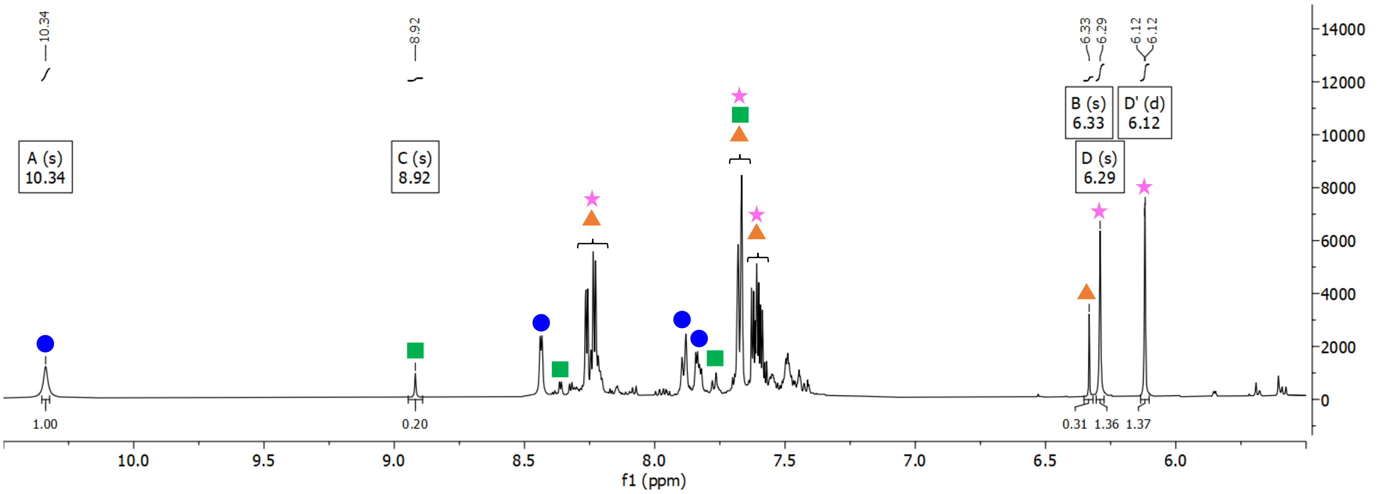

PCA 5

(a)

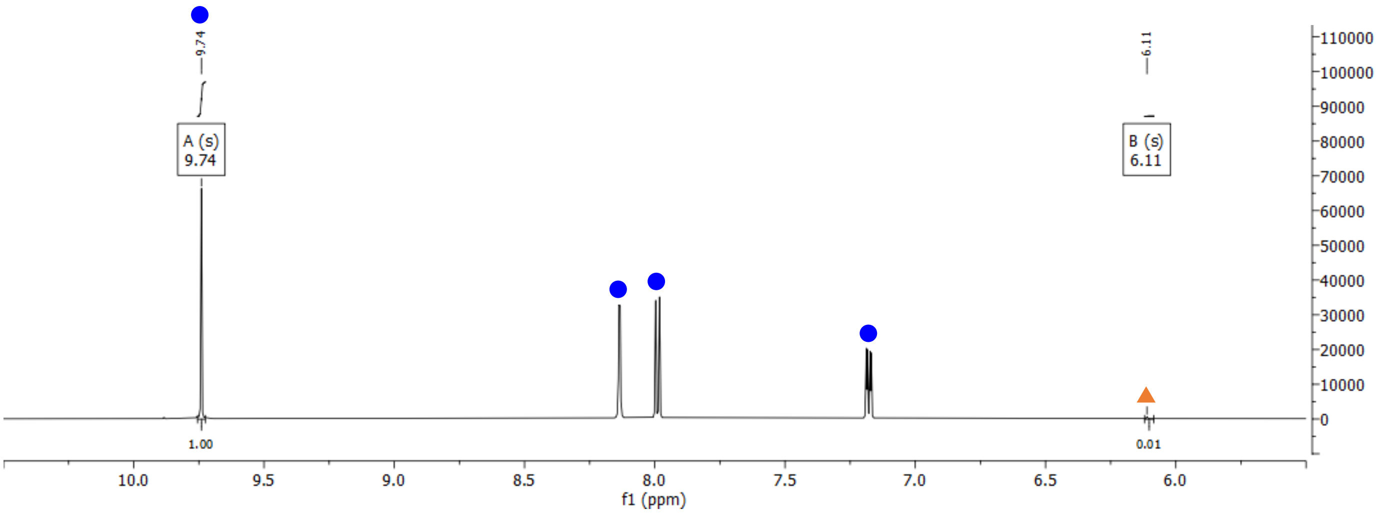

(b) (t = 1 h)

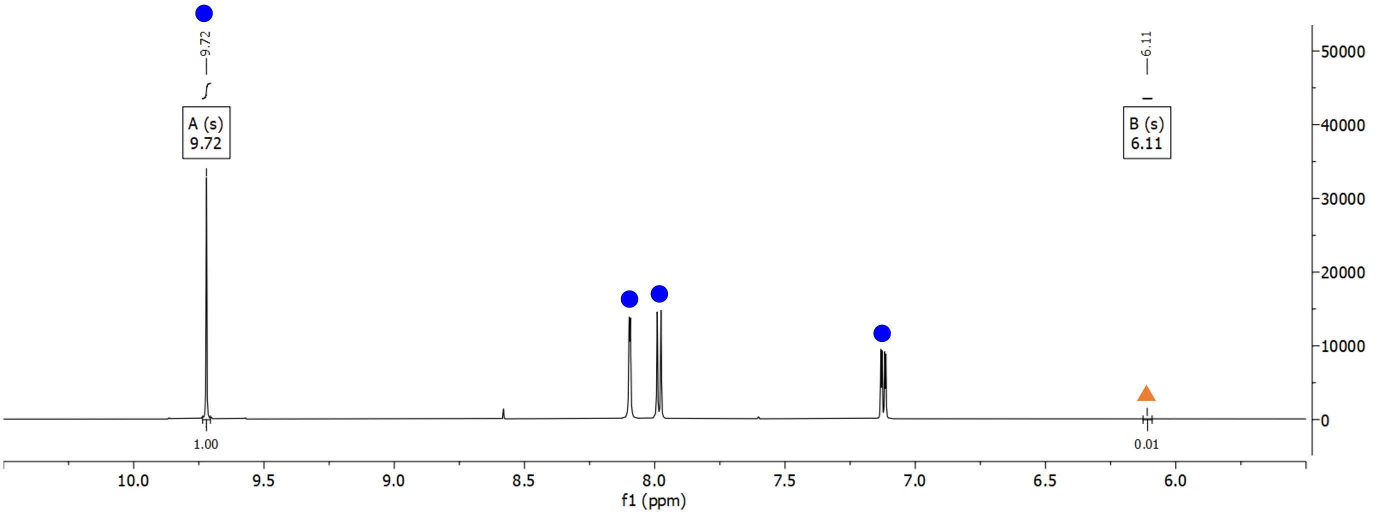

(c) (t = 17 h)

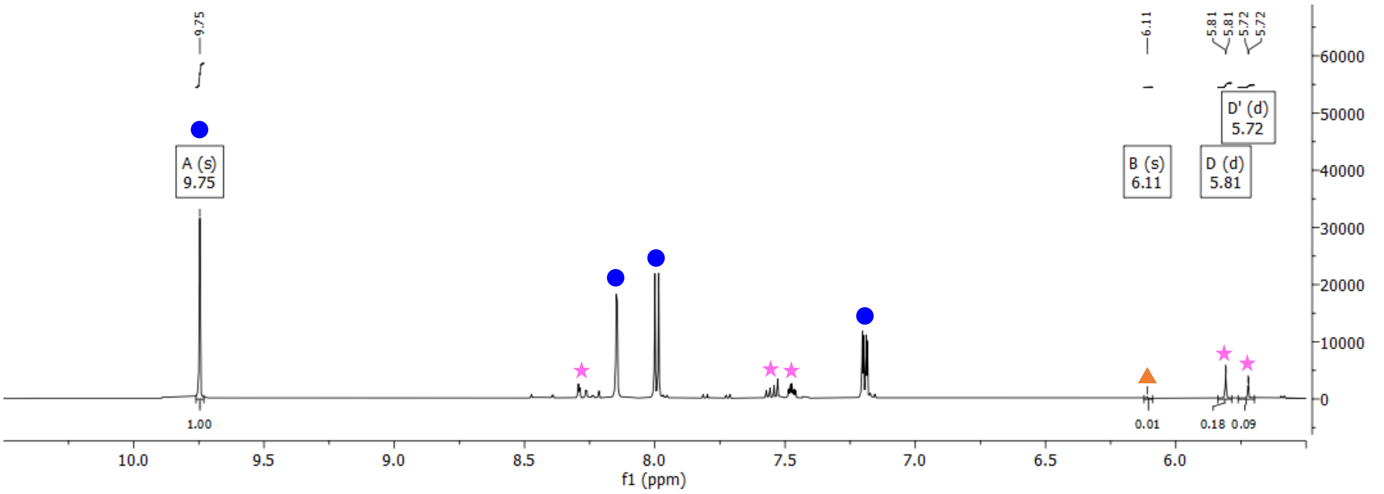

PCA 6

(a)

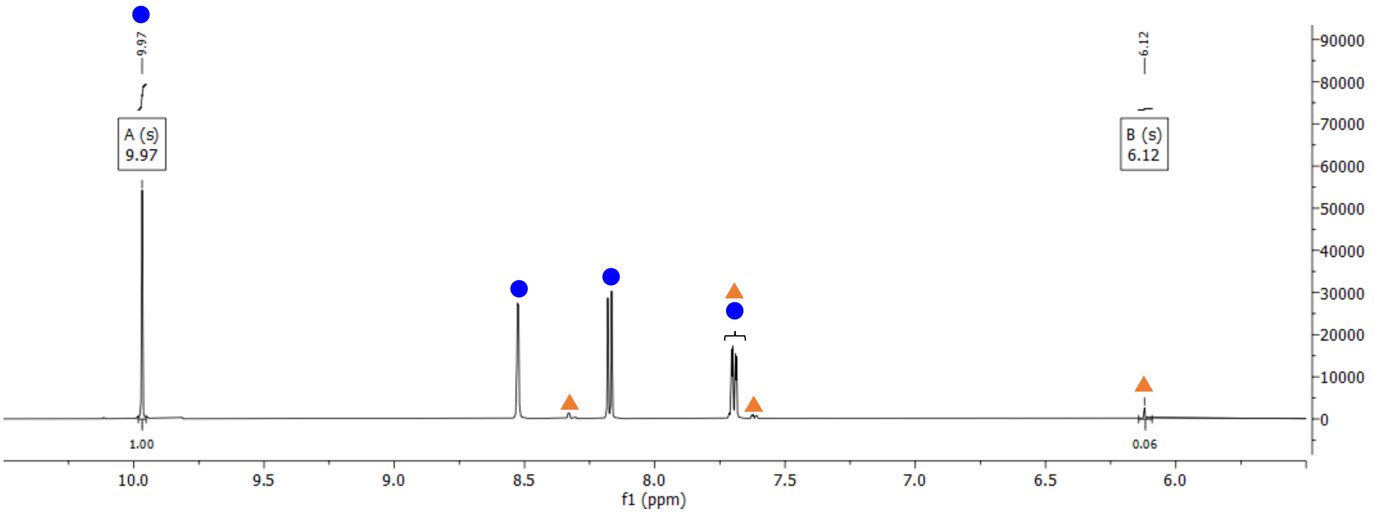

(b) (t = 1 h)

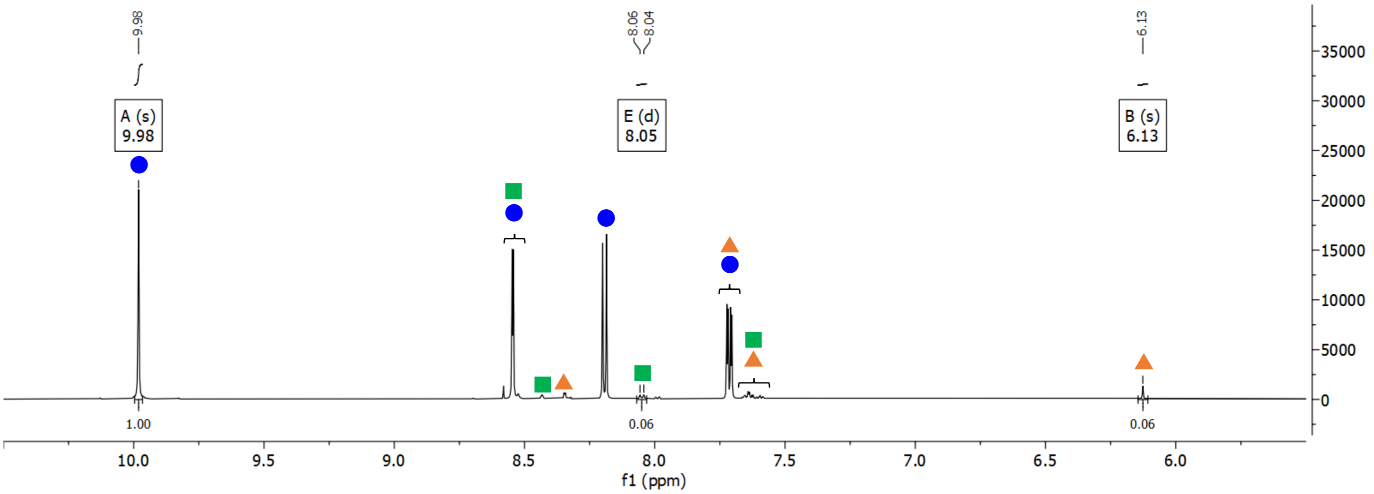

(c) (t = 17 h)

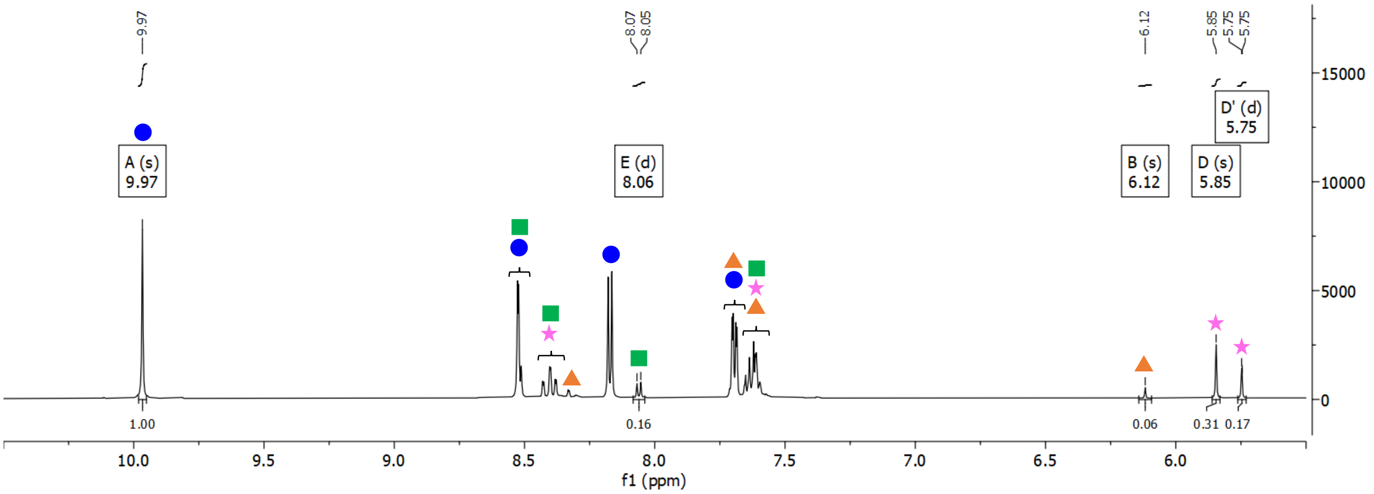

PCA 7

(a)

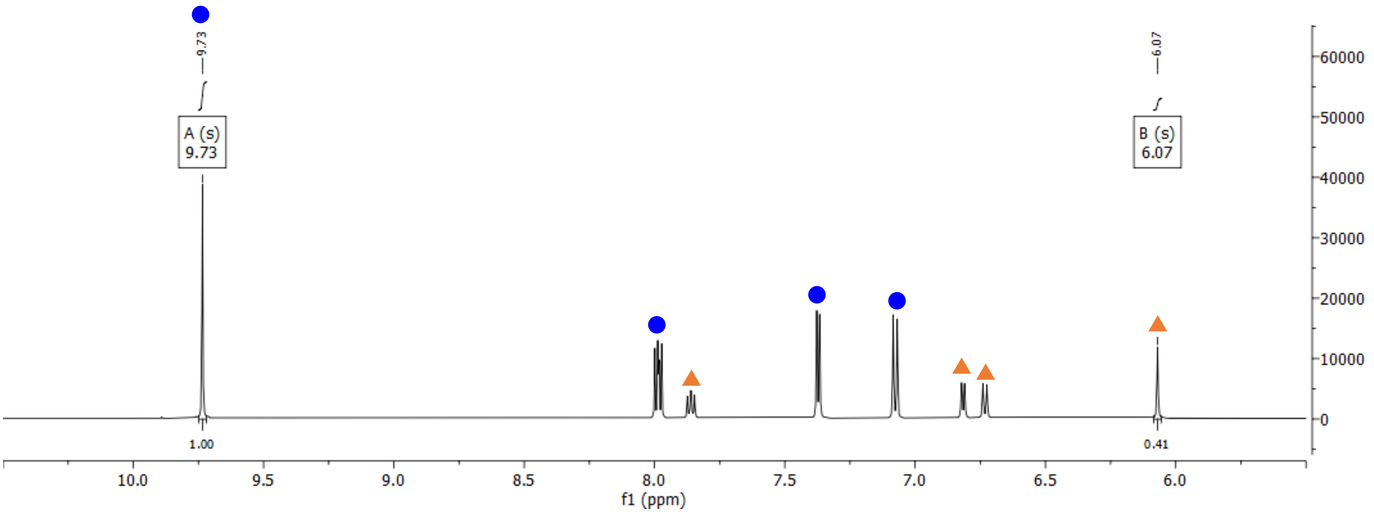

(b) (t = 1 h)

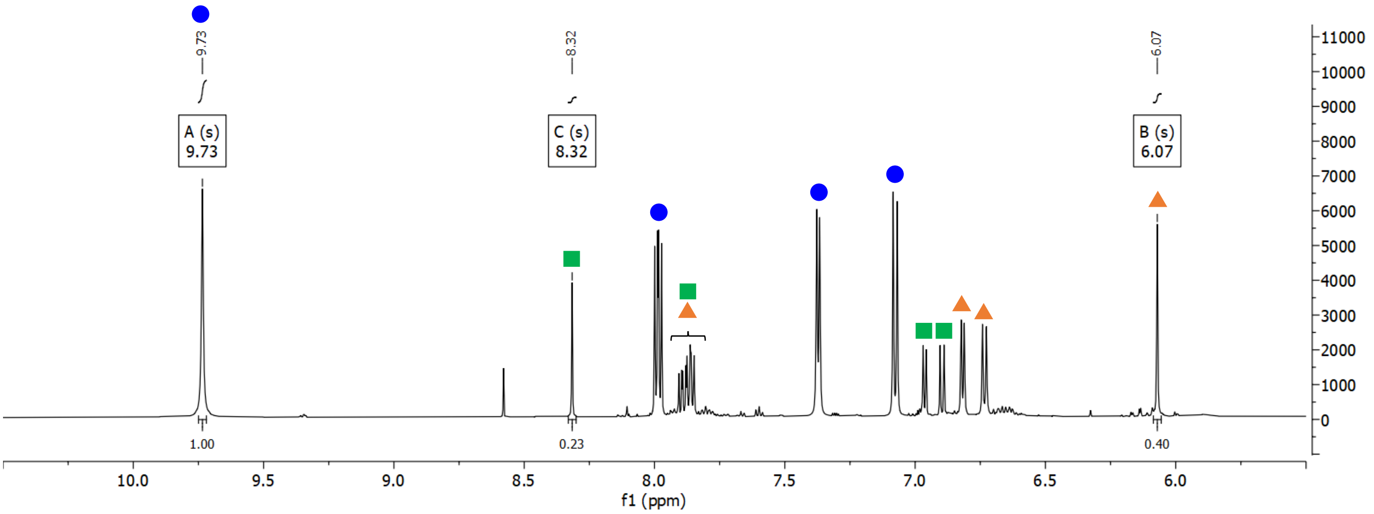

(c) (t = 17 h)

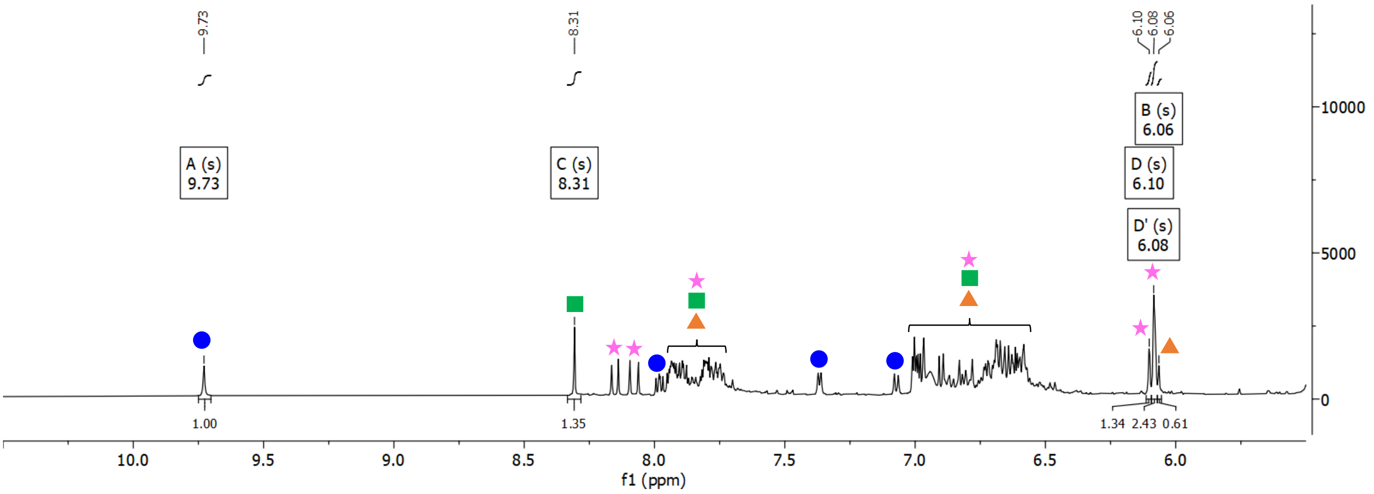

PCA 8

(a)

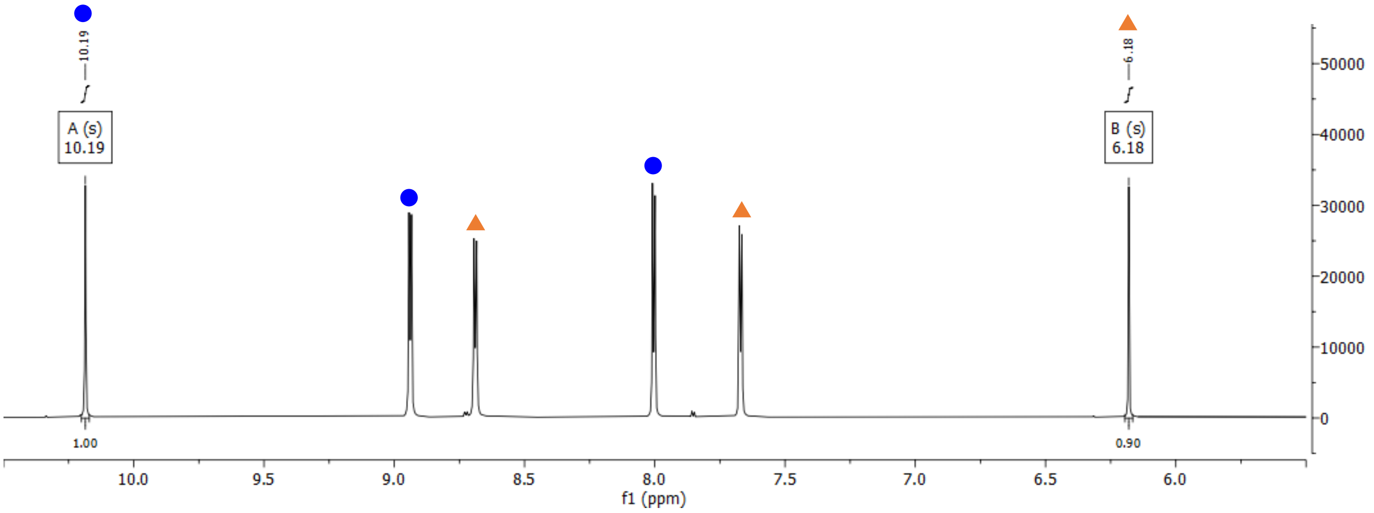

(b) (t = 1 h)

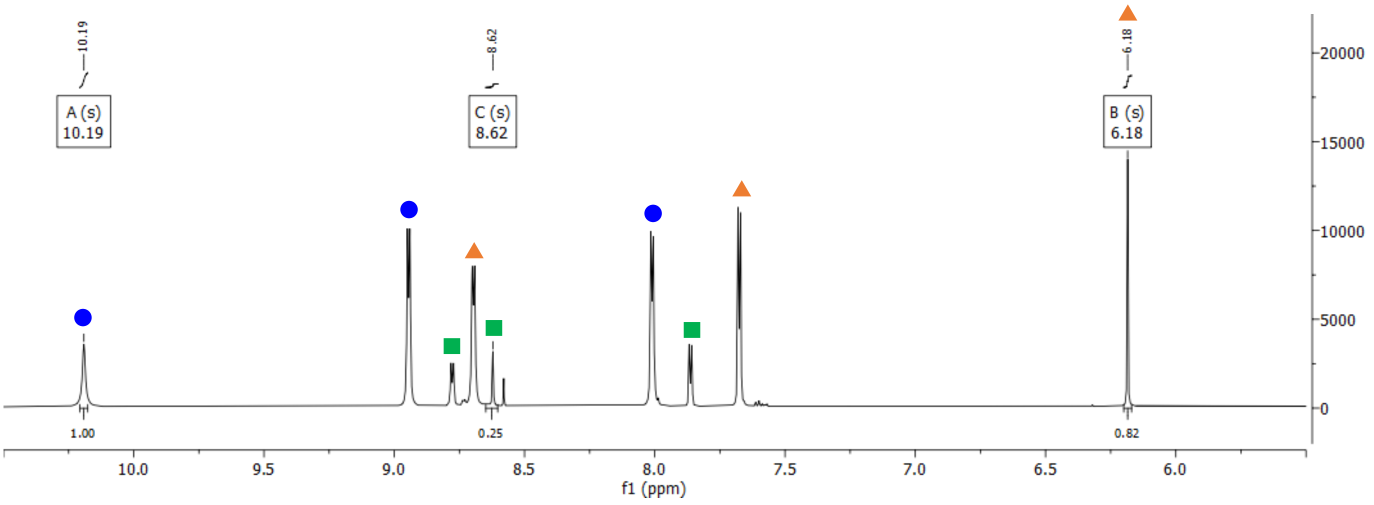

(c) (t = 17 h)

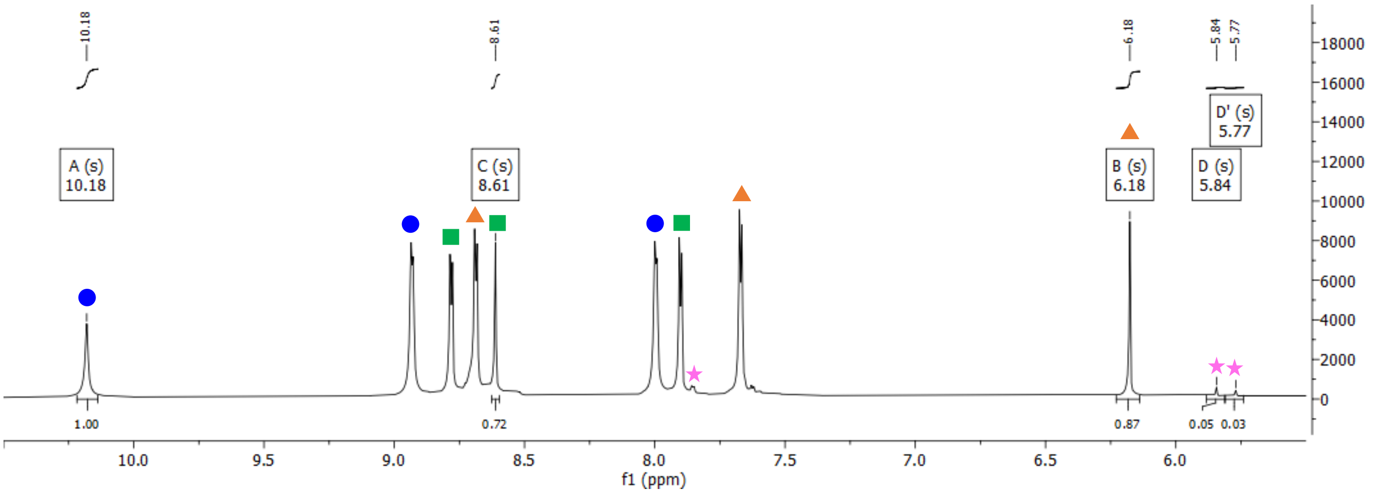

PCA 9

(a)

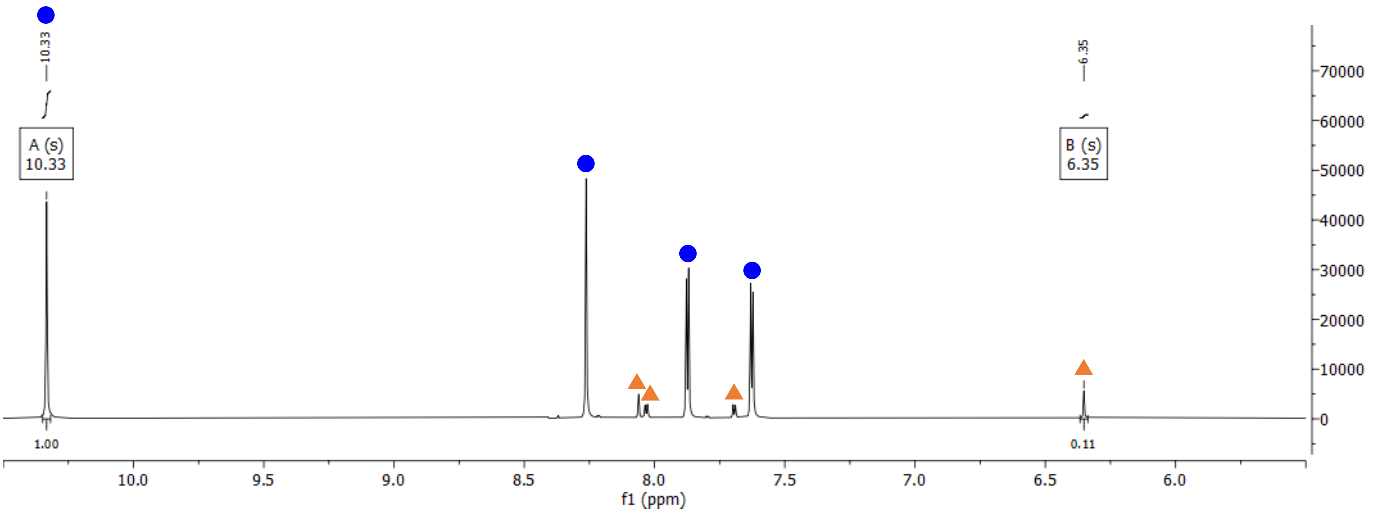

(b) (t = 17 h)

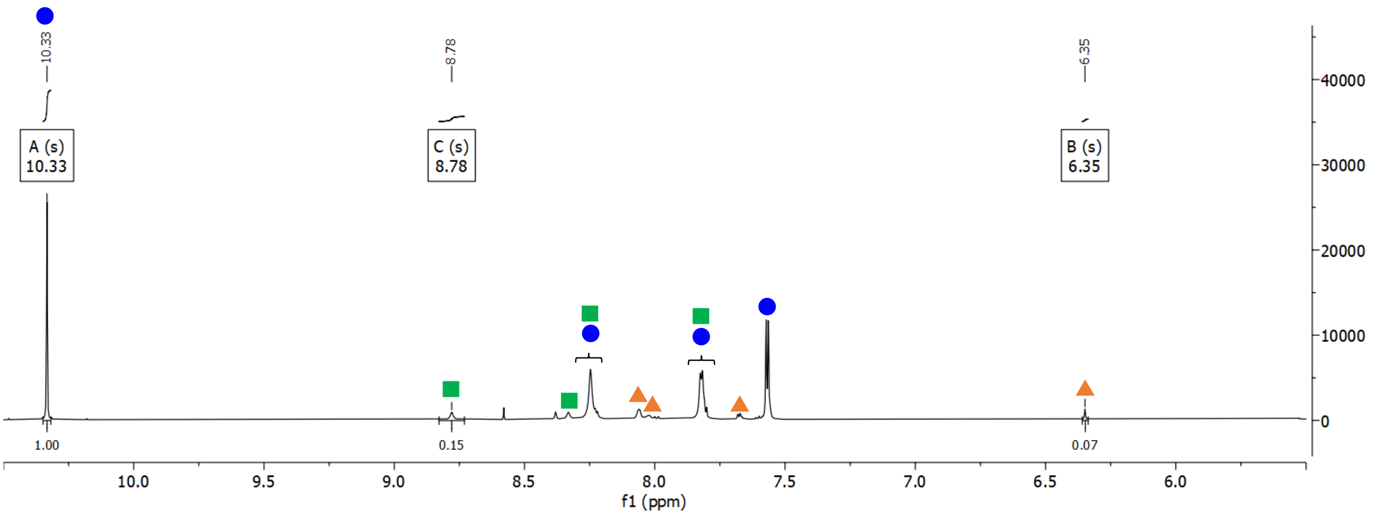

(c) (t = 17 h)

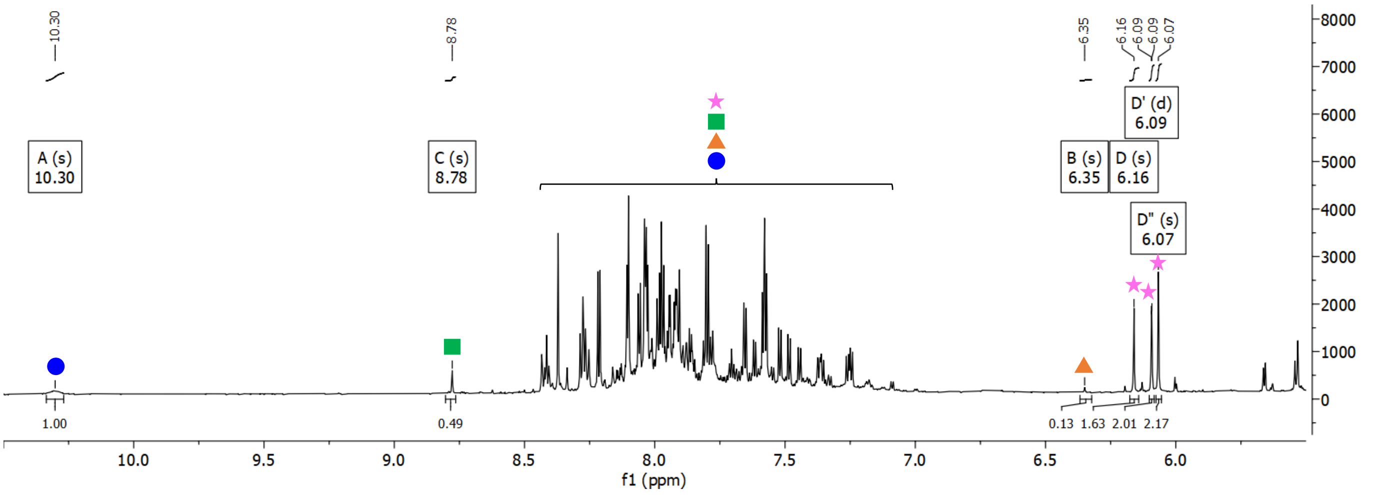

PCA 10

(a)

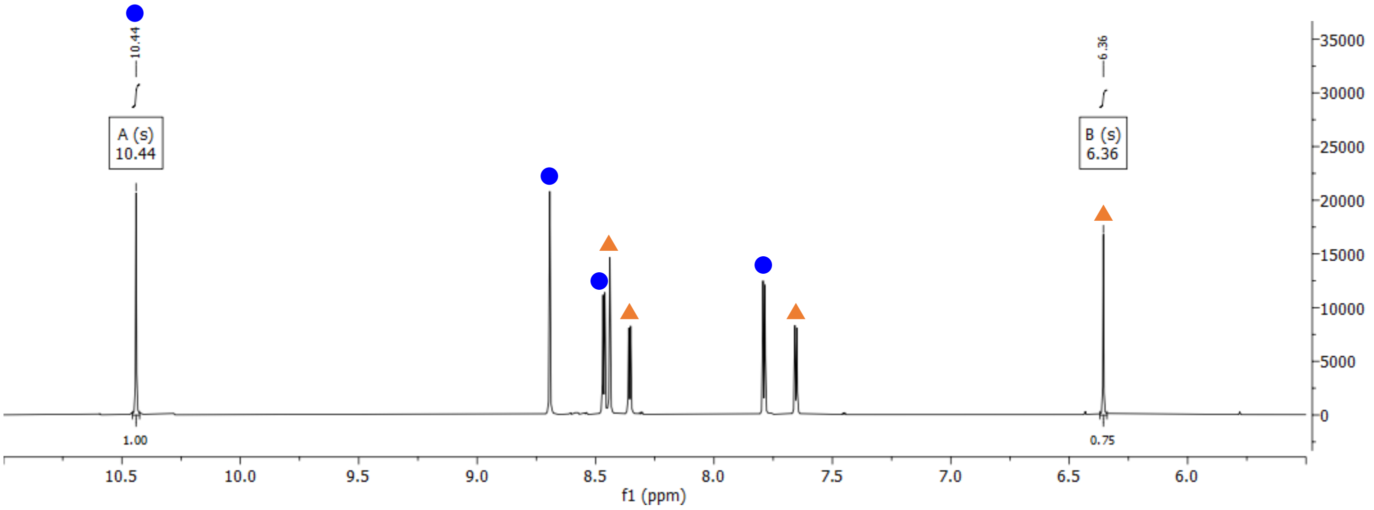

(b) (t = 1 h)

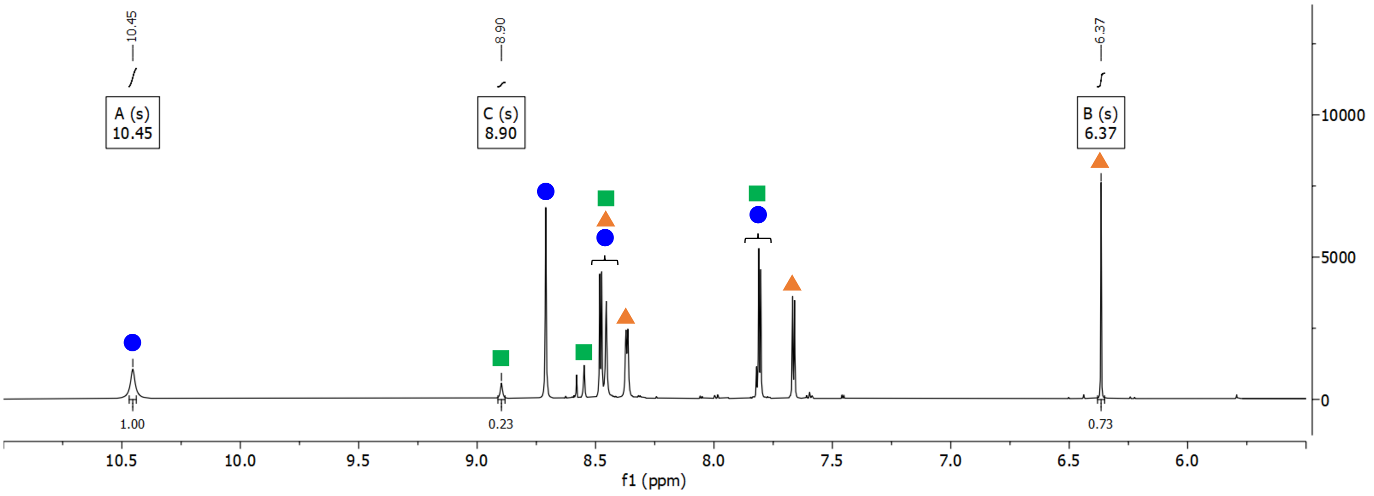

(c) (t = 17 h)

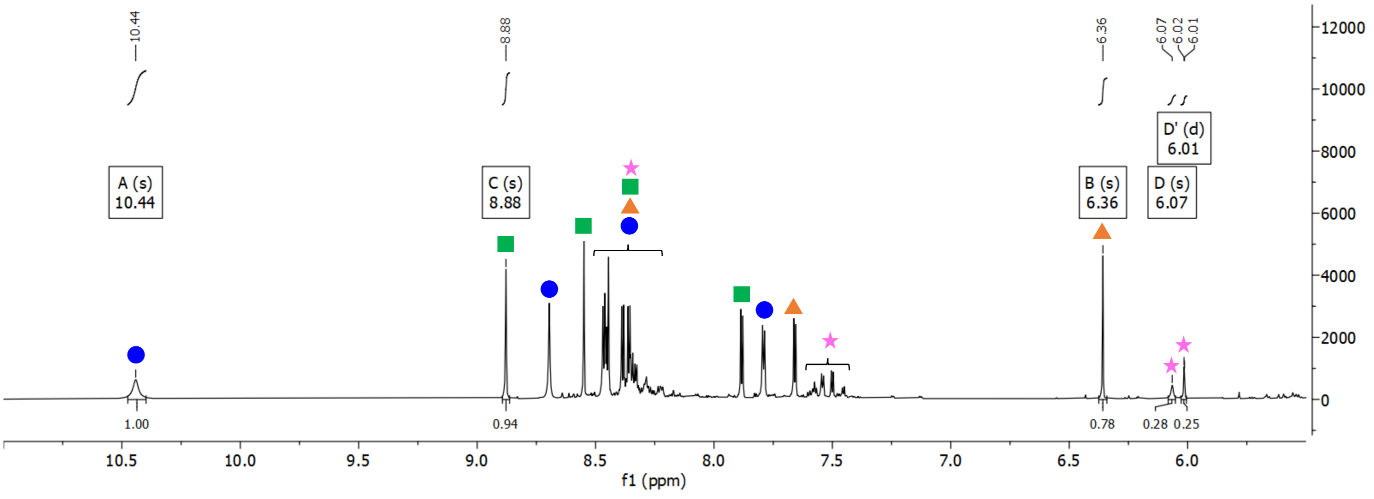

PCA 11

(a)

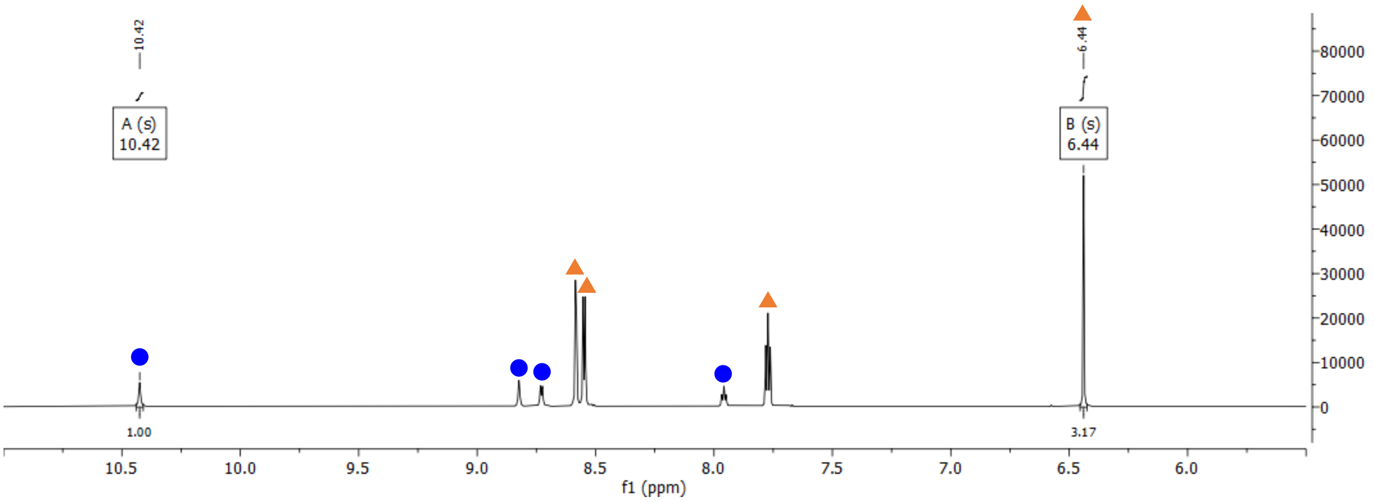

(b) (t = 1 h)

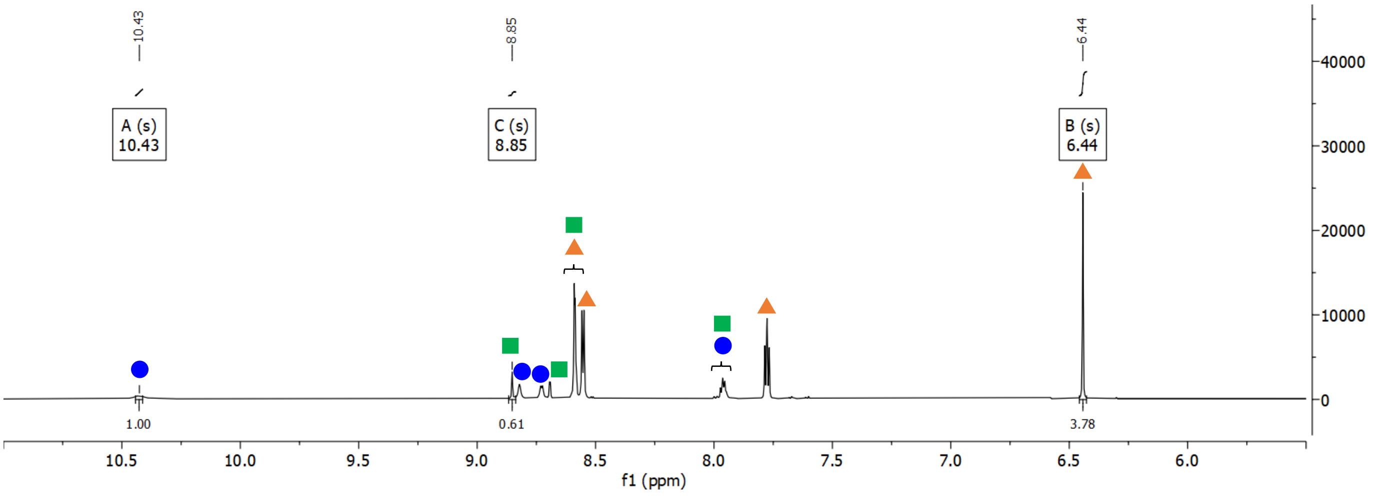

(c) (t = 17 h)

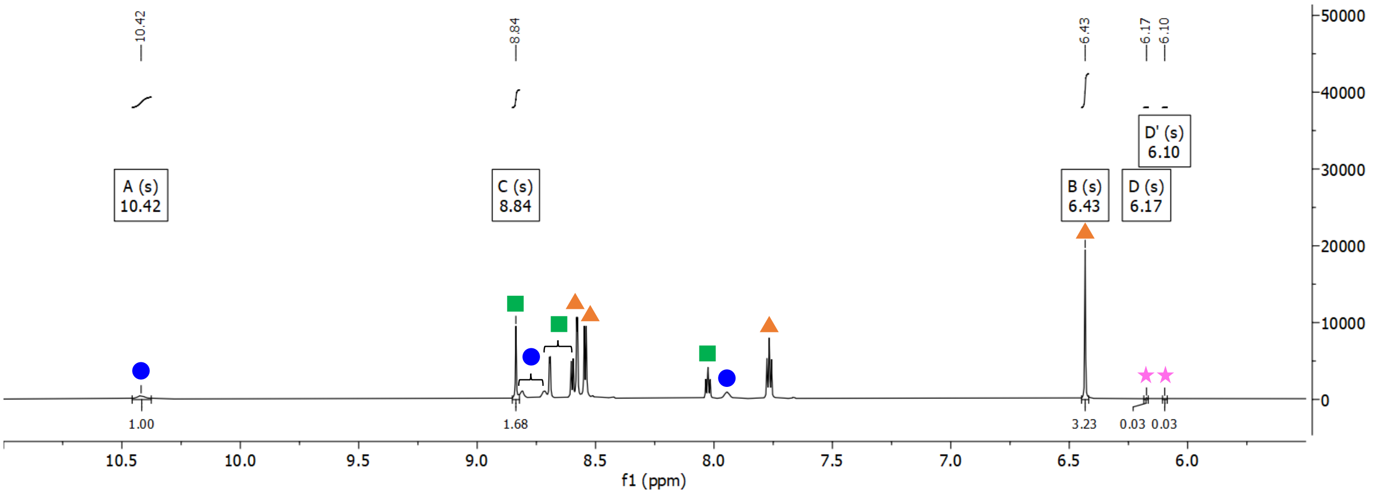

PCA 12

(a)

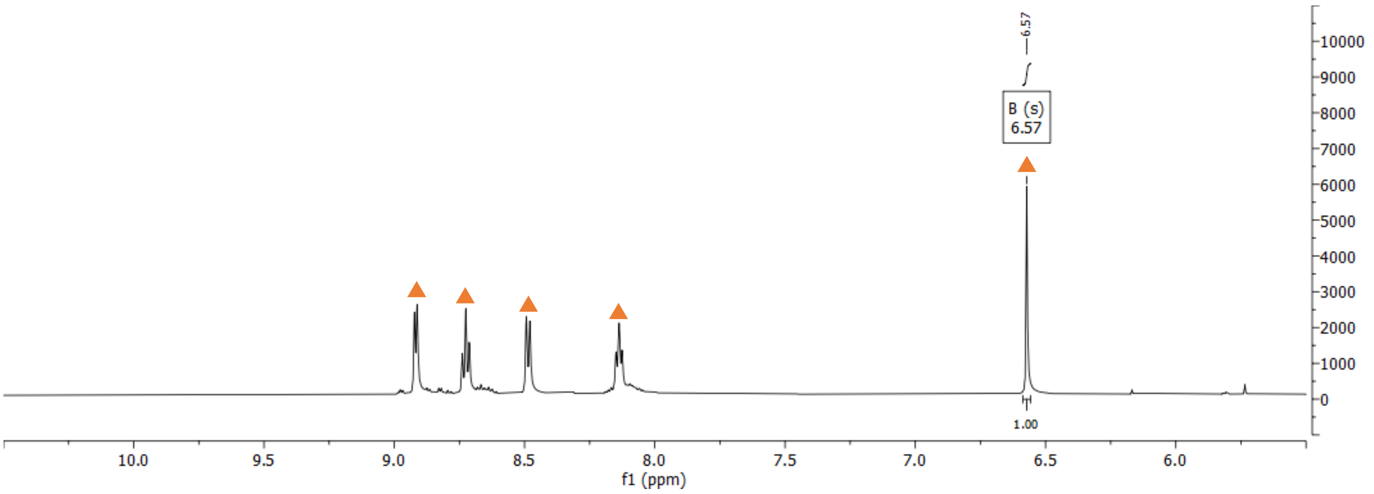

(b) (t = 18 h)

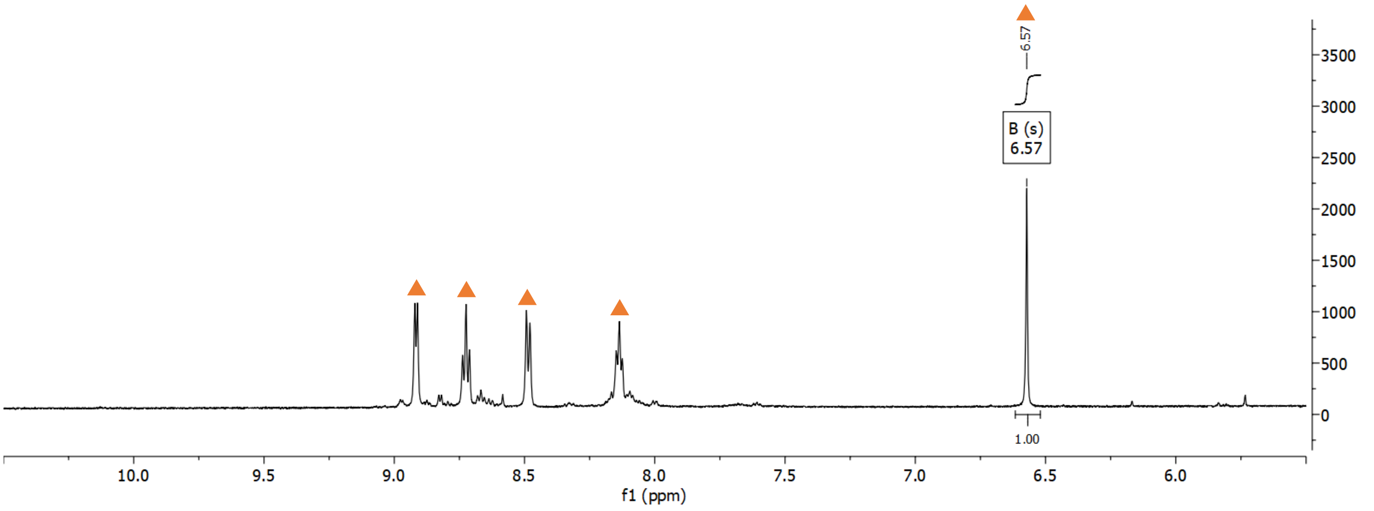

(c) (t = 17 h)

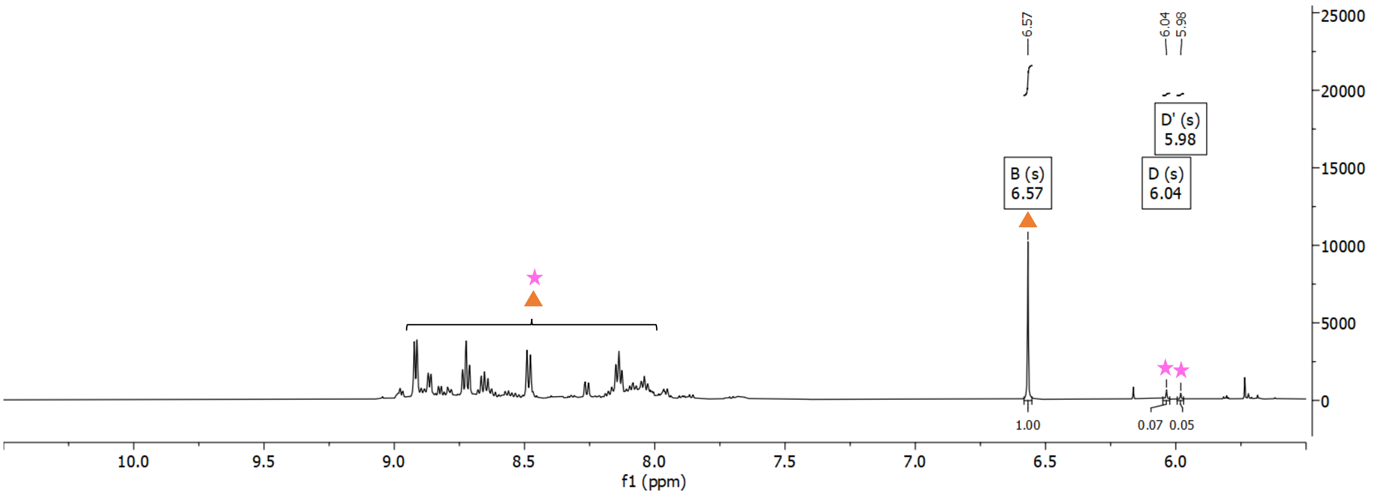

PCA 13

(a)

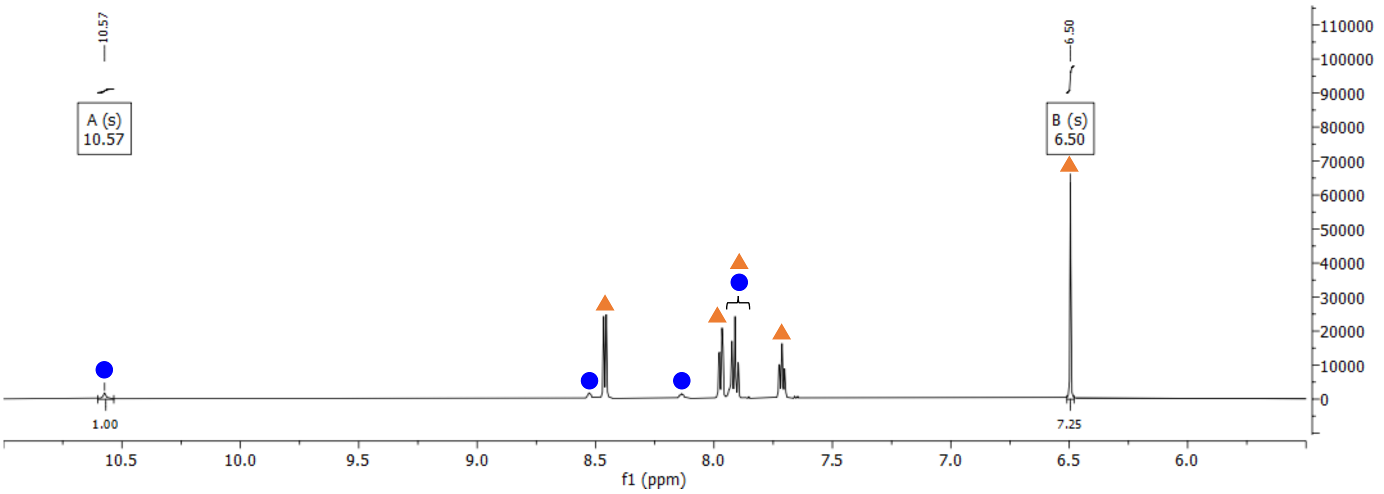

(b) (t = 17 h)

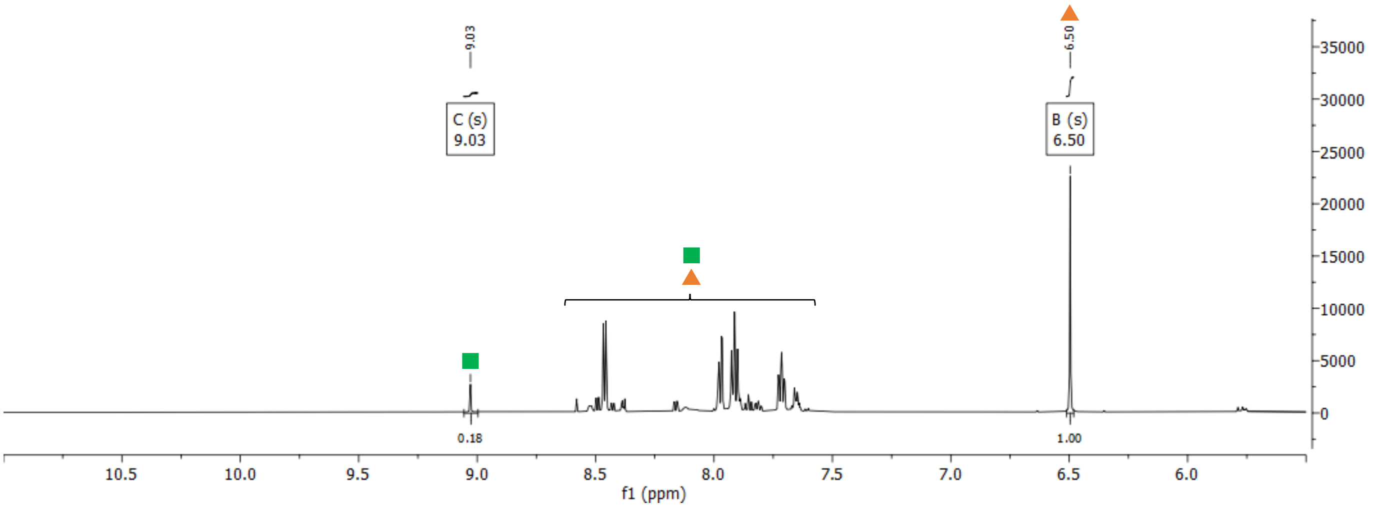

(c) (t = 17 h)

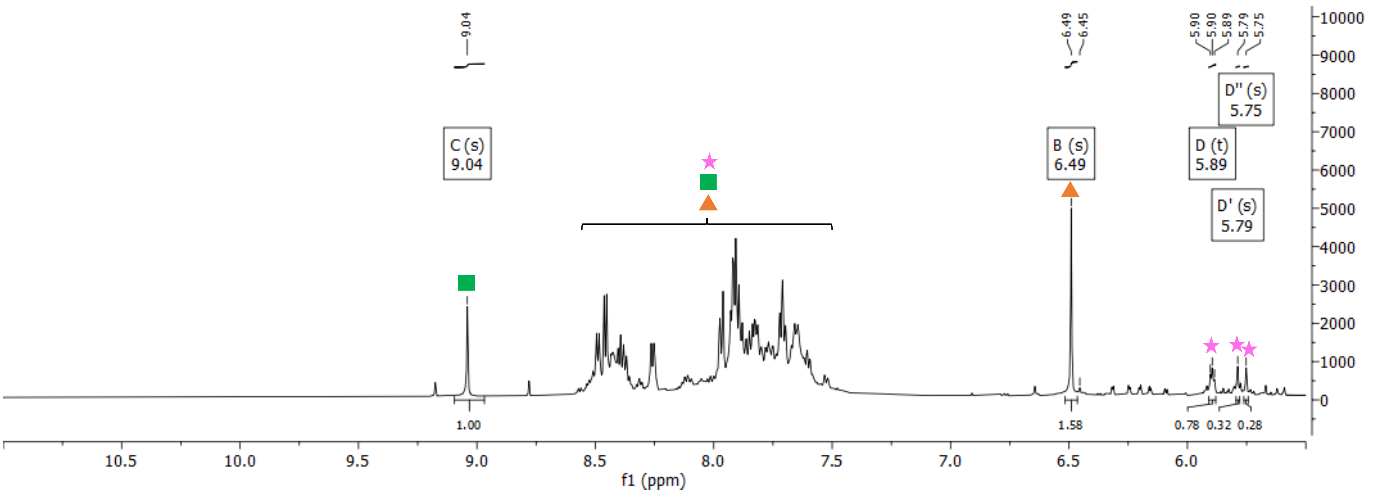

Figure S3. Representative ^{1}H NMR spectra of hydrate equilibria experiments showing the diagnostic signals used to calculate: ***(a)*** the equilibrium constant for hydrate formation; ***(b)*** the equilibrium constant for imine

formation; ***(c)*** rate constants for imidazolidinone formation (**triangle** = hydrate species, **circle** = aldehyde species, **square** = imine species, **star** = imidazolidinone species).

4. pK_a calculation

Hydroxy-PCA analogues **3**, **5**, or **9** were dissolved in water (5 mL) at a concentration of 50 mM. The solution was then acidified by the addition of concentrated hydrochloric acid (5 µL). Aliquots of sodium hydroxide solution (0.1 M, 100 µL) were added and the pH of the solution measured after each addition. The pK_a of the hydroxyl group was calculated by determining the half equivalence point.

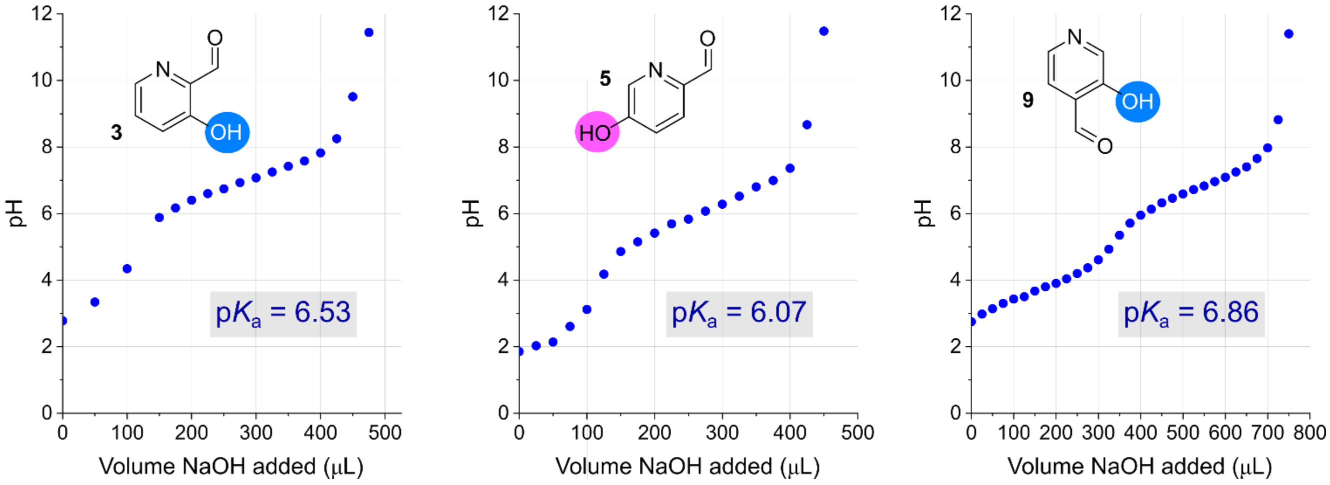

5. Protein Modification

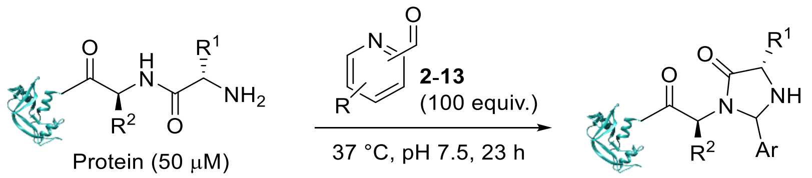

*Conditions for the modification of proteins with PCAs **2-13** were adapted from those reported by MacDonald et al.*[7] A stock solution of PCA **2-13** (85 µL, 10 mM, 850 nmol, 100 equiv., in 50 mM pH 7.5 sodium phosphate buffer) was added to a solution of protein (85 µL, 100 µM, 8.5 nmol, 1 equiv., in 50 mM pH 7.5 sodium phosphate buffer), and the mixture incubated at 37 °C for 23 h with agitation (1000 rpm). Conversion was determined by LC-MS analysis without purification (Crude). Protein conjugates **P** were then purified by dialysis to remove excess reagent (4 °C, 3.5 kDa MWCO; 1 × 50 mM pH 7 sodium phosphate buffer, 2 h; 1 × water, 3 h; 1 × water, 16 h; 1 × water, 4 h), and conversion was again determined by LC-MS analysis (Purified).

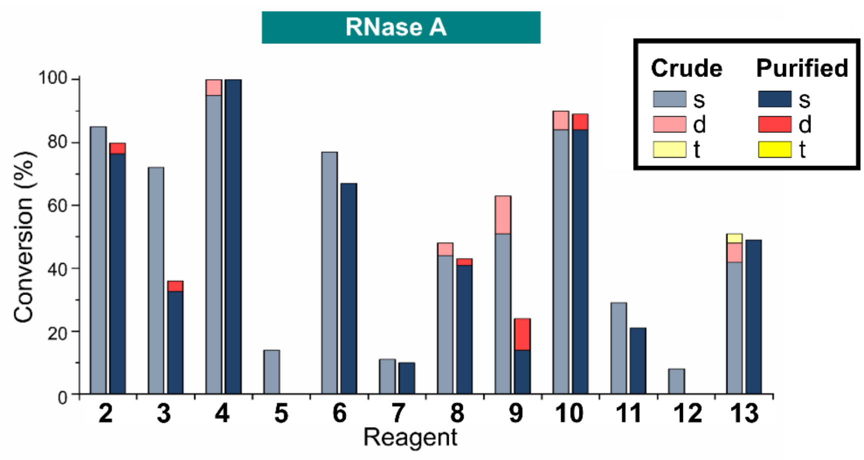

Figure S4. Conversions for the modification of RNase A before (crude) and after (purified) dialysis at 4 °C. s = single, d = double, t = triple modification.

	**PCA**	**Conversion (%)**	
		Crude	*Purified*
**RNase A**	**2**	**85** *(s)*	**80**<br>*76(s):3(d)*
	**3**	**72** *(s)*	**36**<br>*33(s):3(d)*
	**4**	**100**<br>*95(s):5(d)*	**100** *(s)*
	**5**	**14** *(s)*	**0**
	**6**	**77** *(s)*	**67** *(s)*
	**7**	**11** *(s)*	**10** *(s)*
	**8**	**48**<br>*44(s):4(d)*	**43**<br>*41(s):2(d)*
	**9**	**63**<br>*51(s):12(d)*	**24**<br>*14(s):10(d)*
	**10**	**90**<br>*84(s):6(d)*	**89**<br>*84(s):5(d)*
	**11**	**29** *(s)*	**21** *(s)*
	**12**	**8** *(s)*	**0**
	**13**	**51**<br>*42(s):6(d):3(t)*	**49** *(s)*

Table S4. Conversions for the modification of RNase A before (crude) and after (purified) dialysis at 4 °C. s = single, d = double, t = triple modification.

RNase A *(crude)*

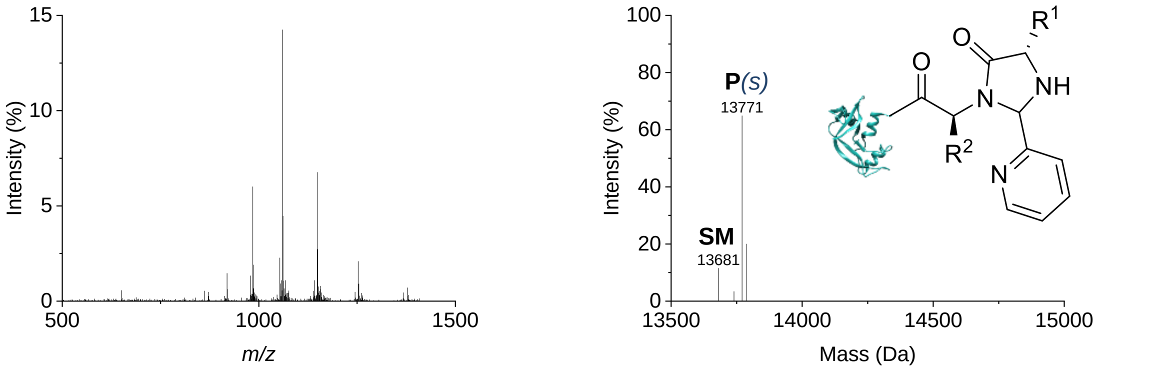

PCA 2

85%

MS (ESI+) [**SM**+H]+ found 13681, calculated 13681; [**SM**+MeCN+NH_4]+ found 13740, calculated 13739; [**P**(s)+H]+ found 13771, calculated 13770; [**P**(s)+H_2O+H]+ found 13786, calculated 13788.

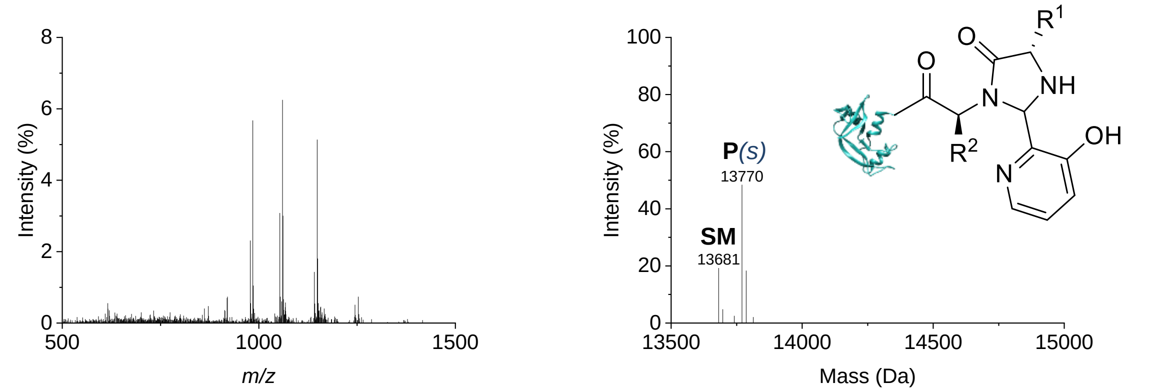

PCA 3

72%

MS (ESI+) [**SM**+H]+ found 13681, calculated 13681; [**SM**+H_2O+H]+ found 13697, calculated 13699; [**SM**+MeCN+NH_4]+ found 13741, calculated 13739; [**P**(s)-H_2O+H]+ found 13770, calculated 13768; [**P**(s)+H]+ found 13786, calculated 13786; [**P**(s)-H_2O+MeCN+H]+ found 13814, calculated 13809.

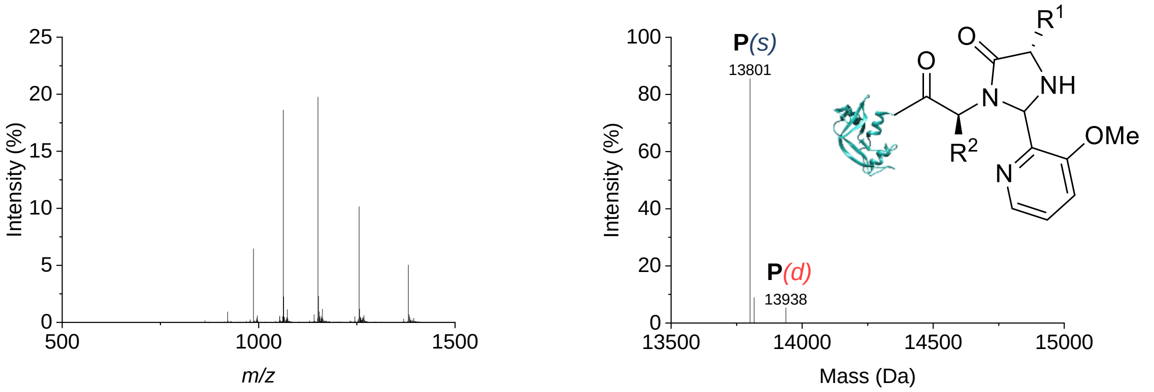

PCA 4

100%

95(s):5(d)

MS (ESI+) [**P**(s)+H]+ found 13801, calculated 13800; [**P**(s)+H_2O+H]+ found 13817, calculated 13818; [**P**(d)+H_2O+H]+ found 13938, calculated 13937.

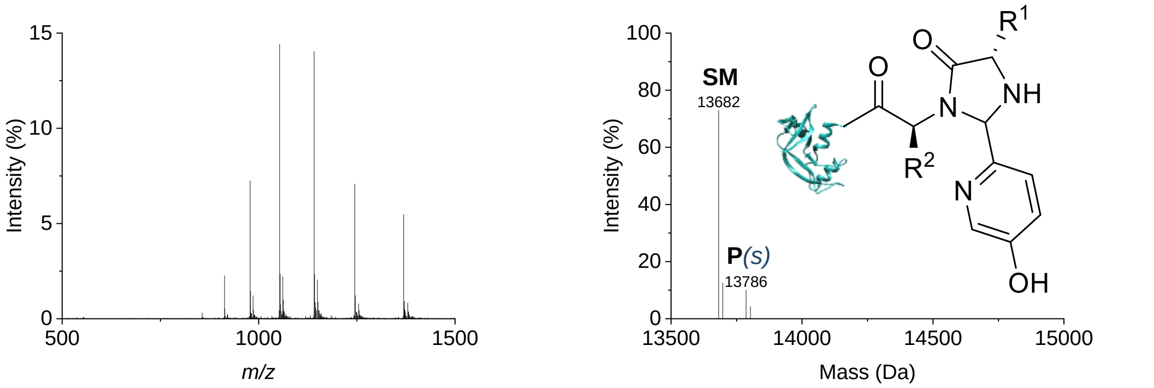

PCA 5

14%

MS (ESI+) [**SM**+H]+ found 13682, calculated 13681; [**SM**+H_2O+H]+ found 13698, calculated 13699; [**P**(s)+H]+ found 13786, calculated 13786; [**P**(s)+H_2O+H]+ found 13803, calculated 13804.

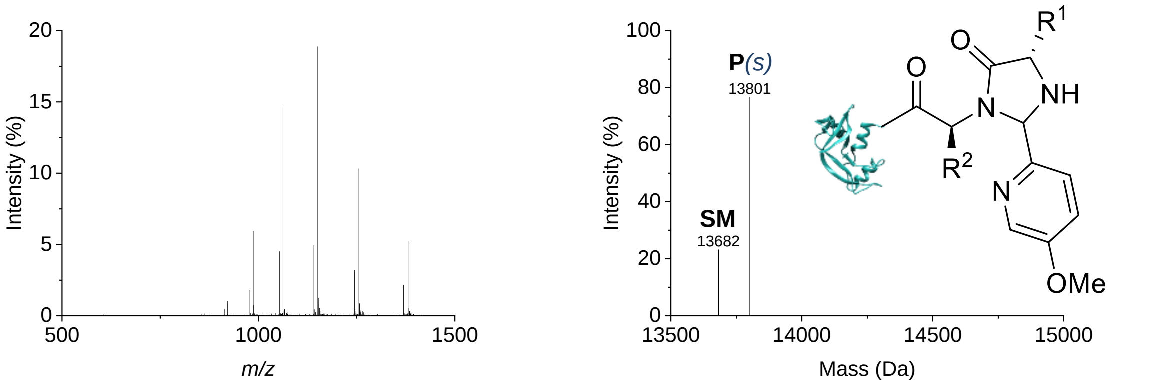

PCA 6

77%

MS (ESI^+) [**SM**+H]$^+$ found 13682, calculated 13681; [**P**(s)+H]$^+$ found 13801, calculated 13800.

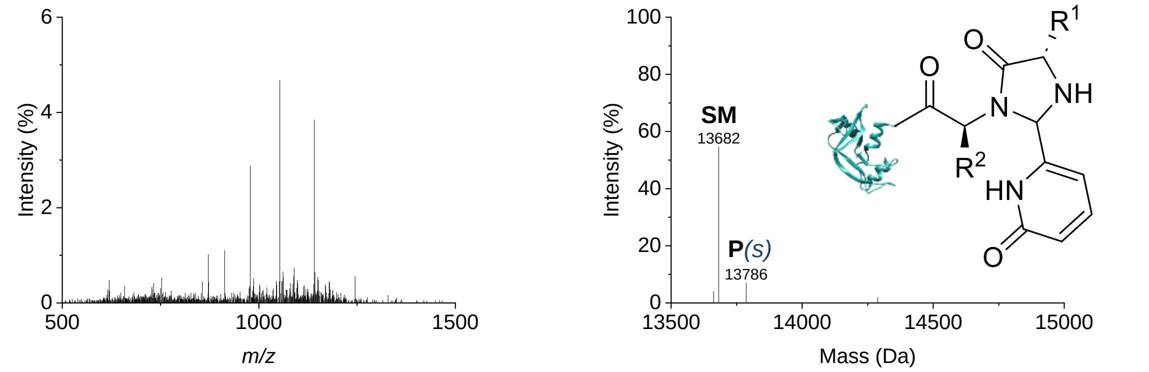

PCA 7

11%

MS (ESI^+) [**SM**-H_2O+H]$^+$ found 13662, calculated 13663; [**SM**+H]$^+$ found 13682, calculated 13681; [**P**(s)+H]$^+$ found 13786, calculated 13786.

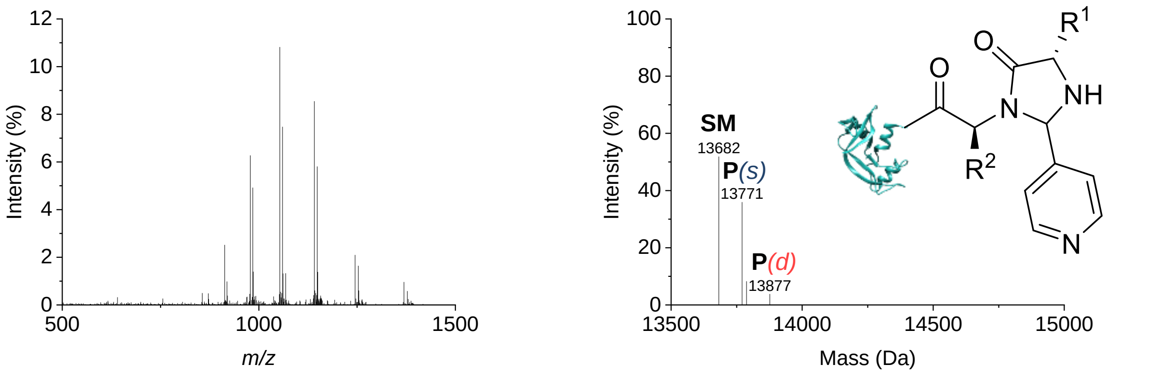

PCA 8

48%

44(s):4(d)

MS (ESI^+) [**SM**+H]$^+$ found 13682, calculated 13681; [**P**(s)+H]$^+$ found 13771, calculated 13770; [**P**(s)+H_2O+H]$^+$ found 13788, calculated 13788; [**P**(d)+H_2O+H]$^+$ found 13877, calculated 13877.

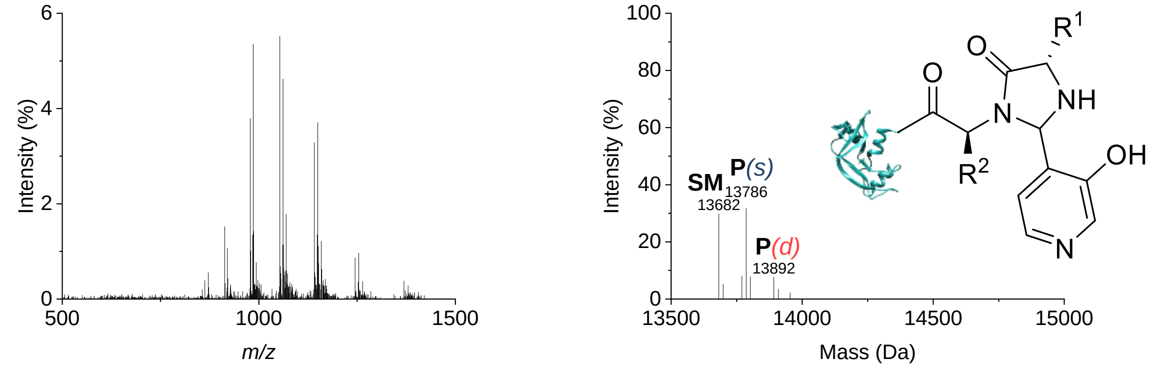

PCA 9

63%

51(s):12(d)

MS (ESI^+) [**SM**+H]$^+$ found 13682, calculated 13681; [**SM**+H_2O+H]$^+$ found 13699, calculated 13699; [**P**(s)-H_2O+H]$^+$ found 13770, calculated 13768; [**P**(s)+H]$^+$ found 13786, calculated 13786; [**P**(s)+H_2O+H]$^+$ found 13802, calculated 13804; [**P**(d)+H]$^+$ found 13892, calculated 13891; [**P**(d)+H_2O+H]$^+$ found 13909, calculated 13909.

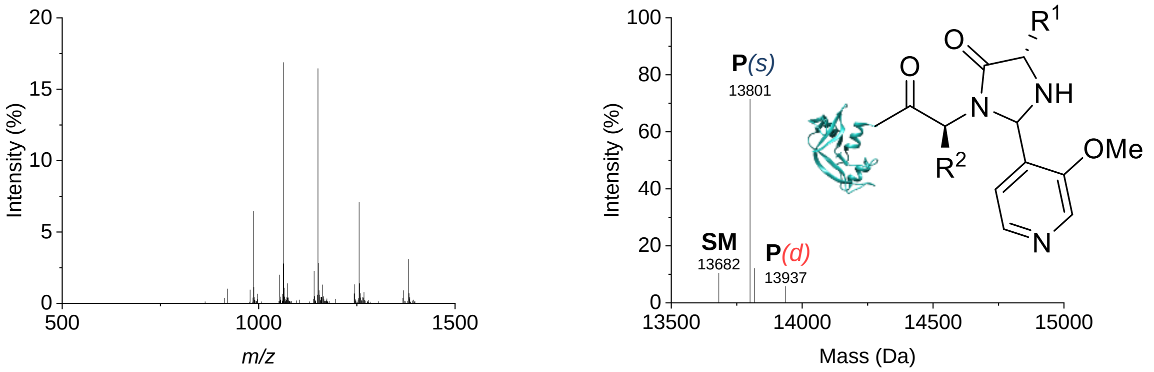

PCA 10

90%

84(s):6(d)

MS (ESI$^+$) [**SM**+H]$^+$ found 13682, calculated 13681; [**P**(s)+H]$^+$ found 13801, calculated 13800; [**P**(s)+H_2O+H]$^+$ found 13817, calculated 13818; [**P**(d)+H]$^+$ found 13937, calculated 13937.

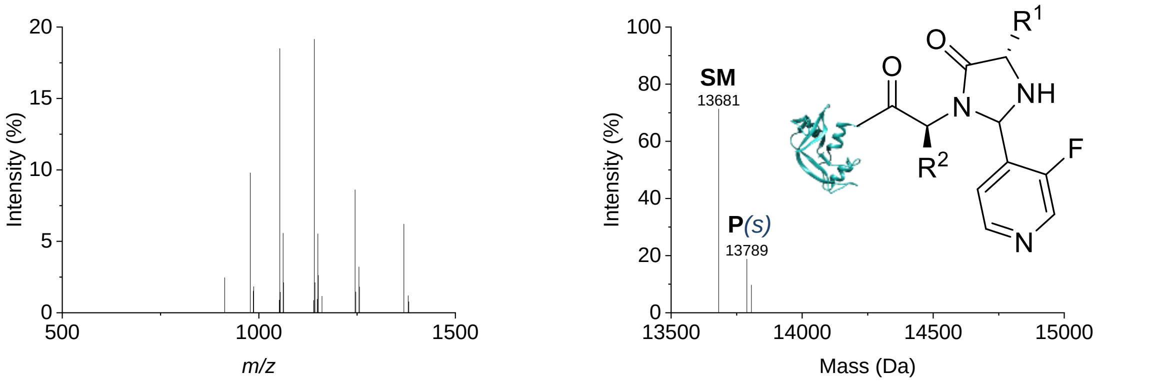

PCA 11

29%

MS (ESI$^+$) [**SM**+H]$^+$ found 13681, calculated 13681; [**P**(s)+H]$^+$ found 13789, calculated 13788; [**P**(s)+H_2O+H]$^+$ found 13806, calculated 13806.

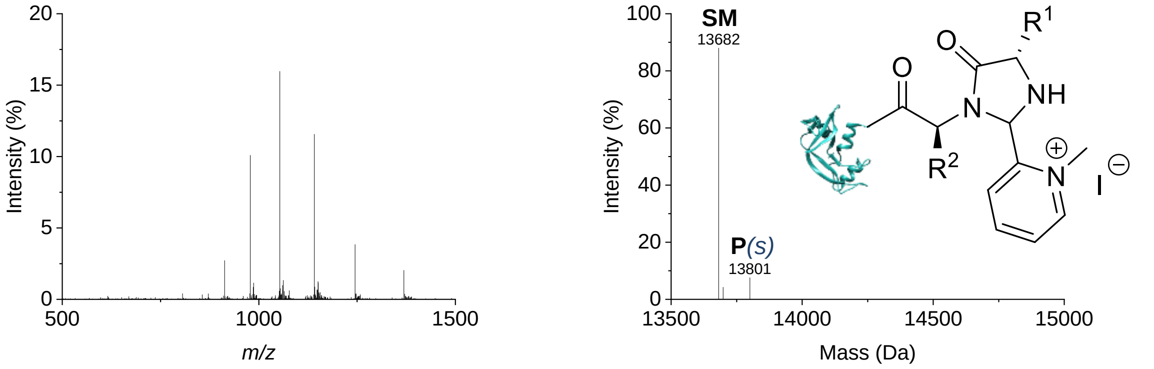

PCA 12

8%

MS (ESI$^+$) [**SM**+H]$^+$ found 13682, calculated 13681; [**SM**+H_2O+H]$^+$ found 13698, calculated 13699; **P**(s)+H_2O+H]$^+$ found 13801, calculated 13803.

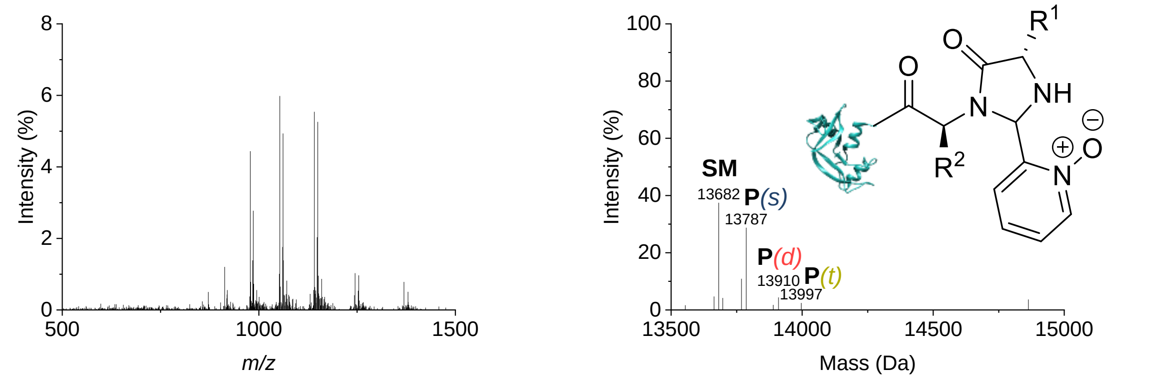

PCA 13

51%

42(s):

6(d):3(t)

MS (ESI$^+$) [**SM**-H_2O+H]$^+$ found 13664, calculated 13663; [**SM**+H]$^+$ found 13682, calculated 13681; [**SM**+H_2O+H]$^+$ found 13697, calculated 13699; [**P**(s)-H_2O+H]$^+$ found 13768, calculated 13768; [**P**(s)+H]$^+$ found 13787, calculated 13786; [**P**(d)+H]$^+$ found 13890, calculated 13891; [**P**(d)+H_2O+H]$^+$ found 13910, calculated 13909; [**P**(t)+H]$^+$ found 13997, calculated 13996.

RNase A *(purified)*

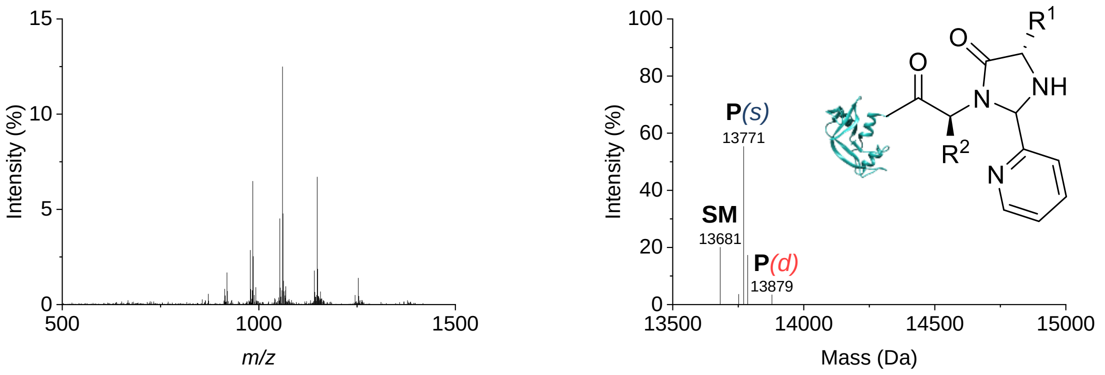

PCA 2

85%

76(s):3(d)

MS (ESI$^+$) [**SM**+H]$^+$ found 13681, calculated 13681; [**P**(s)-H_2O+H]$^+$ found 13752, calculated 13752; [**P**(s)+H]$^+$ found 13771, calculated 13770; [**P**(s)+H_2O+H]$^+$ found 13786, calculated 13788; [**P**(d)+H_2O+H]$^+$ found 13879, calculated 13877.

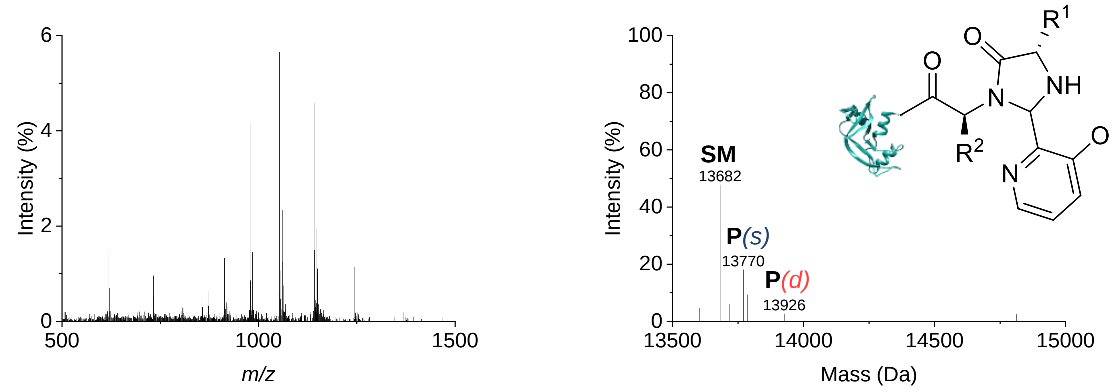

PCA 3

36%

33(s):3(d)

MS (ESI$^+$) [**SM**+H]$^+$ found 13682, calculated 13681; [**SM**+MeOH+H]$^+$ found 13717, calculated 13713; [**P**(s)-H_2O+H]$^+$ found 13770, calculated 13768; [**P**(s)+H]$^+$ found 13787, calculated 13786; [**P**(d)+2H_2O+H]$^+$ found 13926, calculated 13927.

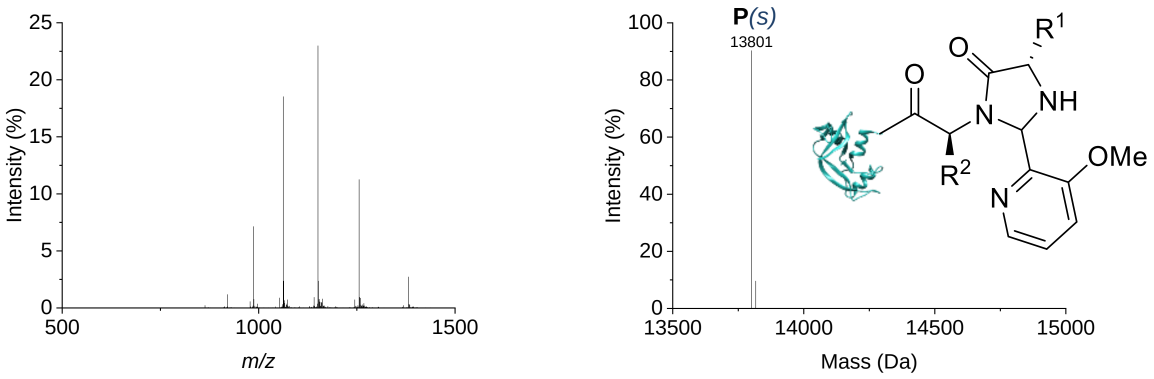

PCA 4

100%

MS (ESI$^+$) [**P**(s)+H]$^+$ found 13801, calculated 13800; [**P**(s)+H_2O+H]$^+$ found 13817, calculated 13818.

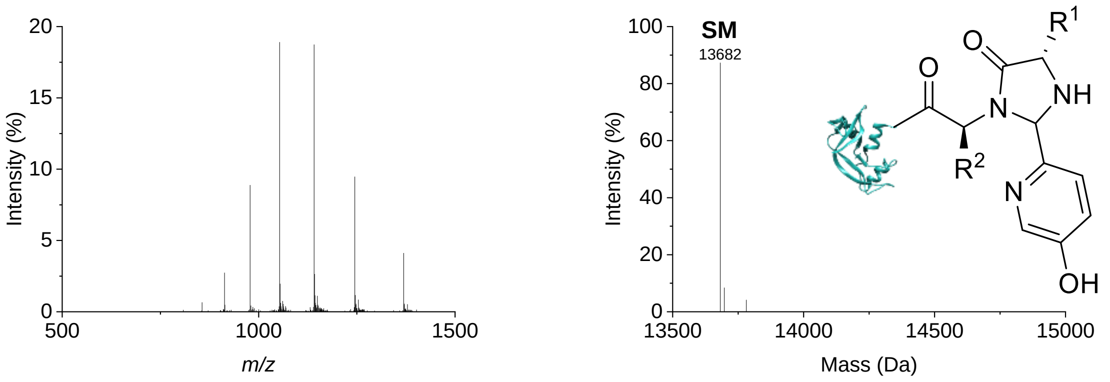

PCA 5

0%

MS (ESI$^+$) [**SM**+H]$^+$ found 13682, calculated 13681; [**SM**+H_2O+H]$^+$ found 13697, calculated 13699; [**SM**+H_3PO_4+H]$^+$ found 13781, calculated 13779.

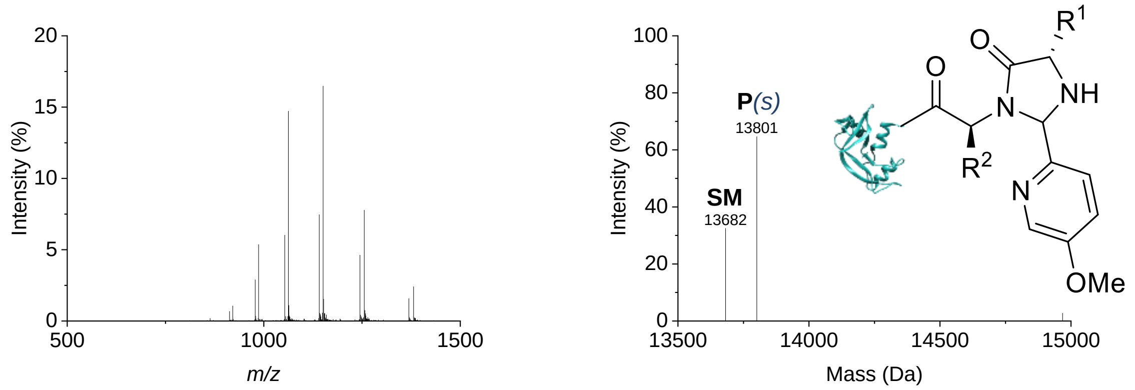

PCA 6

67%

MS (ESI+) [**SM**+H]+ found 13682, calculated 13681; [**P**(s)+H]+ found 13801, calculated 13800.

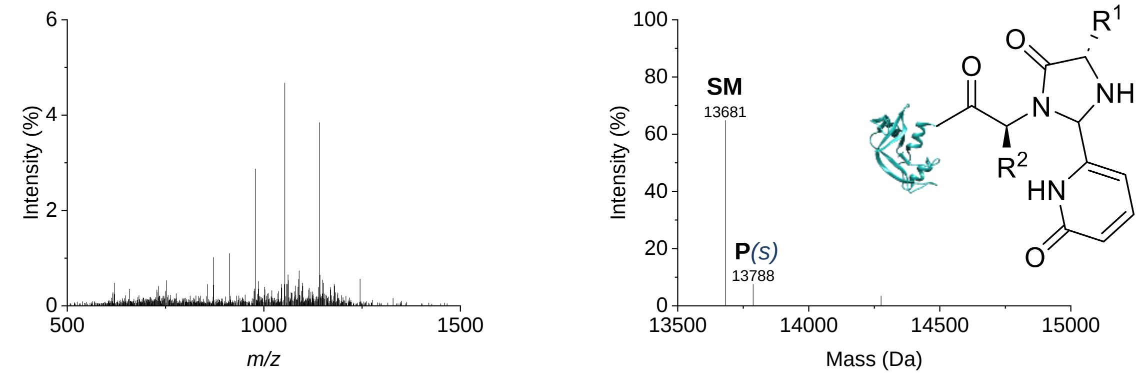

PCA 7

10%

MS (ESI+) [**SM**+H]+ found 13681, calculated 13681; [**P**(s)+H]+ found 13788, calculated 13786.

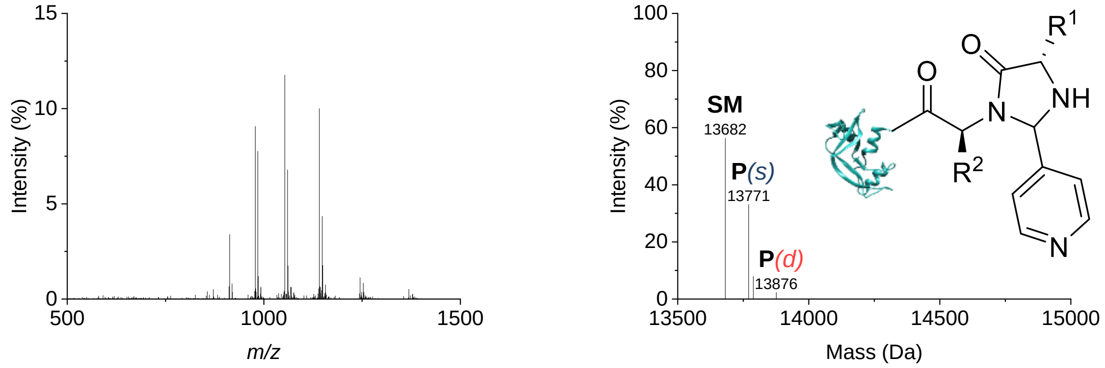

PCA 8

43%

41(s):2(d)

MS (ESI+) [**SM**+H]+ found 13682, calculated 13681; [**P**(s)+H]+ found 13771, calculated 13770; [**P**(s)+H_2O+H]+ found 13788, calculated 13788; [**P**(d)+H_2O+H]+ found 13876, calculated 13877.

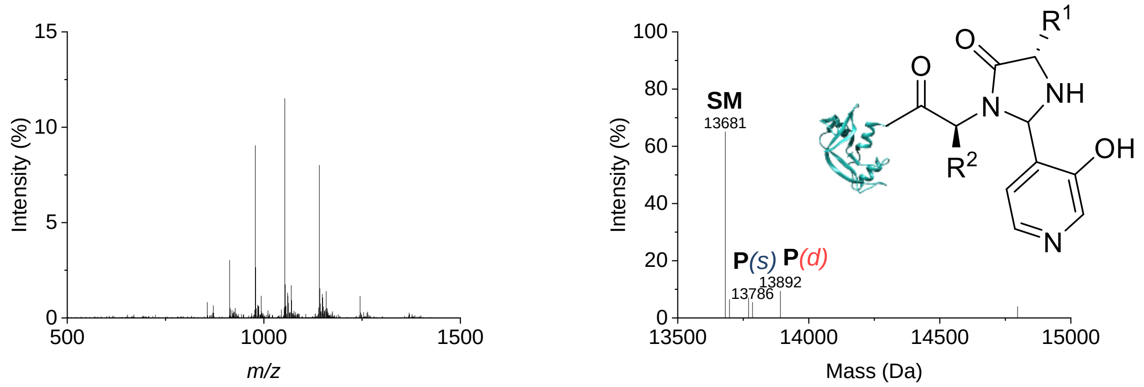

PCA 9

24%

14(s):10(d)

MS (ESI+) [**SM**+H]+ found 13681, calculated 13681; [**SM**+H_2O+H]+ found 13698, calculated 13699; [**P**(s)-H_2O+H]+ found 13770, calculated 13768; [**P**(s)+H]+ found 13786, calculated 13786; [**P**(d)+H]+ found 13892, calculated 13891.

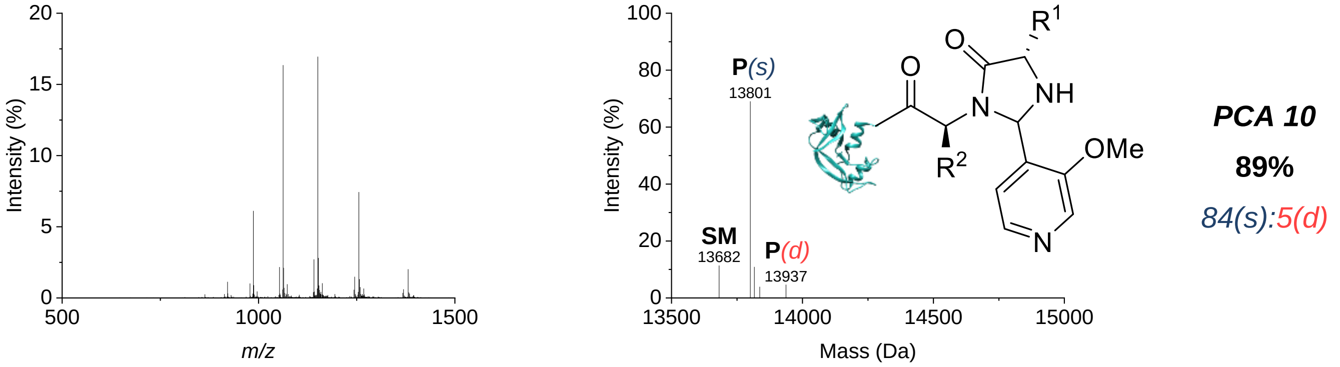

MS (ESI$^+$) [**SM**+H]$^+$ found 13682, calculated 13681; [**P**(s)+H]$^+$ found 13801, calculated 13800; [**P**(s)+H_2O+H]$^+$ found 13816, calculated 13818; [**P**(s)+MeOH+H]$^+$ found 13837, calculated 13832; [**P**(d)+H]$^+$ found 13937, calculated 13937.

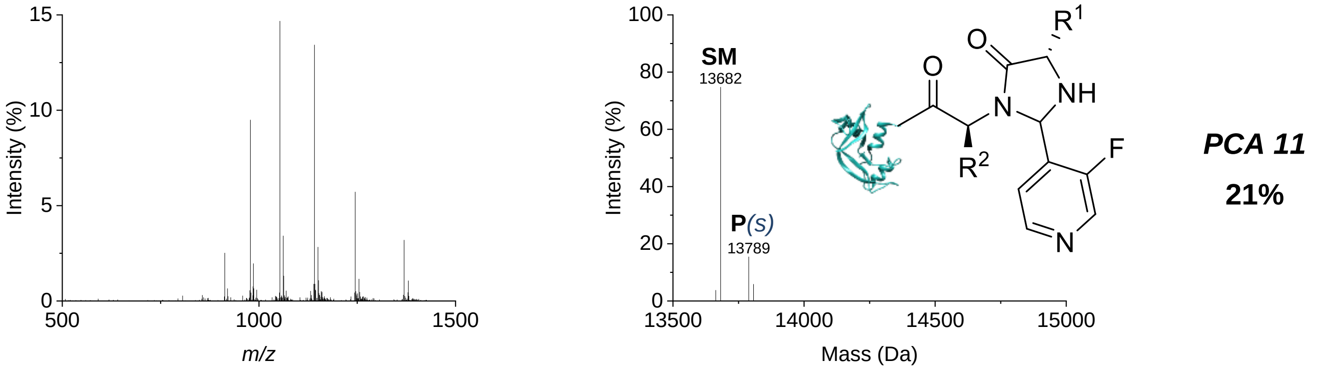

MS (ESI$^+$) [**SM**-H_2O+H]$^+$ found 13663, calculated 13663; [**SM**+H]$^+$ found 13682, calculated 13681; [**P**(s)+H]$^+$ found 13789, calculated 13788; [**P**(s)+H_2O+H]$^+$ found 13807, calculated 13806.

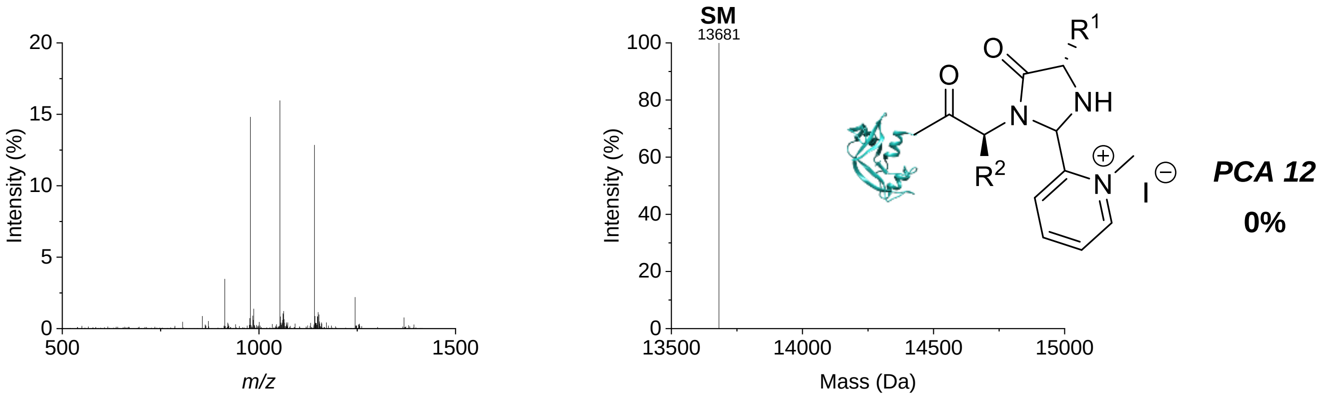

MS (ESI$^+$) [**SM**+H]$^+$ found 13681, calculated 13681.

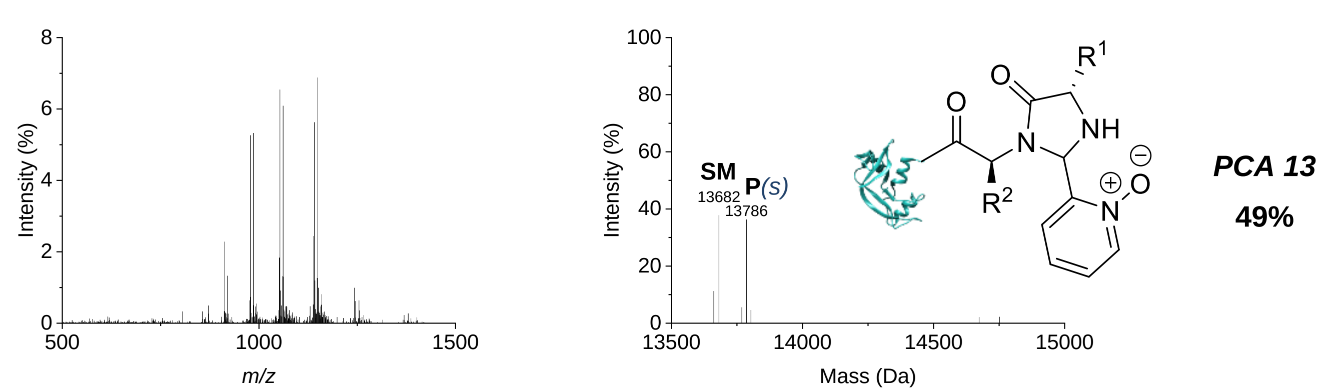

MS (ESI$^+$) [**SM**-H_2O+H]$^+$ found 13662, calculated 13663; [**SM**+H]$^+$ found 13682, calculated 13681; [**P**(s)-H_2O+H]$^+$ found 13769, calculated 13768; [**P**(s)+H]$^+$ found 13786, calculated 13786; [**P**(s)+H_2O+H]$^+$ found 13804, calculated 13804.

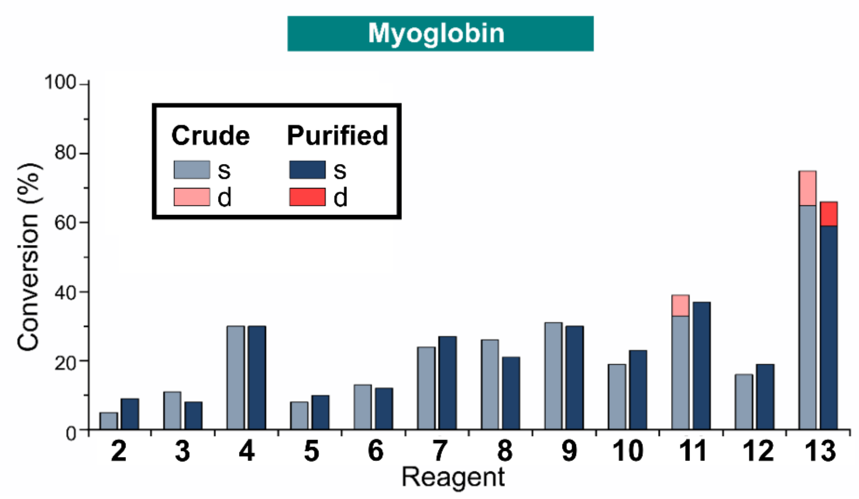

Figure S5. Conversions for the modification of myoglobin before (crude) and after (purified) dialysis at 4 °C. s = single, d = double, t = triple modification.

	PCA	Conversion (%)	
		Crude	*Purified*
Myoglobin	2	**5** *(s)*	**9** *(s)*
	3	**11** *(s)*	**8** *(s)*
	4	**30** *(s)*	**30** *(s)*
	5	**8** *(s)*	**10** *(s)*
	6	**13** *(s)*	**12** *(s)*
	7	**24** *(s)*	**27** *(s)*
	8	**26** *(s)*	**21** *(s)*
	9	**31** *(s)*	**30** *(s)*
	10	**19** *(s)*	**23** *(s)*
	11	**39%** *33(s):6(d)*	**37** *(s)*
	12	**16** *(s)*	**19** *(s)*
	13	**75** *65(s):10(d)*	**66** *59(s):7(d)*

Table S5. Conversions for the modification of myoglobin before (crude) and after (purified) dialysis at 4 °C. s = single, d = double, t = triple modification.

Myoglobin *(crude)*

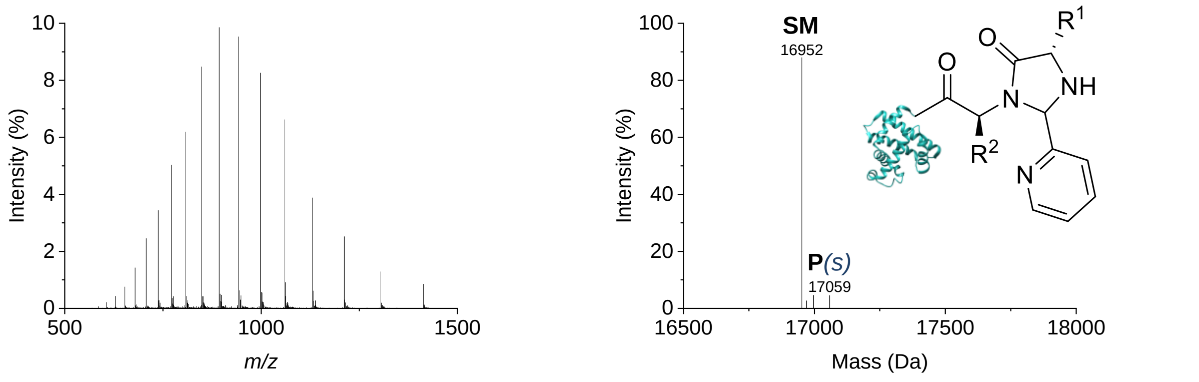

PCA 2

5%

MS (ESI+) [**SM**+H]+ found 16952, calculated 16951; [**SM**+H_2O+H]+ found 16970, calculated 16969; [**SM**+MeCN+H]+ found 16997, calculated 16992; [**P**(s)+H_2O+H]+ found 17059, calculated 17058.

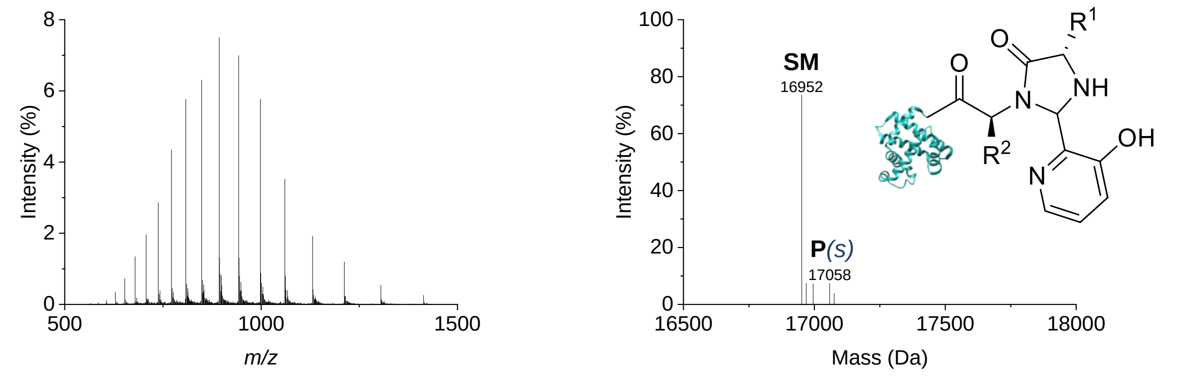

PCA 3

11%

MS (ESI+) [**SM**+H]+ found 16952, calculated 16951; [**SM**+H_2O+H]+ found 16969, calculated 16969; [**SM**+MeCN+H]+ found 16995, calculated 16992; [**P**(s)+H]+ found 17058, calculated 17056; [**P**(s)+H_2O+H]+ found 17076, calculated 17074.

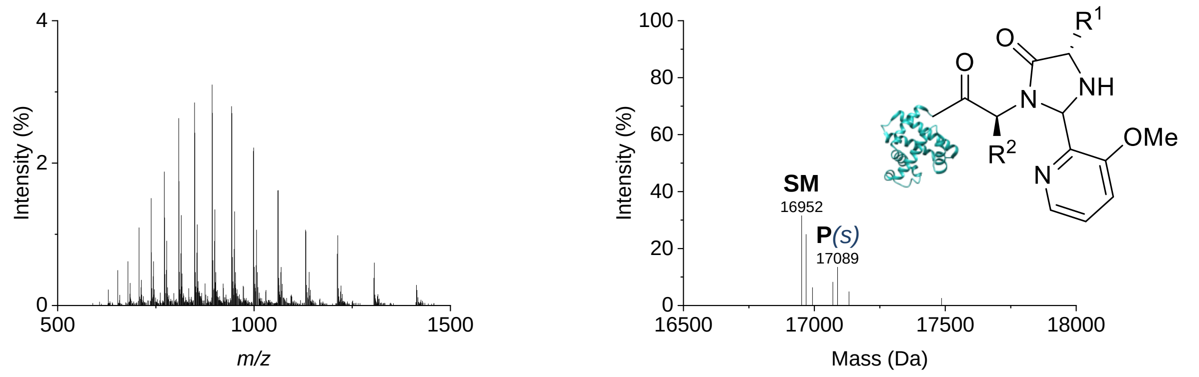

PCA 4

30%

MS (ESI+) [**SM**+H]+ found 16952, calculated 16951; [**SM**+H_2O+H]+ found 16968, calculated 16969; [**SM**+MeCN+H]+ found 16993, calculated 16992; [**P**(s)+H]+ found 17071, calculated 17070; [**P**(s)+H_2O+H]+ found 17089, calculated 17088; [**P**(s)+MeCN+NH_4]+ found 17132, calculated 17128.

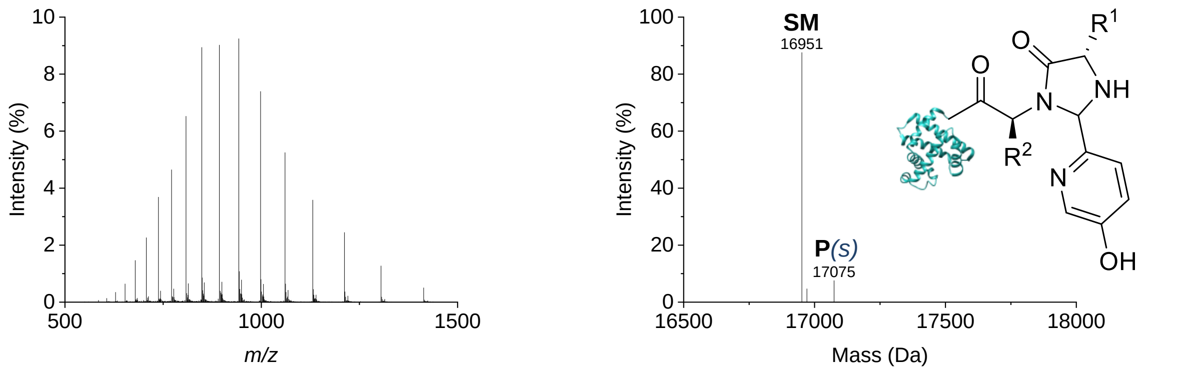

PCA 5

8%

MS (ESI$^+$) [**SM**+H]$^+$ found 16951, calculated 16951; [**SM**+H_2O+H]$^+$ found 16971, calculated 16969; [**P**(s)+H_2O+H]$^+$ found 17075, calculated 17074.

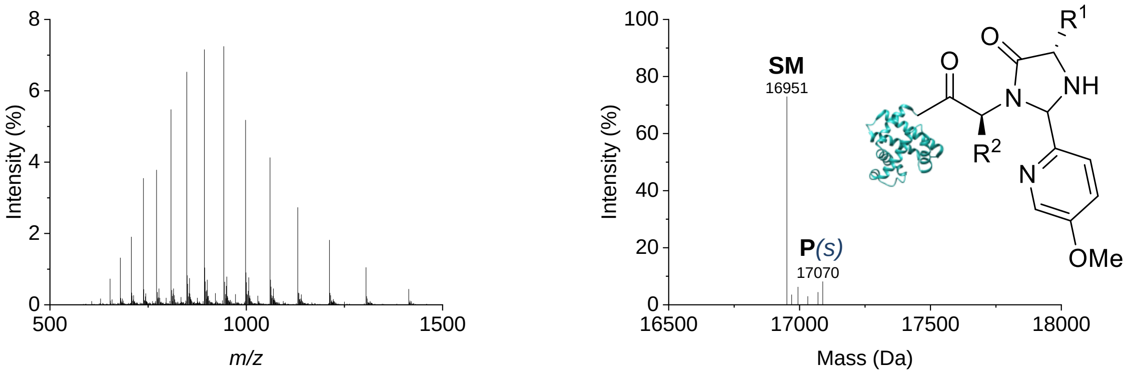

PCA 6

13%

MS (ESI$^+$) [**SM**+H]$^+$ found 16951, calculated 16951; [**SM**+H_2O+H]$^+$ found 16970, calculated 16969; [**SM**+MeCN+H]$^+$ found 16994, calculated 16992; [**SM**+2MeCN+H]$^+$ found 17032, calculated 17033; [**P**(s)+H]$^+$ found 17070, calculated 17070; [**P**(s)+H_2O+H]$^+$ found 17089, calculated 17088.

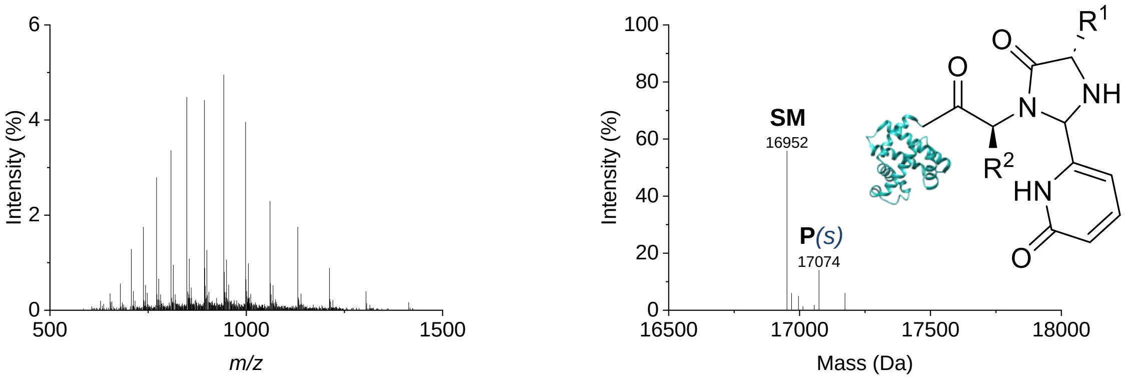

PCA 7

24%

MS (ESI$^+$) [**SM**+H]$^+$ found 16952, calculated 16951; [**SM**+H_2O+H]$^+$ found 16969, calculated 16969; [**SM**+MeCN+H]$^+$ found 16996, calculated 16992; [**SM**+MeCN+Na]$^+$ found 17013, calculated 17014; [**P**(s)+H]$^+$ found 17056, calculated 17056; [**P**(s)+H_2O+H]$^+$ found 17074, calculated 17074; [**P**(s)+H_2O+H_3PO_4+H]$^+$ found 17174, calculated 17172.

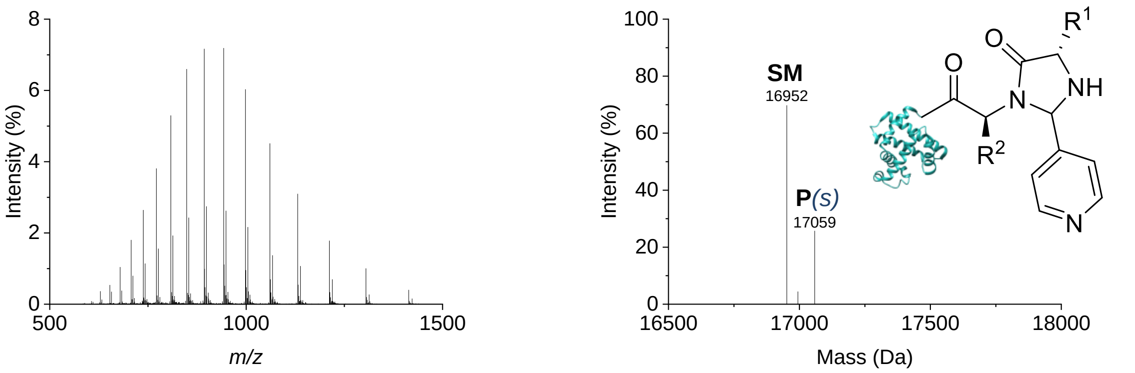

PCA 8

26%

MS (ESI$^+$) [**SM**+H]$^+$ found 16952, calculated 16951; [**SM**+MeCN+H]$^+$ found 16994, calculated 16992; [**P**(s)+H_2O+H]$^+$ found 17059, calculated 17058.

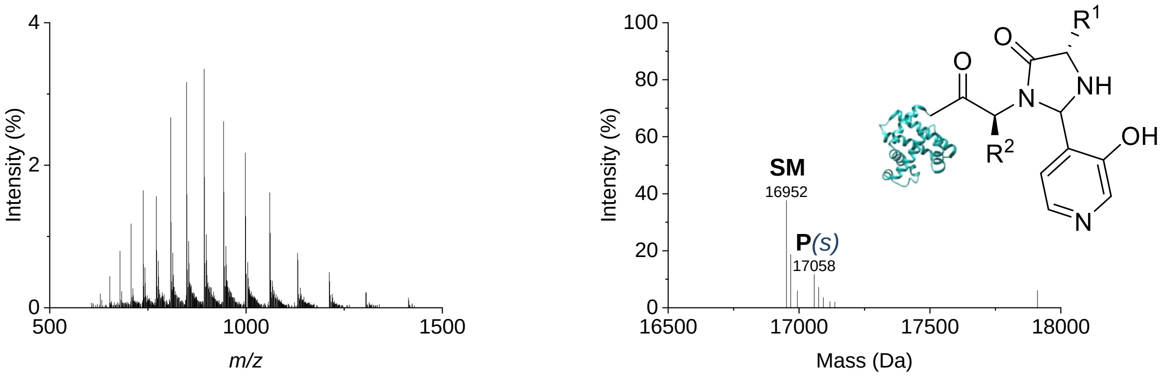

PCA 9

31%

MS (ESI^+) [**SM**+H]$^+$ found 16952, calculated 16951; [**SM**+H_2O+H]$^+$ found 16968, calculated 16969; [**SM**+MeCN+H]$^+$ found 16994, calculated 16992; [**P**(s)+H]$^+$ found 17059, calculated 17056; [**P**(s)+H_2O+H]$^+$ found 17074, calculated 17074; [**P**(s)+MeCN+Na]$^+$ found 17120, calculated 17119; [**P**(s)+DMSO+H]$^+$ found 17137, calculated 17134.

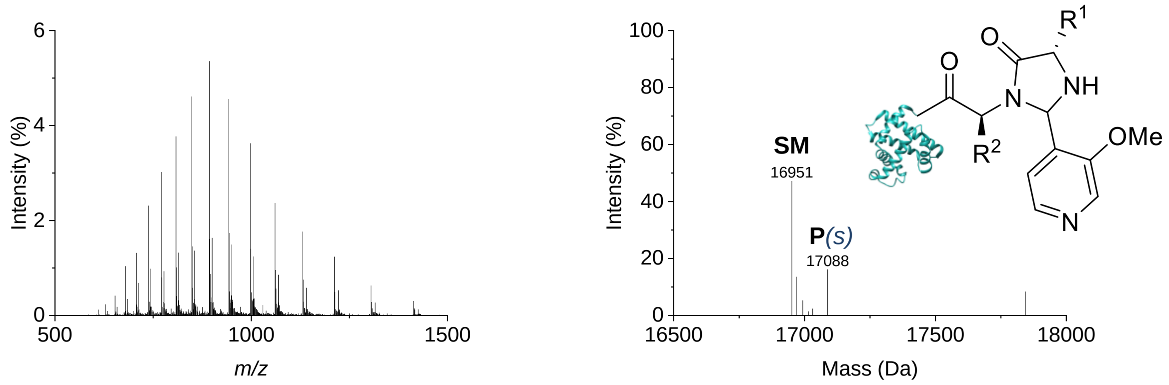

PCA 10

19%

MS (ESI^+) [**SM**+H]$^+$ found 16951, calculated 16951; [**SM**+H_2O+H]$^+$ found 16969, calculated 16969; [**SM**+MeCN+H]$^+$ found 16993, calculated 16992; [**SM**+MeCN+Na]$^+$ found 17015, calculated 17014; [**SM**+2MeCN+H]$^+$ found 17031, calculated 17033; [**P**(s)+H_2O+H]$^+$ found 17088, calculated 17088.

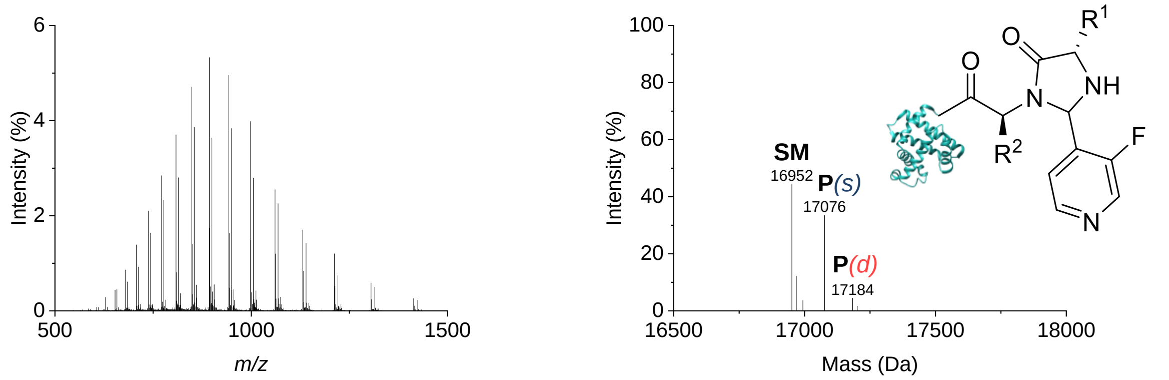

PCA 11

39%

33(s):6(d)

MS (ESI^+) [**SM**+H]$^+$ found 16952, calculated 16951; [**SM**+H_2O+H]$^+$ found 16968, calculated 16969; [**SM**+MeCN+H]$^+$ found 16993, calculated 16992; [**P**(s)+H_2O+H]$^+$ found 17076, calculated 17076; [**P**(d)+H_2O+H]$^+$ found 17184, calculated 17183; [**P**(d)+2H_2O+H]$^+$ found 17201, calculated 17201.

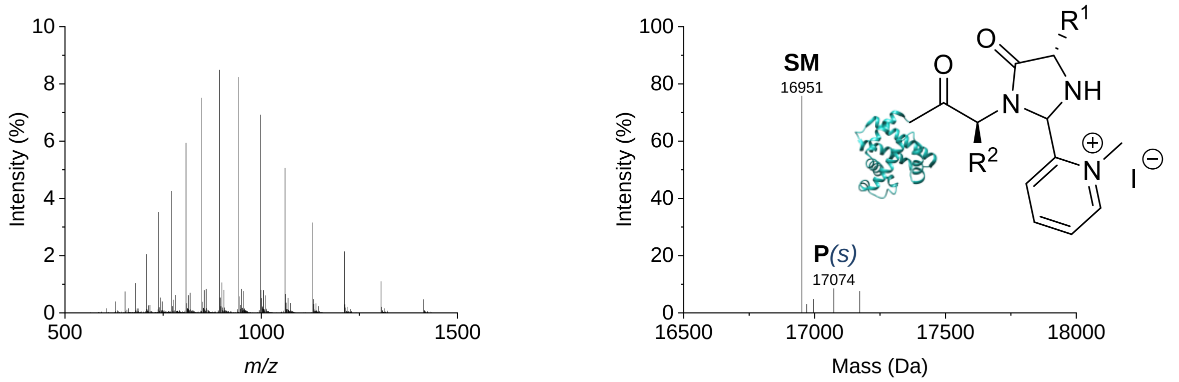

PCA 12

16%

MS (ESI^+) [**SM**+H]$^+$ found 16951, calculated 16951; [**SM**+H_2O+H]$^+$ found 16970, calculated 16969; [**SM**+MeCN+H]$^+$ found 16996, calculated 16992; [**P**(s)+H_2O+H]$^+$ found 17074, calculated 17073; [**P**(s)+H_2O+H_3PO_4+H]$^+$ found 17173, calculated 17171.

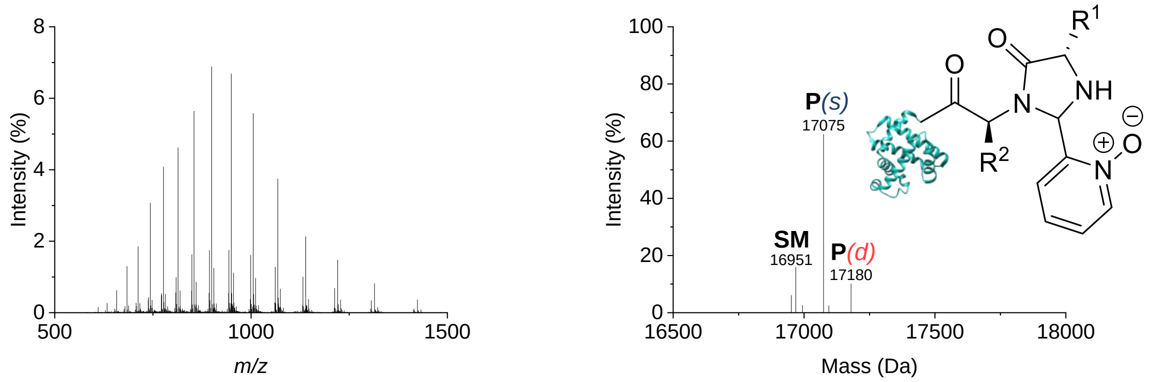

PCA 13

75%

65(s):10(d)

MS (ESI$^+$) [**SM**+H]$^+$ found 16951, calculated 16951; [**SM**+H_2O+H]$^+$ found 16968, calculated 16969; [**SM**+MeCN+H]$^+$ found 16994, calculated 16992; [**P**(s)+H_2O+H]$^+$ found 17075, calculated 17074; [**P**(s)+MeCN+H]$^+$ found 17095, calculated 17097; [**P**(d)+H_2O+H]$^+$ found 17180, calculated 17179.

Myoglobin *(purified)*

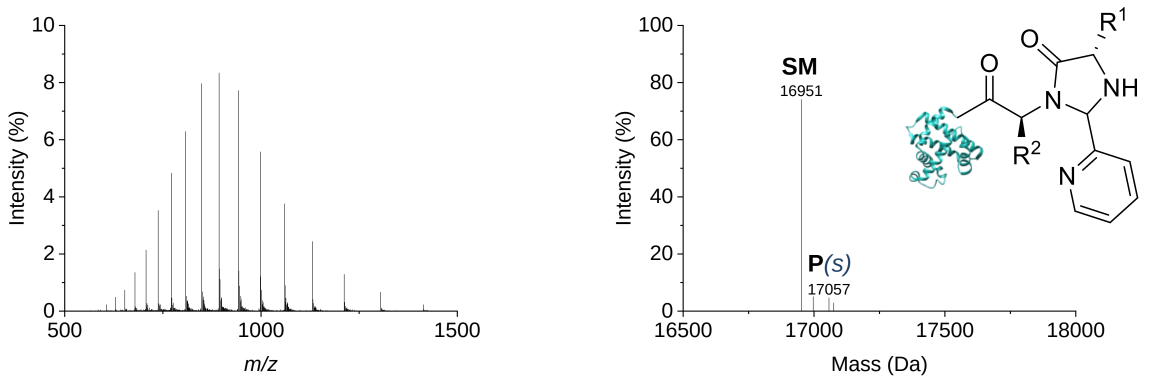

PCA 2

9%

MS (ESI$^+$) [**SM**+H]$^+$ found 16951, calculated 16951; [**SM**+MeCN+H]$^+$ found 16997, calculated 16992; [**P**(s)+H_2O+H]$^+$ found 17057, calculated 17058; [**P**(s)+2H_2O+H]$^+$ found 17076, calculated 17076.

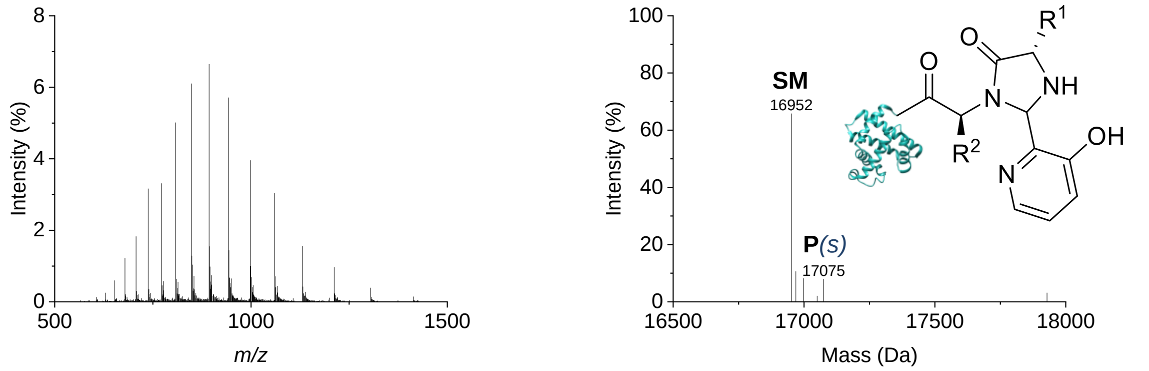

PCA 3

8%

MS (ESI$^+$) [**SM**+H]$^+$ found 16952, calculated 16951; [**SM**+H_2O+H]$^+$ found 16969, calculated 16969; [**SM**+MeCN+H]$^+$ found 16997, calculated 16992; [**SM**+H_3PO_4+H]$^+$ found 17050, calculated 17049; [**P**(s)+H_2O+H]$^+$ found 17075, calculated 17074.

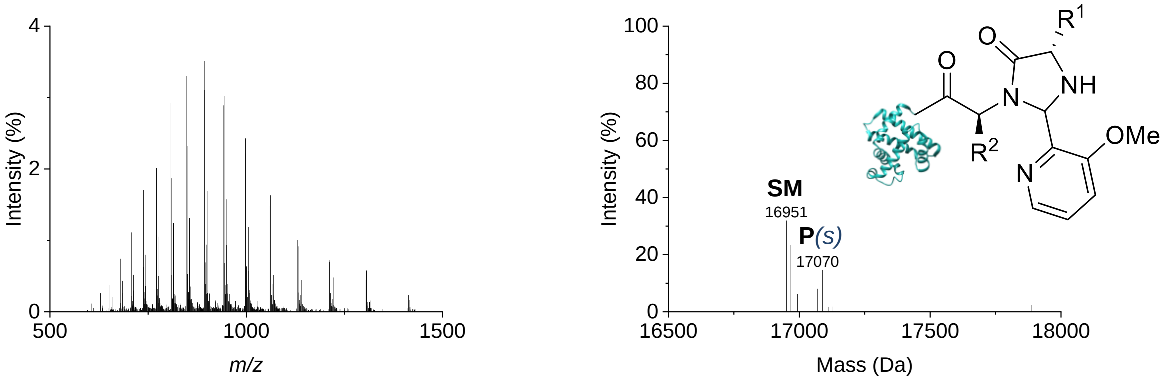

PCA 4

30%

MS (ESI$^+$) [**SM**+H]$^+$ found 16951, calculated 16951; [**SM**+H_2O+H]$^+$ found 16968, calculated 16969; [**SM**+MeCN+H]$^+$ found 16993, calculated 16992; [**P**(s)+H]$^+$ found 17070, calculated 17070; [**P**(s)+H_2O+H]$^+$ found 17088, calculated 17088; [**P**(s)+MeCN+H]$^+$ found 17111, calculated 17111; [**P**(s)+MeCN+NH_4]$^+$ found 17129, calculated 17128.

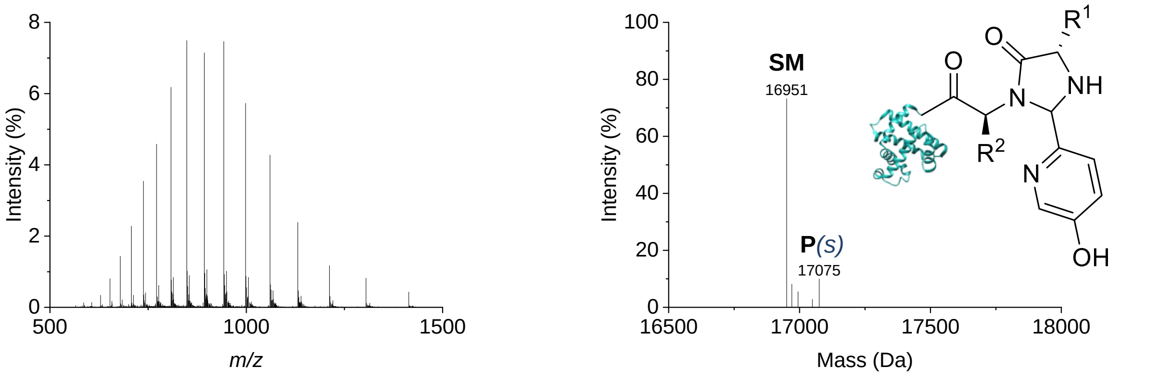

PCA 5

10%

MS (ESI$^+$) [**SM**+H]$^+$ found 16951, calculated 16951; [**SM**+H_2O+H]$^+$ found 16971, calculated 16969; [**SM**+MeCN+H]$^+$ found 16994, calculated 16992; [**SM**+H_3PO_4+H]$^+$ found 17049, calculated 17049; [**P**(s)+H_2O+H]$^+$ found 17075, calculated 17074.

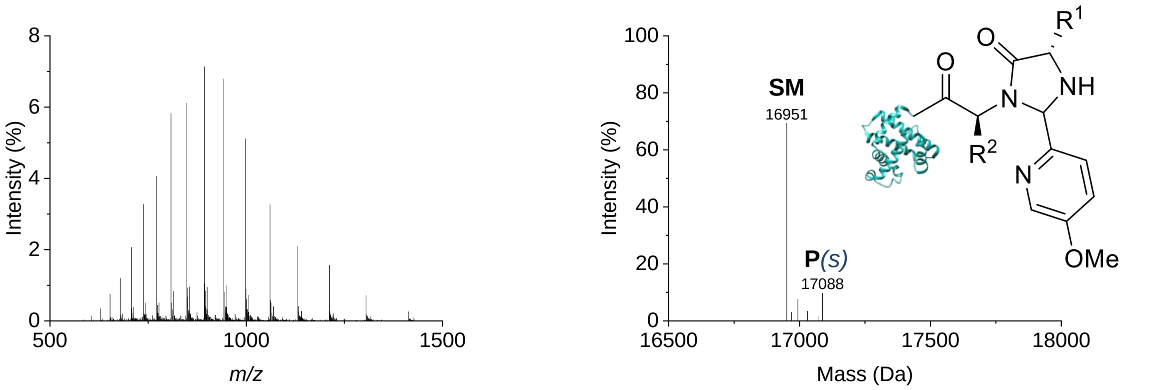

PCA 6

12%

MS (ESI$^+$) [**SM**+H]$^+$ found 16951, calculated 16951; [**SM**+H_2O+H]$^+$ found 16969, calculated 16969; [**SM**+MeCN+H]$^+$ found 16993, calculated 16992; [**SM**+2MeCN+H]$^+$ found 17031, calculated 17033; [**P**(s)+H]$^+$ found 17071, calculated 17070; [**P**(s)+H_2O+H]$^+$ found 17088, calculated 17088.

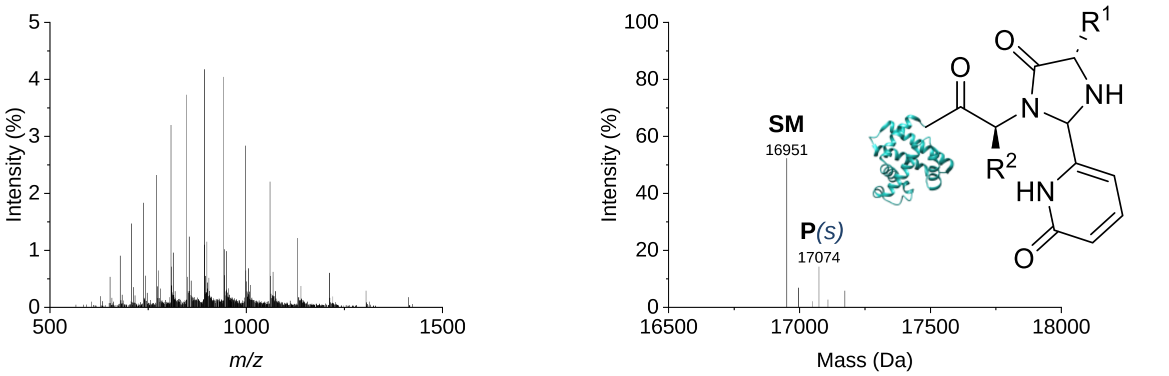

PCA 7

27%

MS (ESI$^+$) [**SM**+H]$^+$ found 16951, calculated 16951; [**SM**+MeCN+H]$^+$ found 16996, calculated 16992; [**SM**+H_3PO_4+H]$^+$ found 17048, calculated 17049; [**P**(s)+H_2O+H]$^+$ found 17074, calculated 17074; [**P**(s)+H_2O+MeOH+H]$^+$ found 17109, calculated 17106; [**P**(s)+H_2O+H_3PO_4+H]$^+$ found 17173, calculated 17172.

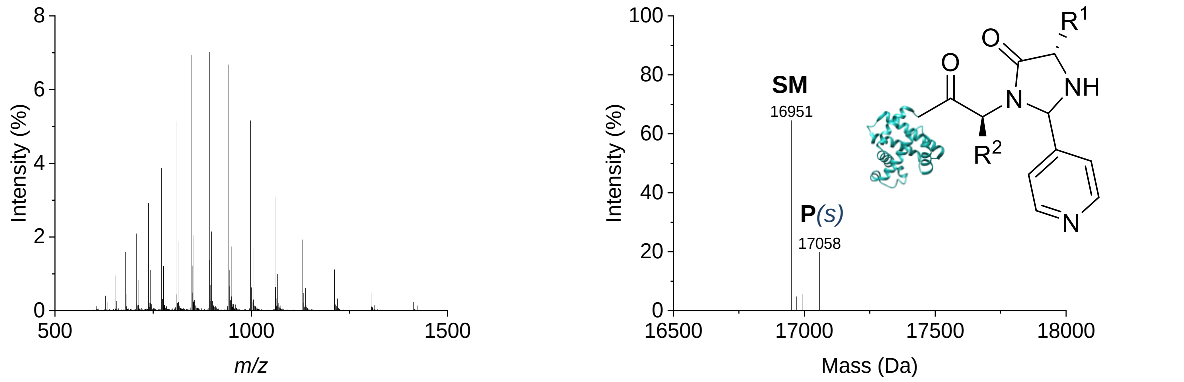

PCA 8

21%

MS (ESI$^+$) [**SM**+H]$^+$ found 16951, calculated 16951; [**SM**+H_2O+H]$^+$ found 16970, calculated 16969; [**SM**+MeCN+H]$^+$ found 16995, calculated 16992; [**P**(s)+H_2O+H]$^+$ found 17058, calculated 17058.

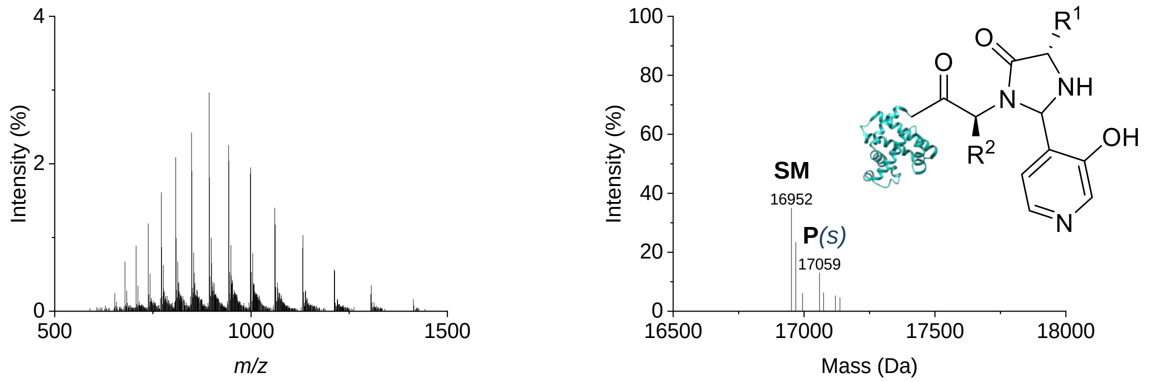

PCA 9

30%

MS (ESI$^+$) [**SM**+H]$^+$ found 16952, calculated 16951; [**SM**+H_2O+H]$^+$ found 16968, calculated 16969; [**SM**+MeCN+H]$^+$ found 16994, calculated 16992; [**P**(s)+H]$^+$ found 17058, calculated 17056; [**P**(s)+H_2O+H]$^+$ found 17075, calculated 17074; [**P**(s)+MeOH+H]$^+$ found 17093, calculated 17088; [**P**(s)+MeCN+Na]$^+$ found 17118, calculated 17119; [**P**(s)+DMSO+H]$^+$ found 17137, calculated 17134.

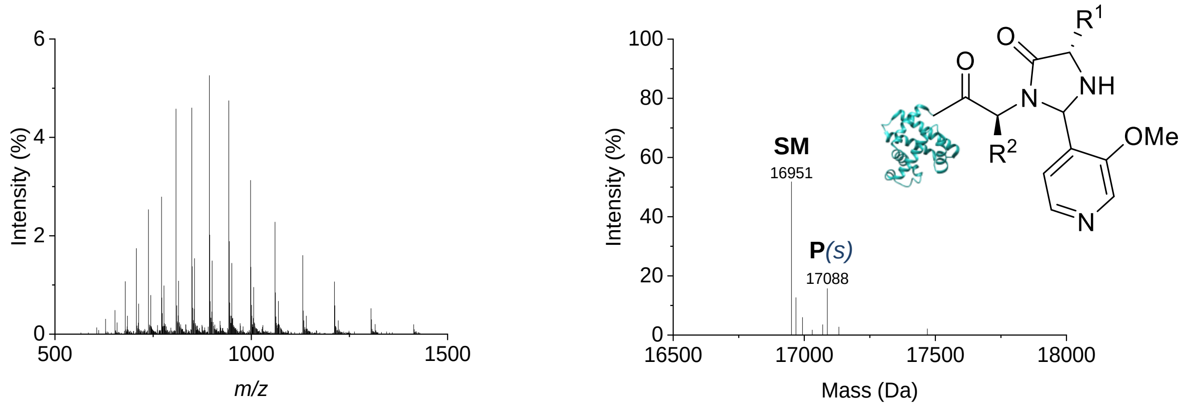

PCA 10

23%

MS (ESI$^+$) [**SM**+H]$^+$ found 16951, calculated 16951; [**SM**+H_2O+H]$^+$ found 16968, calculated 16969; [**SM**+MeCN+H]$^+$ found 16993, calculated 16992; [**SM**+2MeCN+H]$^+$ found 17030, calculated 17033; [**P**(s)+H]$^+$ found 17070, calculated 17070; [**P**(s)+H_2O+H]$^+$ found 17088, calculated 17088; [**P**(s)+MeCN+Na]$^+$ found 17132, calculated 17133.

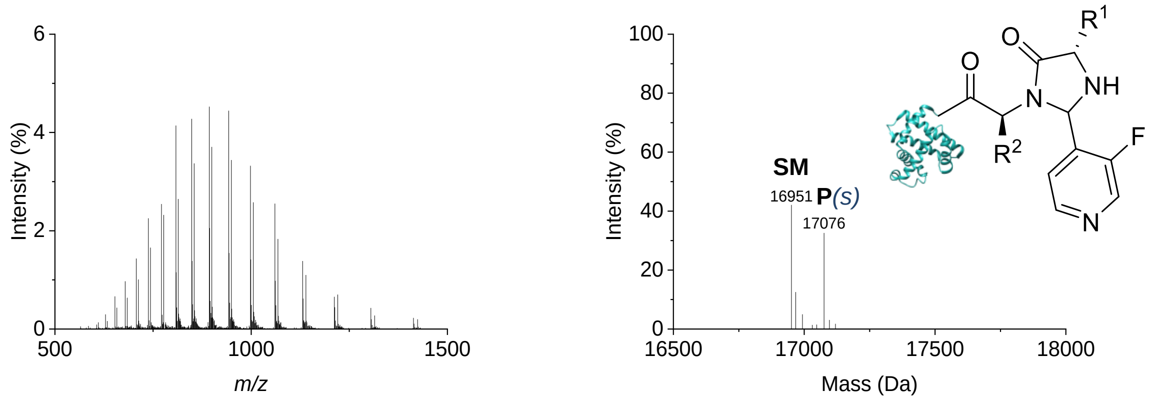

PCA 11

37%

MS (ESI$^+$) [**SM**+H]$^+$ found 16951, calculated 16951; [**SM**+H_2O+H]$^+$ found 16968, calculated 16969; [**SM**+MeCN+H]$^+$ found 16993, calculated 16992; [**SM**+DMSO+H]$^+$ found 17031, calculated 17029; [**SM**+H_3PO_4+H]$^+$ found 17048, calculated 17049; [**P**(s)+H_2O+H]$^+$ found 17076, calculated 17076; [**P**(s)+MeCN+H]$^+$ found 17096, calculated 17099; [**P**(s)+MeCN+H_2O+H]$^+$ found 17120, calculated 17117.

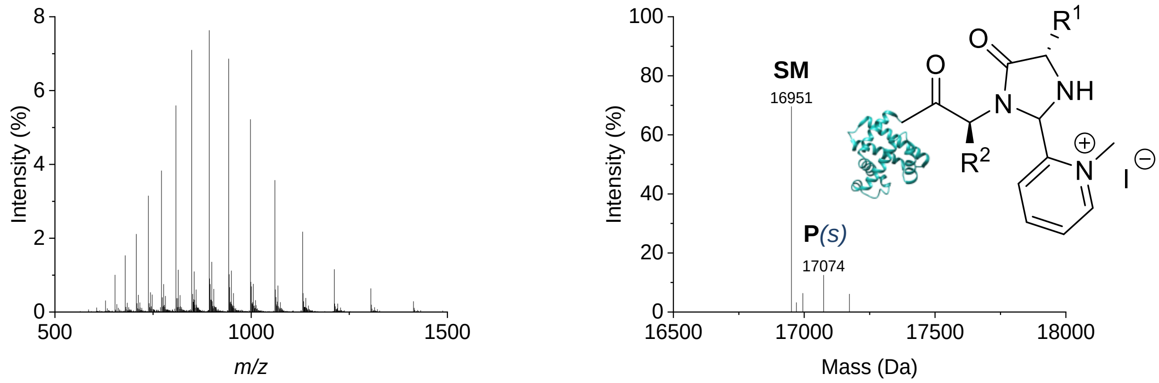

PCA 12

19%

MS (ESI$^+$) [**SM**+H]$^+$ found 16951, calculated 16951; [**SM**+H_2O+H]$^+$ found 16970, calculated 16969; [**SM**+MeCN+H]$^+$ found 16995, calculated 16992; [**P**(s)+H_2O+H]$^+$ found 17074, calculated 17073; [**P**(s)+H_2O+H_3PO_4+H]$^+$ found 17173, calculated 17171.

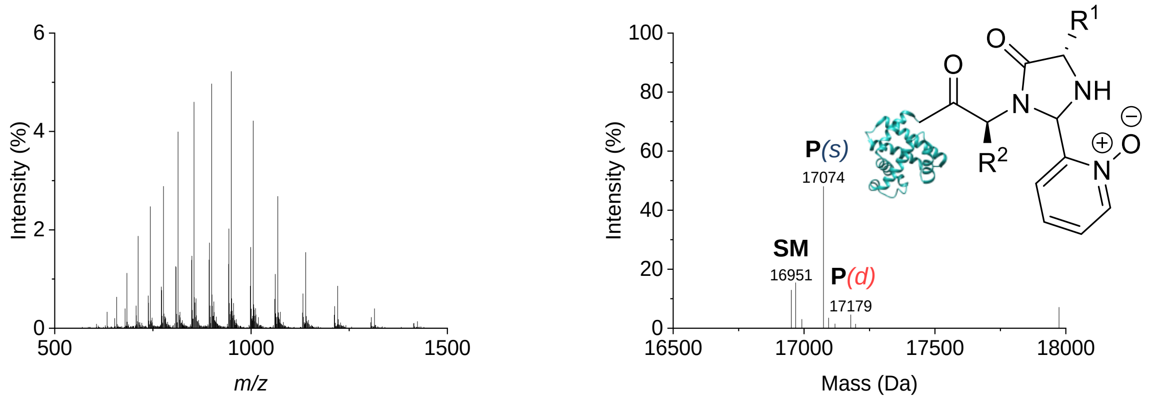

PCA 13

66%

59(s):7(d)

MS (ESI$^+$) [**SM**+H]$^+$ found 16951, calculated 16951; [**SM**+H_2O+H]$^+$ found 16968, calculated 16969; [**SM**+MeCN+H]$^+$ found 16992, calculated 16992; [**P**(s)+H_2O+H]$^+$ found 17074, calculated 17074; [**P**(s)+MeCN+H]$^+$ found 17094, calculated 17097; [**P**(s)+MeCN+Na]$^+$ found 17118, calculated 17119; [**P**(d)+H_2O+H]$^+$ found 17179, calculated 17179; [**P**(d)+2H_2O+H]$^+$ found 17197, calculated 17197.

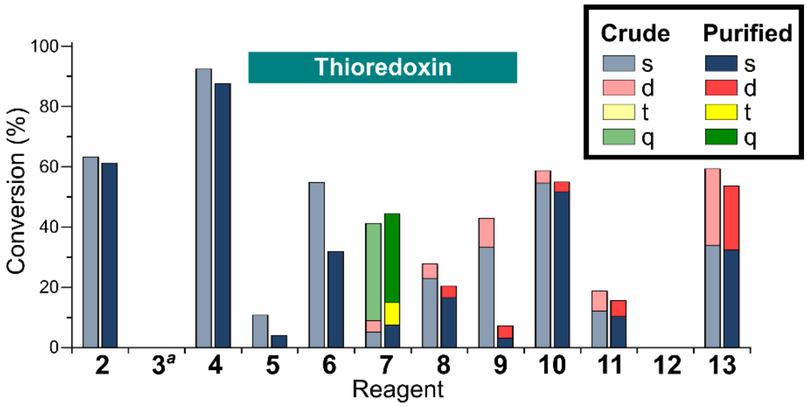

Figure S6. Conversions for the modification of thioredoxin before (crude) and after (purified) dialysis at 4 °C. s = single, d = double, t = triple, q = quadruple modification.

	PCA	Conversion (%)	
		Crude	*Purified*
Thioredoxin	2	63 *(s)*	61 *(s)*
	3	-	-
	4	92 *(s)*	88 *(s)*
	5	11 *(s)*	4 *(s)*
	6	55 *(s)*	32 *(s)*
	7	41<br>*5(s):4(d):32(q)*	45<br>*8(s):7(t):30(q)*
	8	28<br>*23(s):5(d)*	20<br>*17(s):4(d)*
	9	43<br>*33(s):10(d)*	7<br>*3(s):4(d)*
	10	59<br>*55(s):4(d)*	55<br>*52(s):3(d)*
	11	19<br>*12(s): 7(d)*	15<br>*10(s):5(d)*
	12	0	0
	13	59<br>*34(s): 25(d)*	54<br>*33(s):21(d)*

Table S6. Conversions for the modification of thioredoxin before (crude) and after (purified) dialysis at 4 °C. s = single, d = double, t = triple, q = quadruple modification.

Thioredoxin *(crude)*

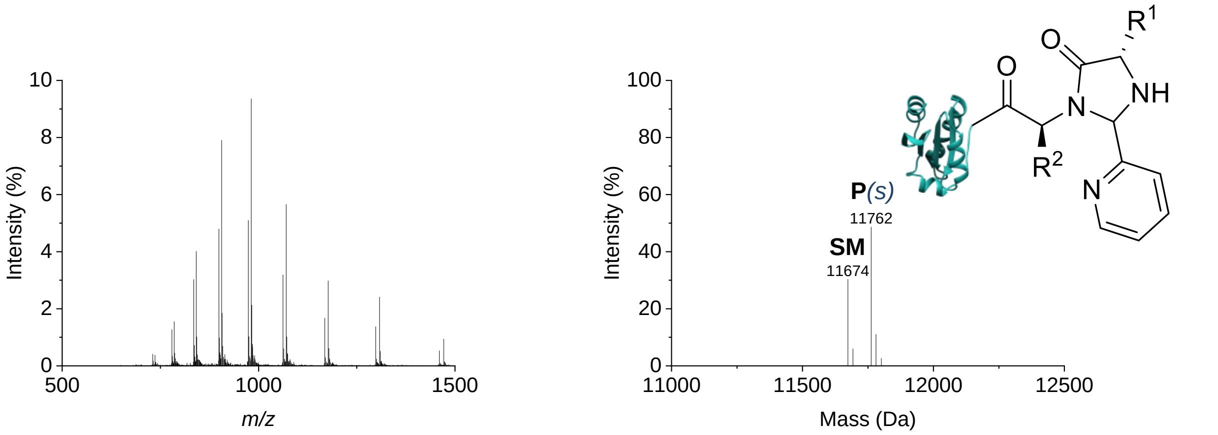

PCA 2

63%

MS (ESI$^+$) [**SM**+H]$^+$ found 11674, calculated 11674; [**SM**+H_2O+H]$^+$ found 11693, calculated 11692; [**P**(s)+H]$^+$ found 11762, calculated 11763; [**P**(s)+H_2O+H]$^+$ found 11781, calculated 11781; [**P**(s)+K]$^+$ found 11801, calculated 11801.

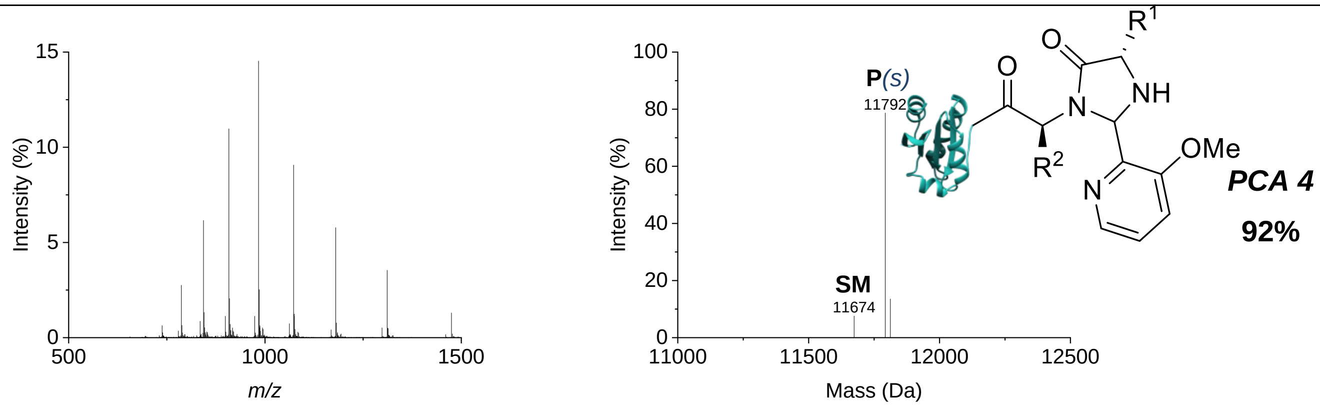

PCA 4

92%

MS (ESI$^+$) [**SM**+H]$^+$ found 11674, calculated 11674; [**P**(s)+H]$^+$ found 11792, calculated 11793; [**P**(s)+H_2O+H]$^+$ found 11812, calculated 11811.

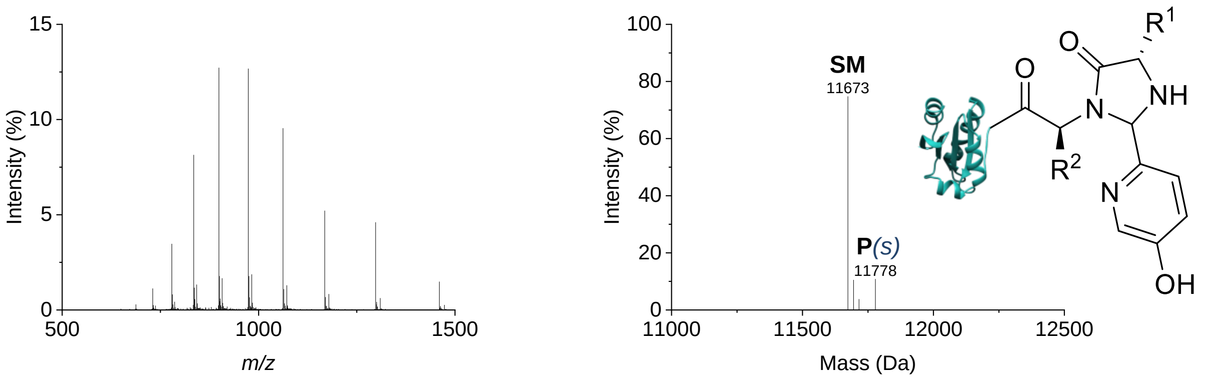

PCA 5

11%

MS (ESI$^+$) [**SM**+H]$^+$ found 11673, calculated 11674; [**SM**+H_2O+H]$^+$ found 11695, calculated 11692; [**SM**+MeCN+H]$^+$ found 11716, calculated 11715; [**P**(s)+H]$^+$ found 11778, calculated 11779.

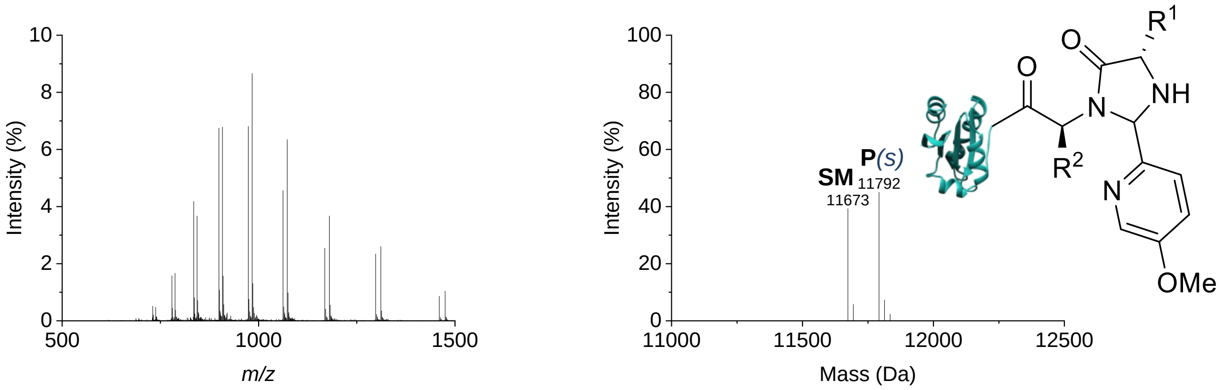

PCA 6

55%

MS (ESI$^+$) [**SM**+H]$^+$ found 11673, calculated 11674; [**SM**+H_2O+H]$^+$ found 11694, calculated 11692; [**P**(s)+H]$^+$ found 11792, calculated 11793; [**P**(s)+H_2O+H]$^+$ found 11813, calculated 11811; [**P**(s)+MeCN+H]$^+$ found 11835, calculated 11834.

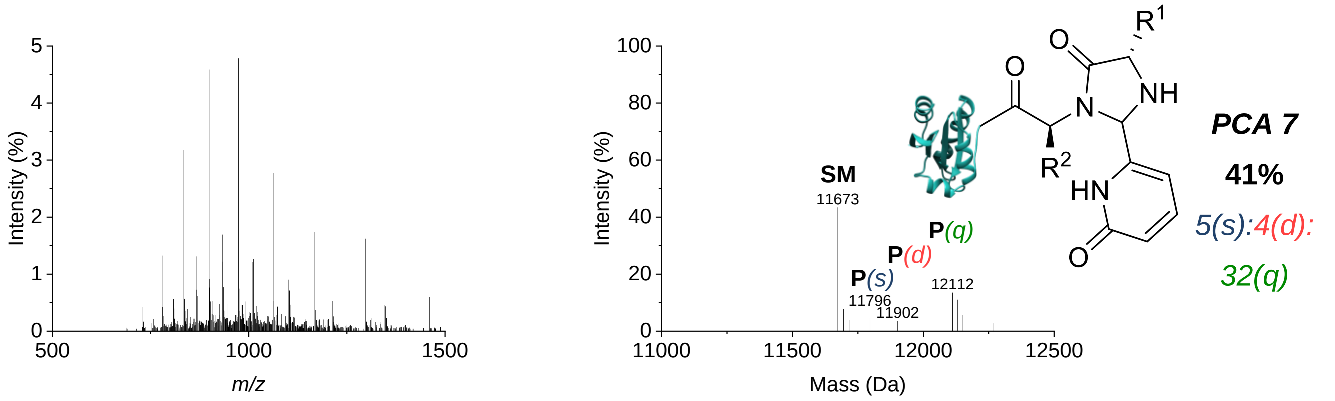

MS (ESI$^+$) [**SM**+H]$^+$ found 11673, calculated 11674; [**SM**+H_2O+H]$^+$ found 11695, calculated 11692; [**SM**+MeCN+H]$^+$ found 11716, calculated 11715; [**P**(s)+H_2O+H]$^+$ found 11796, calculated 11797; [**P**(d)+H_2O+H]$^+$ found 11902, calculated 11902; [**P**(q)+H_2O+H]$^+$ found 12112, calculated 12112; [**P**(q)+K]$^+$ found 12130, calculated 12132; [**P**(q)+H_2O+K]$^+$ found 12148, calculated 12150.

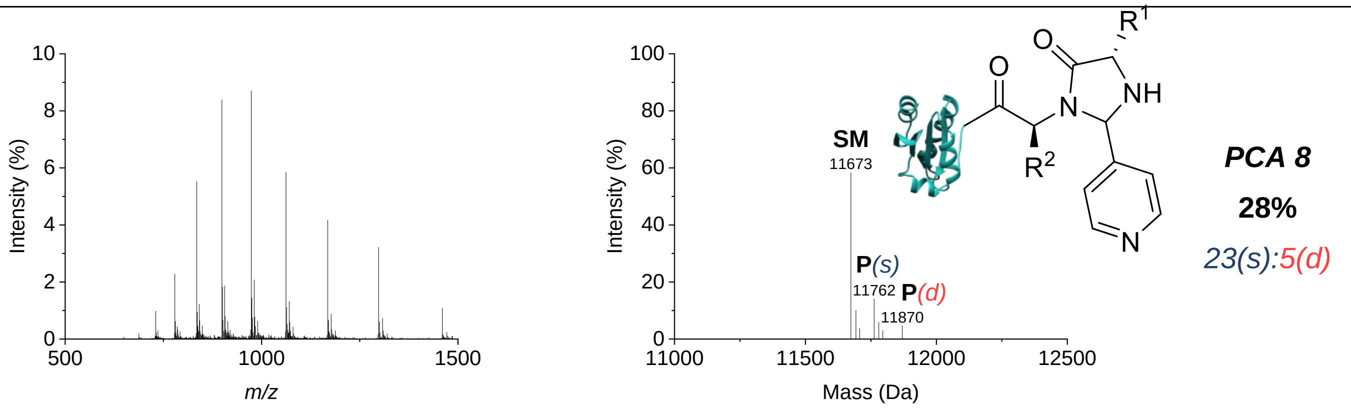

MS (ESI$^+$) [**SM**+H]$^+$ found 11673, calculated 11674; [**SM**+H_2O+H]$^+$ found 11693, calculated 11692; [**SM**+MeOH+H]$^+$ found 11707, calculated 11706; [**P**(s)+H]$^+$ found 11762, calculated 11763; [**P**(s)+H_2O+H]$^+$ found 11780, calculated 11781; [**P**+MeOH+H]$^+$ found 11796, calculated 11795; [**P**(d)+H_2O+H]$^+$ found 11870, calculated 11870.

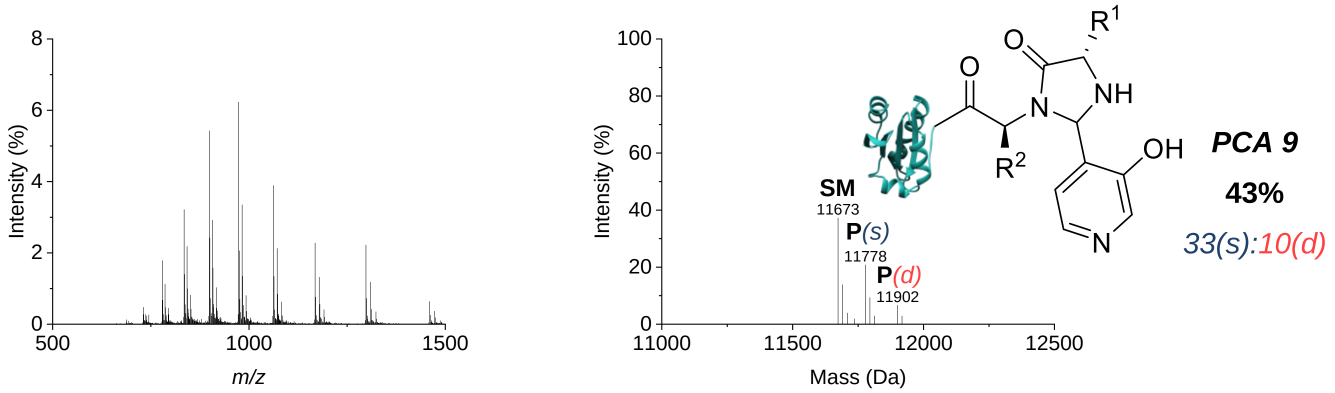

MS (ESI$^+$) [**SM**+H]$^+$ found 11673, calculated 11674; [**SM**+H_2O+H]$^+$ found 11690, calculated 11692; [**SM**+MeOH+H]$^+$ found 11709, calculated 11706; [**SM**+MeCN+Na]$^+$ found 11736, calculated 11737; [**P**(s)+H]$^+$ found 11778, calculated 11779; [**P**(s)+H_2O+H]$^+$ found 11795, calculated 11797; [**P**+MeOH+H]$^+$ found 11813, calculated 11811; [**P**(d)+H_2O+H]$^+$ found 11902, calculated 11902; [**P**(d)+MeOH+H]$^+$ found 11918, calculated 11916.

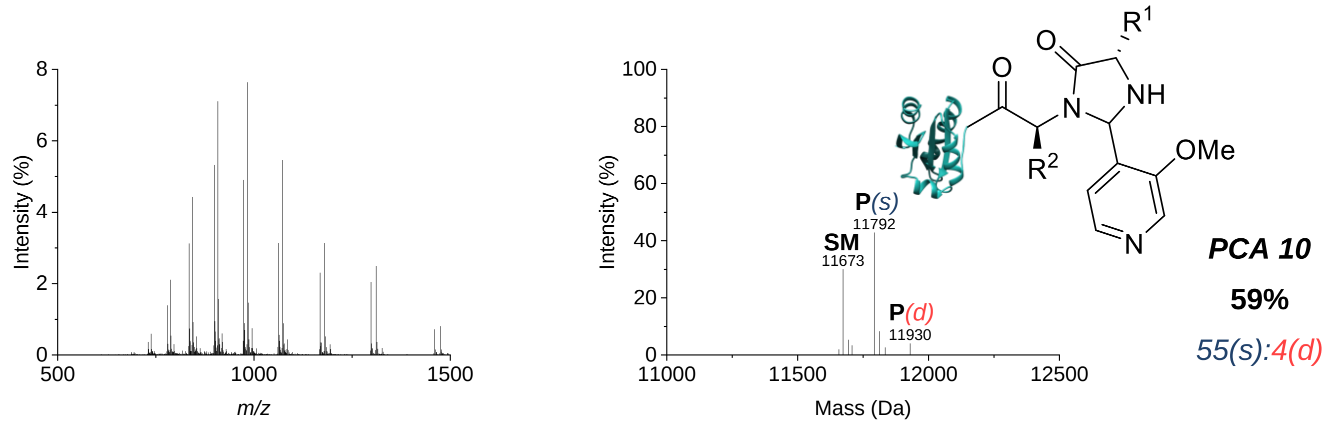

MS (ESI$^+$) [**SM**-H_2O+H]$^+$ found 11658, calculated 11656; [**SM**+H]$^+$ found 11673, calculated 11674; [**SM**+H_2O+H]$^+$ found 11695, calculated 11692; [**SM**+MeOH+H]$^+$ found 11708, calculated 11706; [**P**(s)+H]$^+$ found 11792, calculated 11793; [**P**(s)+H_2O+H]$^+$ found 11813, calculated 11811; [**P**(s)+MeCN+H]$^+$ found 11834, calculated 11834; [**P**(d)+H_2O+H]$^+$ found 11930, calculated 11930.

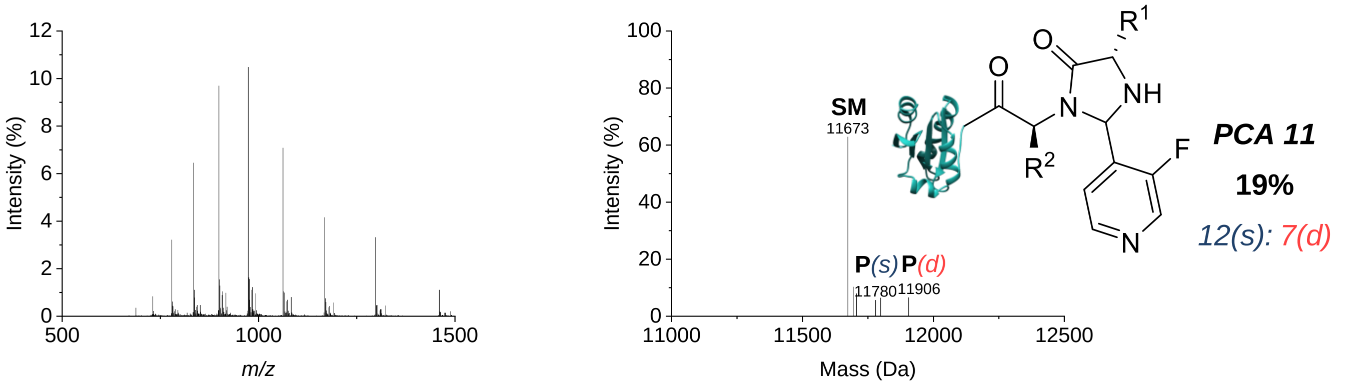

MS (ESI$^+$) [**SM**+H]$^+$ found 11673, calculated 11674; [**SM**+H_2O+H]$^+$ found 11694, calculated 11692; [**SM**+MeOH+H]$^+$ found 11706, calculated 11706; [**P**(s)+H]$^+$ found 11780, calculated 11781; [**P**(s)+H_2O+H]$^+$ found 11799, calculated 11799; [**P**(d)+H_2O+H]$^+$ found 11906, calculated 11906.

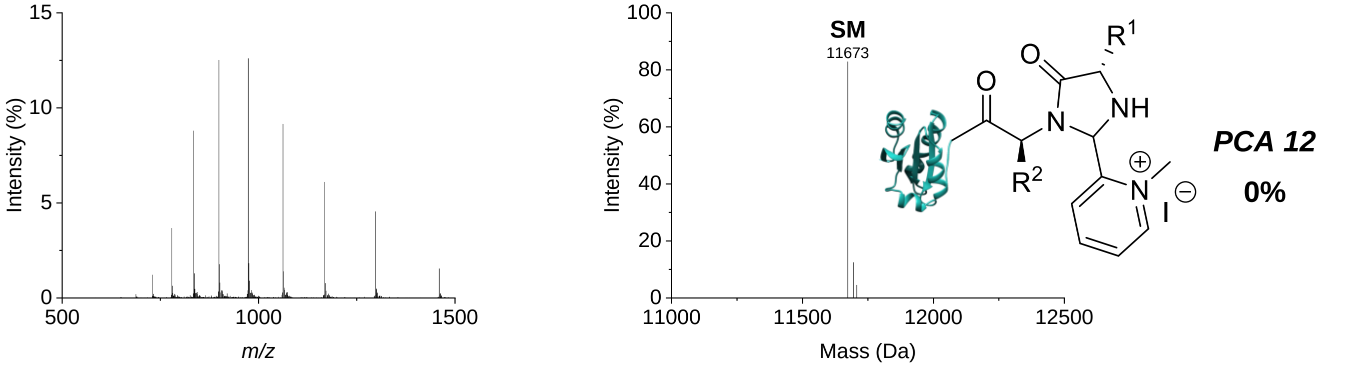

MS (ESI$^+$) [**SM**+H]$^+$ found 11673, calculated 11674; [**SM**+H_2O+H]$^+$ found 11694, calculated 11692; [**SM**+MeOH+H]$^+$ found 11707, calculated 11706.

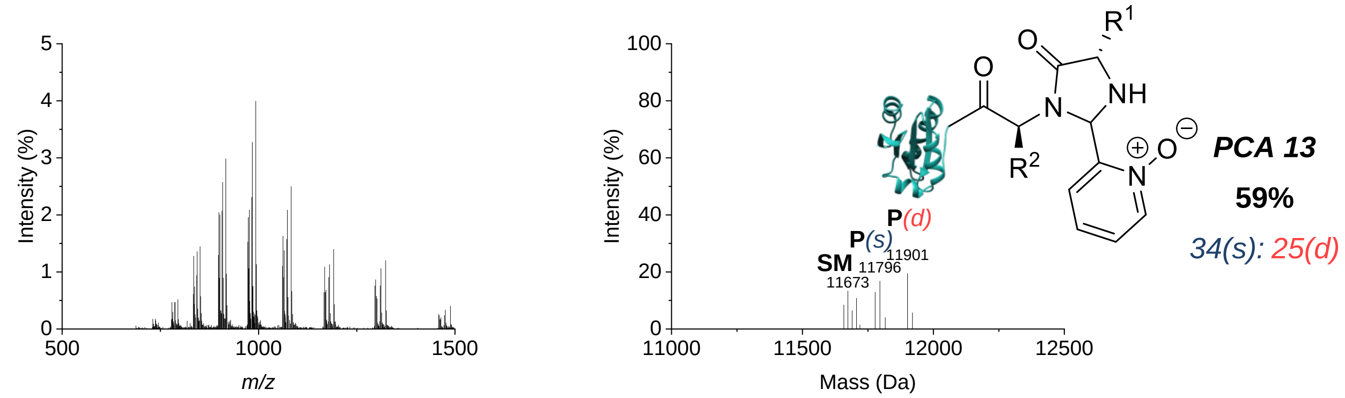

MS (ESI$^+$) [**SM**-H_2O+H]$^+$ found 11658, calculated 11656; [**SM**+H]$^+$ found 11673, calculated 11674; [**SM**+H_2O+H]$^+$ found 11690, calculated 11692; [**SM**+MeOH+H]$^+$ found 11707, calculated 11706; [**SM**+MeCN+H]$^+$ found 11719, calculated 11715; [**P**(s)+H]$^+$ found 11778, calculated 11779;

[**P**(s)+H_2O+H]$^+$ found 11796, calculated 11797; [**P**+K]$^+$ found 11816, calculated 11817; [**P**(d)+H_2O+H]$^+$ found 11901, calculated 11902; [**P**(d)+K]$^+$ found 11920, calculated 11922.

Thioredoxin *(purified)*

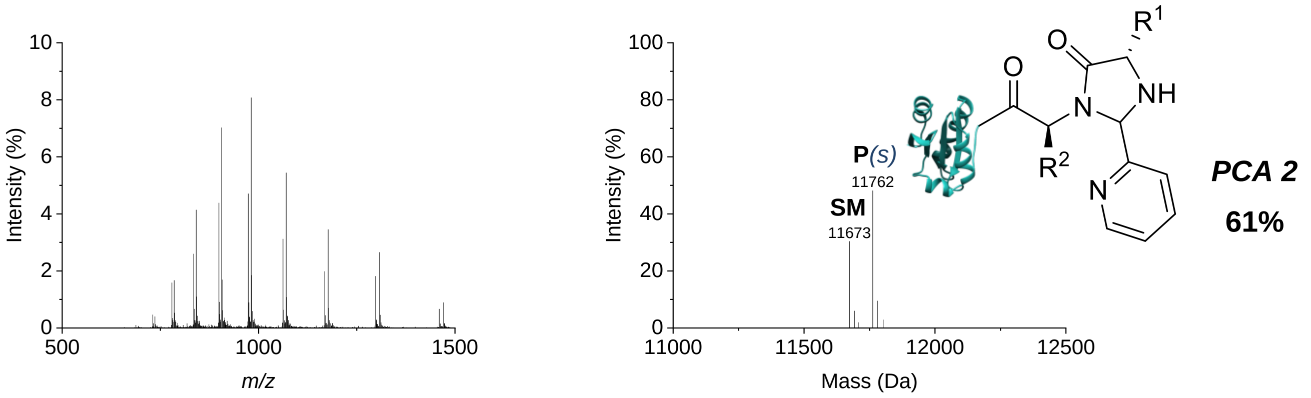

MS (ESI$^+$) [**SM**+H]$^+$ found 11673, calculated 11674; [**SM**+H_2O+H]$^+$ found 11692, calculated 11692; [**SM**+MeOH+H]$^+$ found 11707, calculated 11706; [**P**(s)+H]$^+$ found 11762, calculated 11763; [**P**(s)+H_2O+H]$^+$ found 11780, calculated 11781; [**P**(s)+K]$^+$ found 11802, calculated 11801.

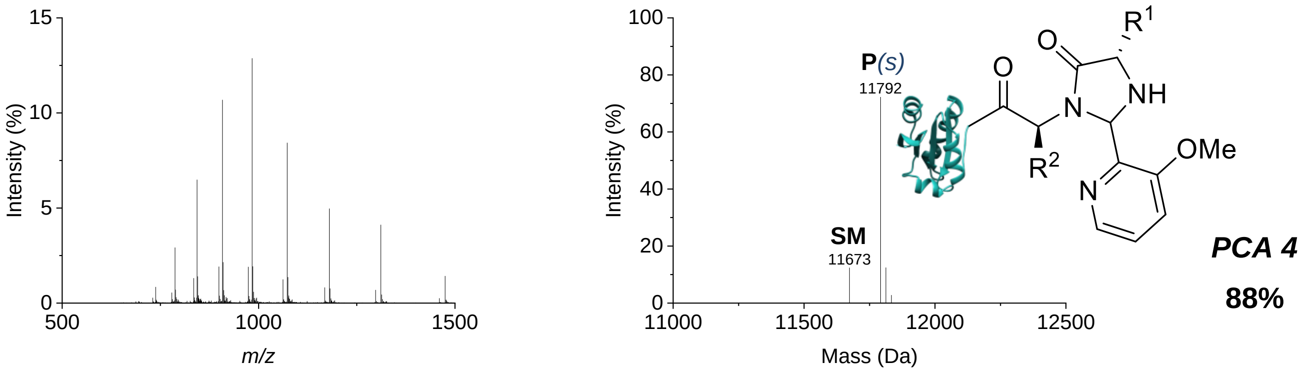

MS (ESI$^+$) [**SM**+H]$^+$ found 11673, calculated 11674; [**P**(s)+H]$^+$ found 11792, calculated 11793; [**P**(s)+H_2O+H]$^+$ found 11813, calculated 11811; [**P**(s)+MeCN+H]$^+$ found 11834, calculated 11834.

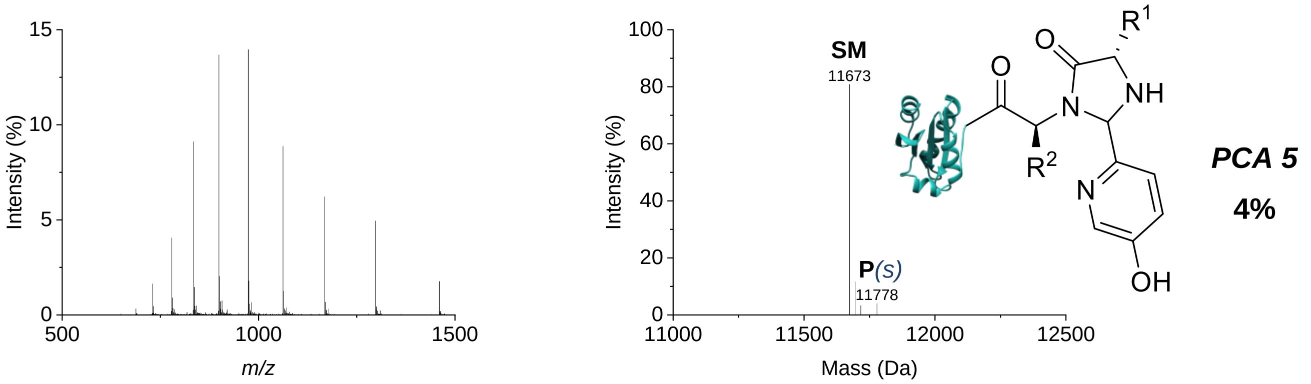

MS (ESI$^+$) [**SM**+H]$^+$ found 11673, calculated 11674; [**SM**+H_2O+H]$^+$ found 11695, calculated 11692; [**SM**+MeCN+H]$^+$ found 11717, calculated 11715; [**P**(s)+H]$^+$ found 11778, calculated 11779.

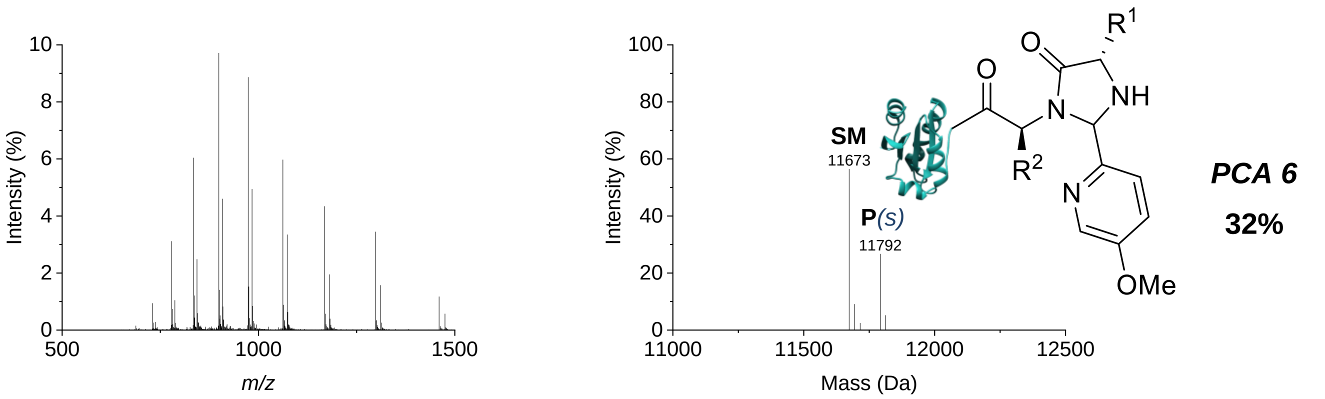

MS (ESI$^+$) [**SM**+H]$^+$ found 11673, calculated 11674; [**SM**+H_2O+H]$^+$ found 11694, calculated 11692; [**SM**+MeCN+H]$^+$ found 11715, calculated 11715; [**P**(s)+H]$^+$ found 11792, calculated 11793; [**P**(s)+H_2O+H]$^+$ found 11812, calculated 11811.

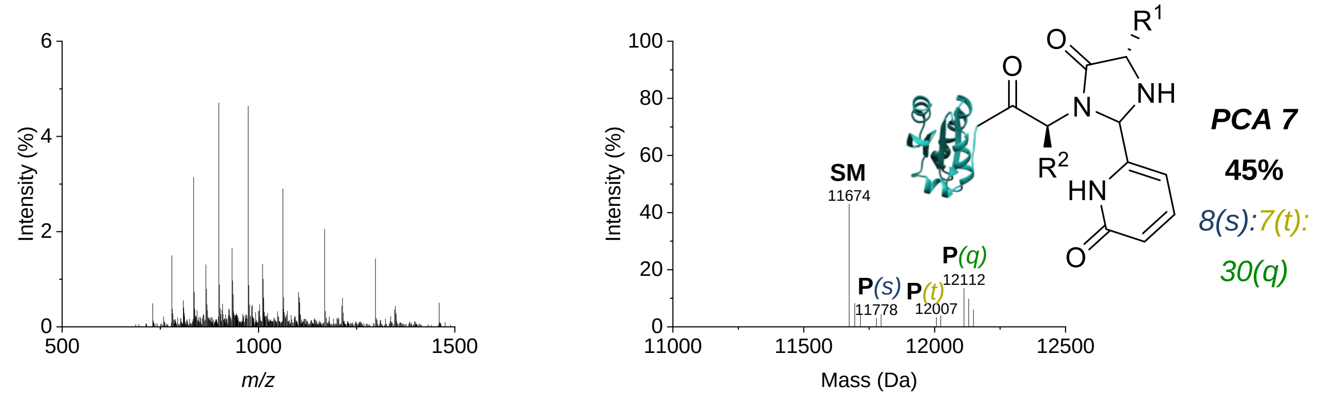

MS (ESI$^+$) [**SM**+H]$^+$ found 11674, calculated 11674; [**SM**+H_2O+H]$^+$ found 11695, calculated 11692; [**SM**+MeCN+H]$^+$ found 11716, calculated 11715; [**P**(s)+H]$^+$ found 11778, calculated 11779; [**P**(s)+H_2O+H]$^+$ found 11795, calculated 11797; [**P**(t)+H_2O+H]$^+$ found 12007, calculated 12007; [**P**(t)+MeOH+H]$^+$ found 12024, calculated 12021; [**P**(q)+H_2O+H]$^+$ found 12112, calculated 12112; [**P**(q)+K]$^+$ found 12130, calculated 12132; [**P**(q)+H_2O+K]$^+$ found 12148, calculated 12150.

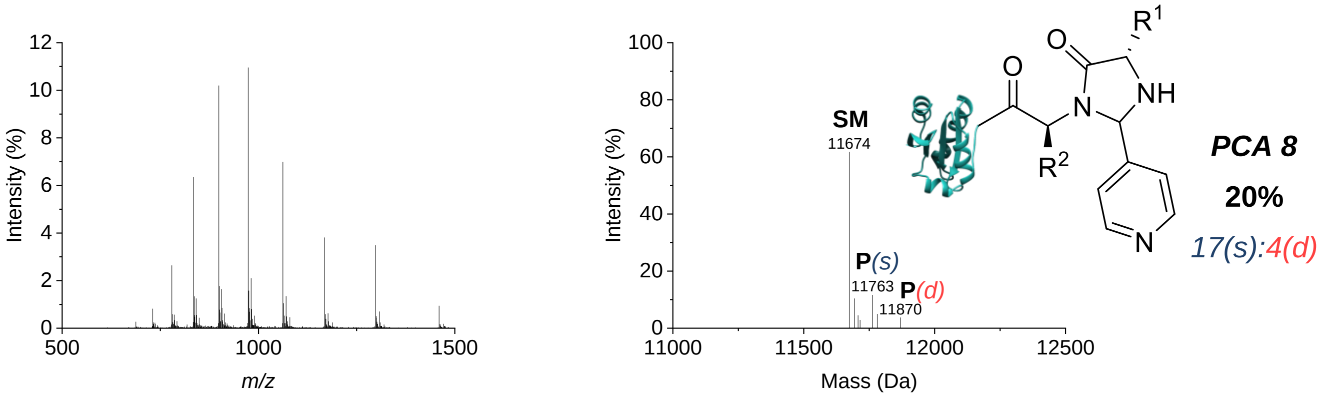

MS (ESI$^+$) [**SM**+H]$^+$ found 11674, calculated 11674; [**SM**+H_2O+H]$^+$ found 11694, calculated 11692; [**SM**+MeOH+H]$^+$ found 11708, calculated 11706; [**SM**+MeCN+H]$^+$ found 11715, calculated 11715; [**P**(s)+H]$^+$ found 11763, calculated 11763; [**P**(s)+H_2O+H]$^+$ found 11781, calculated 11781; [**P**(d)+H_2O+H]$^+$ found 11870, calculated 11870.

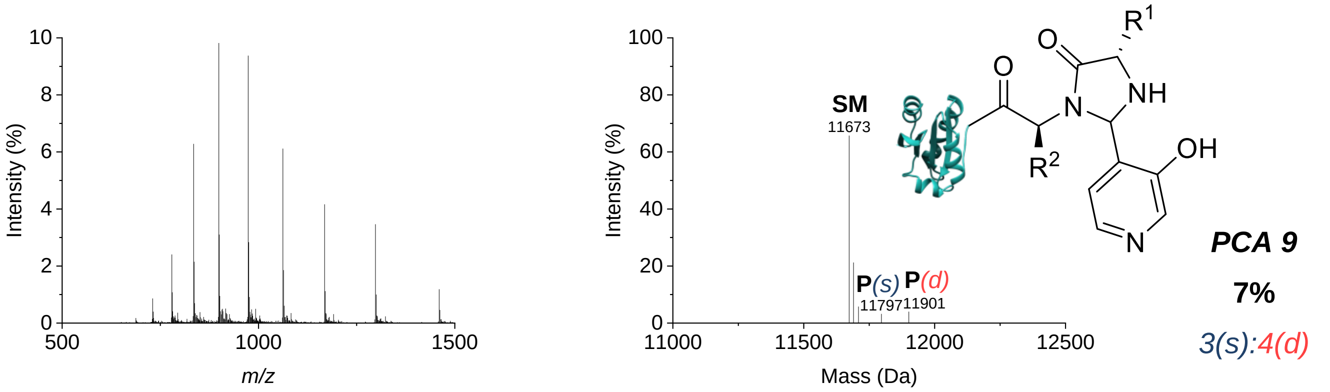

MS (ESI$^+$) [**SM**+H]$^+$ found 11673, calculated 11674; [**SM**+H_2O+H]$^+$ found 11690, calculated 11692; [**SM**+MeOH+H]$^+$ found 11709, calculated 11706; [**P**(s)+H_2O+H]$^+$ found 11797, calculated 11797; [**P**(d)+H_2O+H]$^+$ found 11901, calculated 11902.

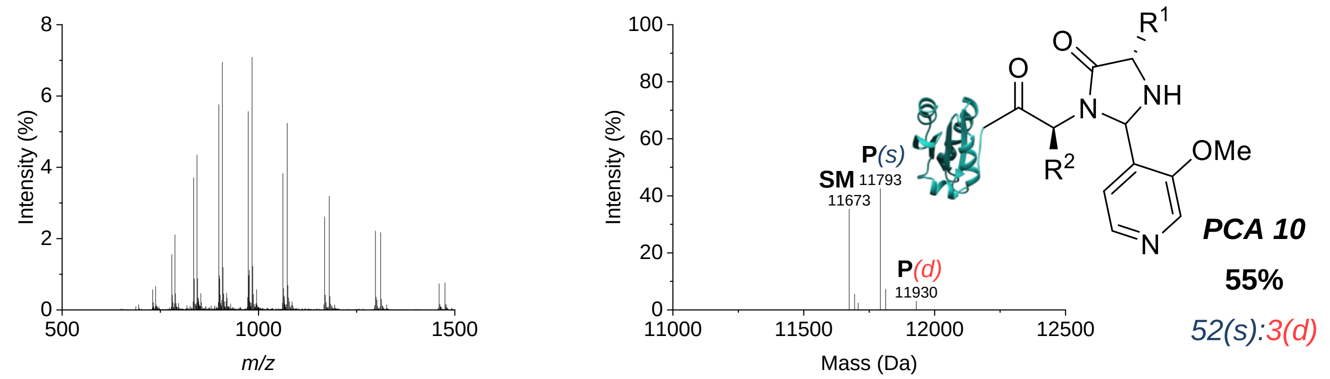

MS (ESI$^+$) [**SM**+H]$^+$ found 11673, calculated 11674; [**SM**+H_2O+H]$^+$ found 11694, calculated 11692; [**SM**+MeOH+H]$^+$ found 11708, calculated 11706; [**P**(s)+H]$^+$ found 11793, calculated 11793; [**P**(s)+H_2O+H]$^+$ found 11813, calculated 11811; [**P**(d)+H_2O+H]$^+$ found 11930, calculated 11930.

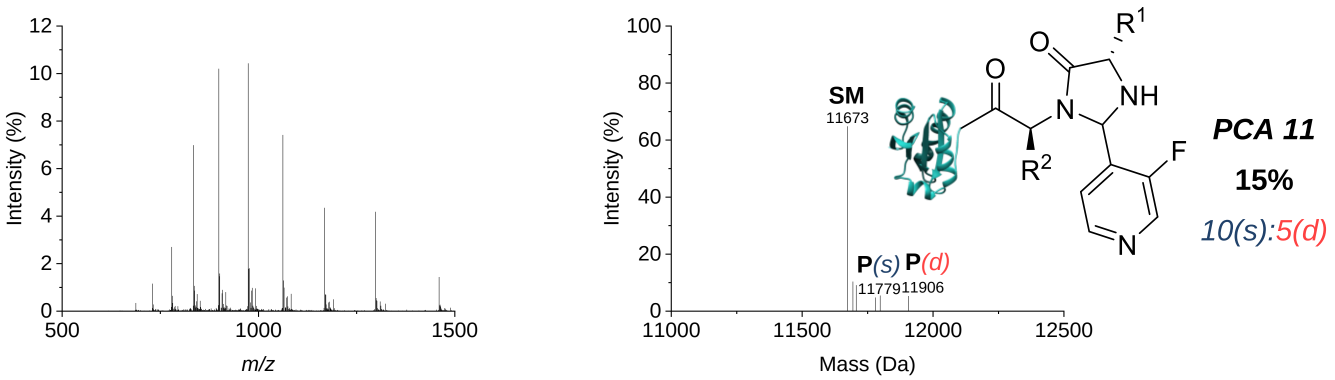

MS (ESI$^+$) [**SM**+H]$^+$ found 11673, calculated 11674; [**SM**+H_2O+H]$^+$ found 11694, calculated 11692; [**SM**+MeOH+H]$^+$ found 11707, calculated 11706; [**P**(s)+H]$^+$ found 11779, calculated 11781; [**P**(s)+H_2O+H]$^+$ found 11798, calculated 11799; [**P**(d)+H_2O+H]$^+$ found 11906, calculated 11906.

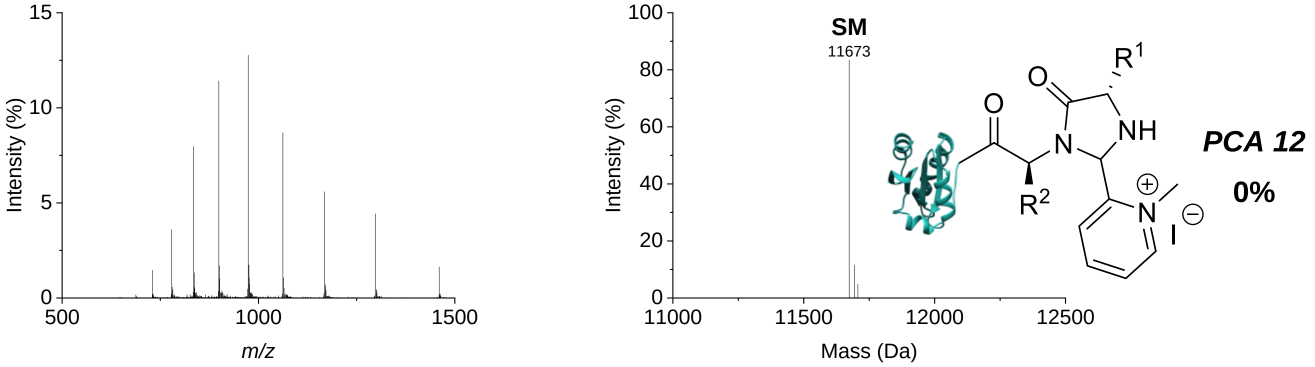

MS (ESI$^+$) [**SM**+H]$^+$ found 11673, calculated 11674; [**SM**+H_2O+H]$^+$ found 11695, calculated 11692; [**SM**+MeOH+H]$^+$ found 11707, calculated 11706.

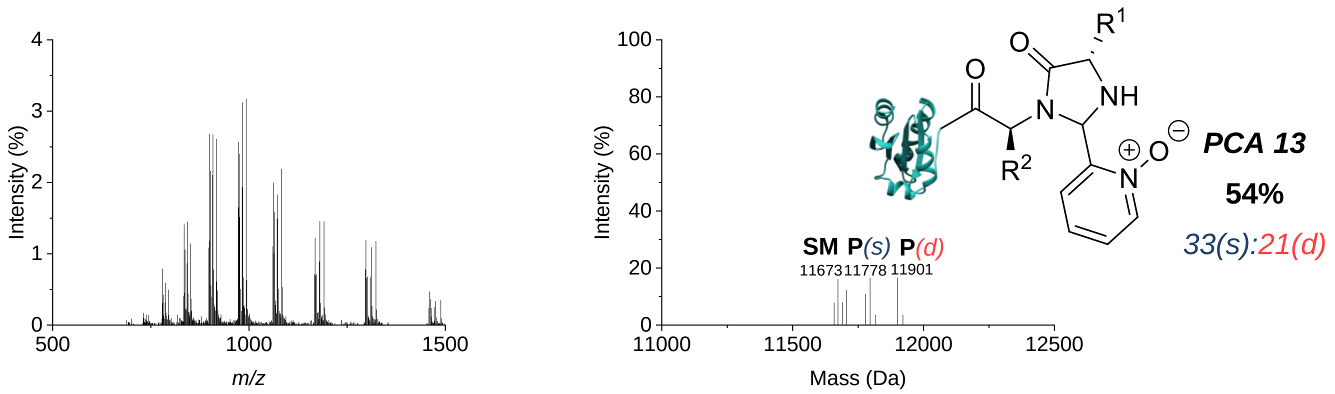

MS (ESI+) [**SM**-H_2O+H]+ found 11658, calculated 11656; [**SM**+H]+ found 11673, calculated 11674; [**SM**+H_2O+H]+ found 11690, calculated 11692; [**SM**+MeOH+H]+ found 11706, calculated 11706; [**P**(s)+H]+ found 11778, calculated 11779; [**P**(s)+H_2O+H]+ found 11796, calculated 11797; [**P**+K]+ found 11815, calculated 11817; [**P**(d)+H_2O+H]+ found 11901, calculated 11902; [**P**(d)+K]+ found 11921, calculated 11922.

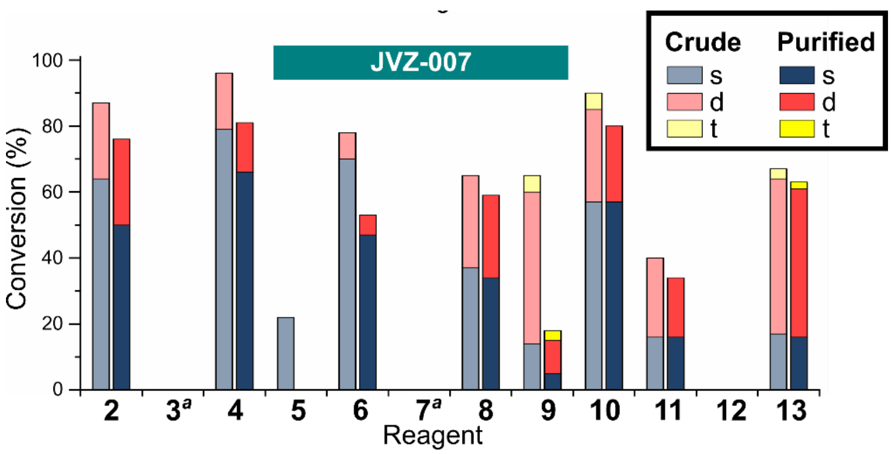

Figure S6. Conversions for the modification of JVZ-007 before (crude) and after (purified) dialysis at 4 °C. s = single, d = double, t = triple modification.

	**PCA**	**Conversion (%)** *Crude*	*Purified*
**JVZ-007**	**2**	**87**<br>*64(s):23(d)*	**76**<br>*50(s):26(d)*
	**3**	**-**	**-**
	**4**	**96**<br>*79(s):17(d)*	**76**<br>*61(s):15(d)*
	**5**	**22** *(s)*	**0**
	**6**	**78**<br>*70(s):8(d)*	**53**<br>*47(s):6(d)*
	**7**	**-**	**-**
	**8**	**65**<br>*37(s):28(d)*	**59**<br>*34(s):25(d)*
	**9**	**67**<br>*14(s):46(d):5(t)*	**18**<br>*5(s):10(d):3(t)*
	**10**	**89**<br>*57(s):28(d):4(t)*	**80**<br>*57(s):23(d)*
	**11**	**40**	**34**

		16(s):24(d)	16(s):18(d)
	12	0	4
	13	68<br>17(s): 48(d):3(t)	65<br>17(s):46(d):2(t)

Table S6. Conversions for the modification of JVZ-007 before (crude) and after (purified) dialysis at 4 °C. s = single, d = double, t = triple modification.

JVZ-007 *(crude)*

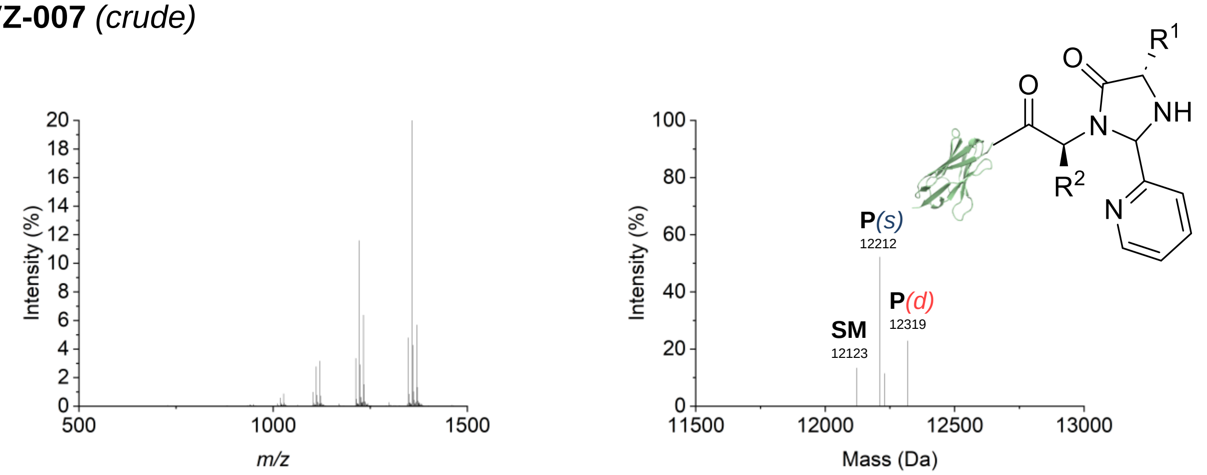

PCA 2

87%

64(s):23(d)

MS (ESI$^+$) [**SM**+H]$^+$ found 12123, calculated 12124; [**P**(s)+H]$^+$ found 12212, calculated 12213; [**P**(s)+H_2O+H]$^+$ found 12229, calculated 12231; [**P**(d)+H_2O]$^+$ found 12319, calculated 12320.

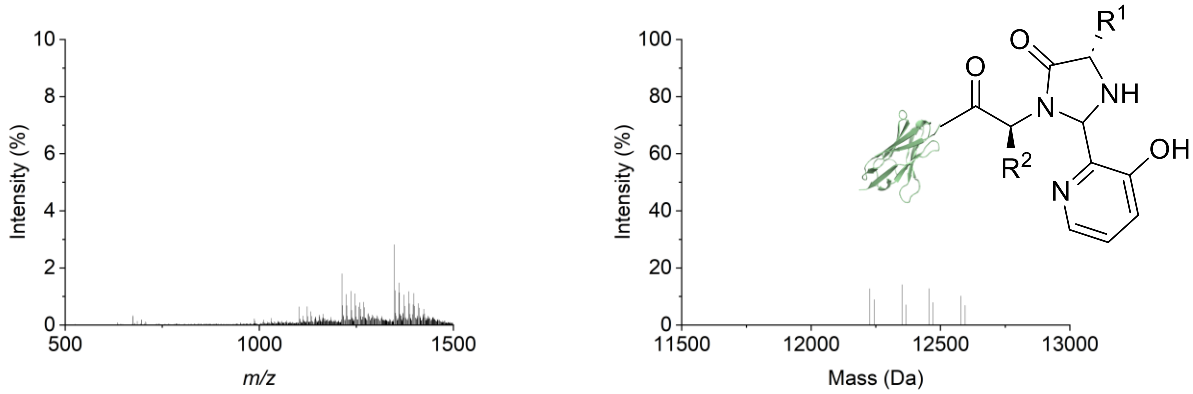

PCA 3

-

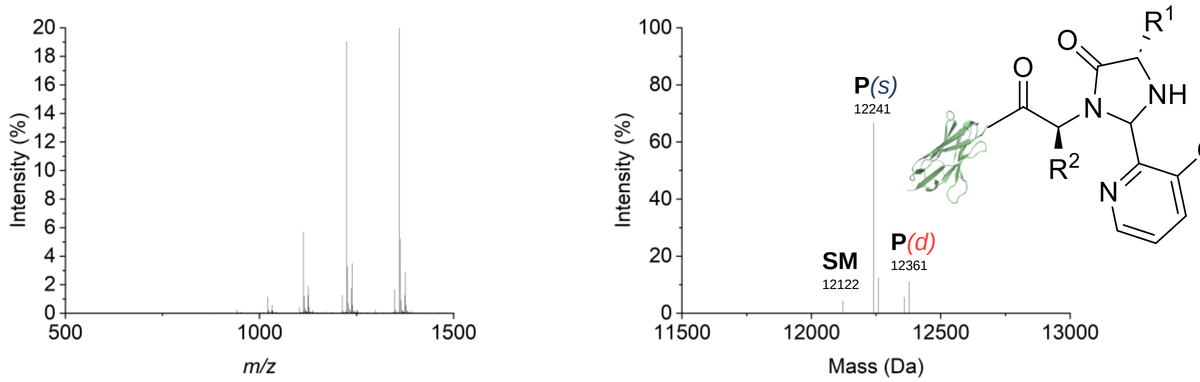

PCA 4

96%

79(s):17(d)

MS (ESI$^+$) [**SM**+H]$^+$ found 12122, calculated 12124; [**P**(s)+H]$^+$ found 12241, calculated 12243; [**P**(s)+H+H_2O]$^+$ found 12258, calculated 12261; [**P**(d)+H]$^+$ found 12361, calculated 12362; [**P**(d)+H+H_2O]$^+$ found 12379, calculated 12380.

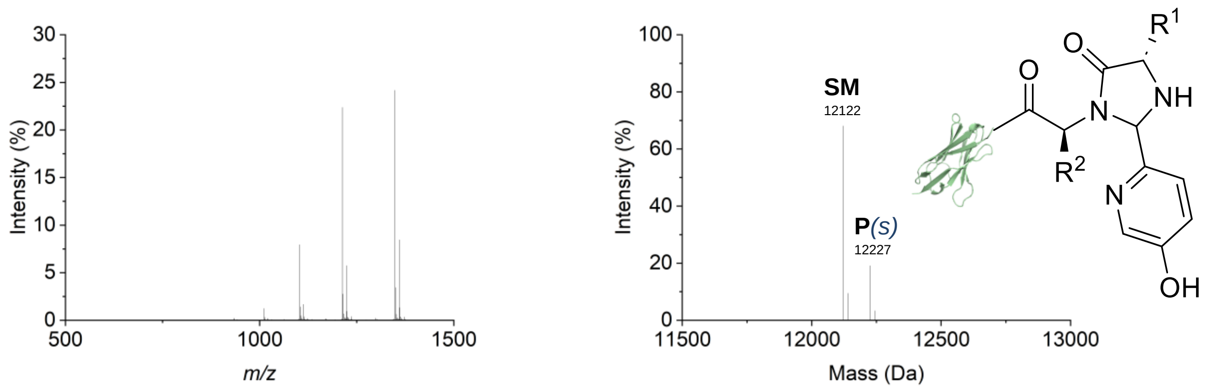

PCA 5

22%

MS (ESI+) [**SM**+H]+ found 12122, calculated 12124; [**SM**+H_2O+H]+ found 12140, calculated 12142; [**P**(s)+H]+ found 12227, calculated 12229; [**P**(s)+H_2O+H]+ found 12244, calculated 12247.

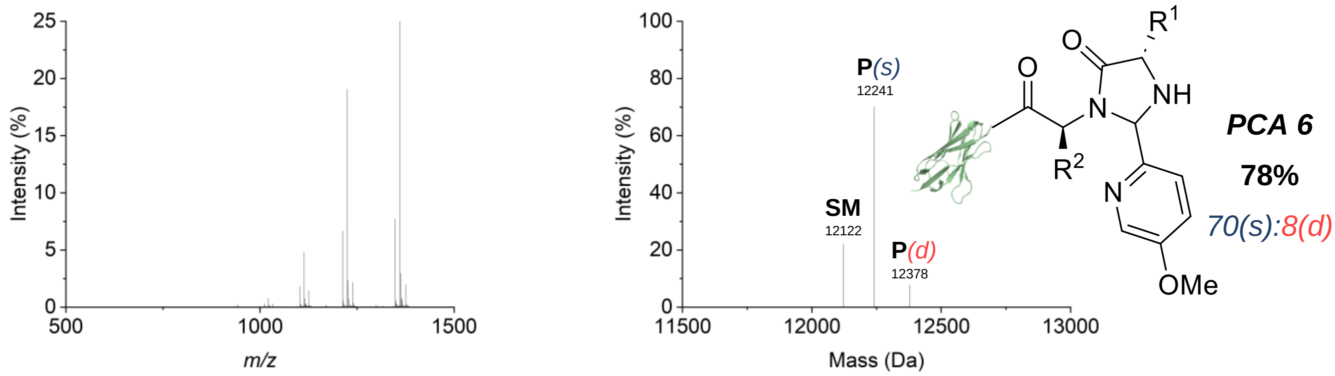

PCA 6

78%

70(s):8(d)

MS (ESI+) [**SM**+H]+ found 12122, calculated 12124; [**P**(s)+H]+ found 12241, calculated 12243; [**P**(d)+H_2O+H]+ found 12378, calculated 12380.

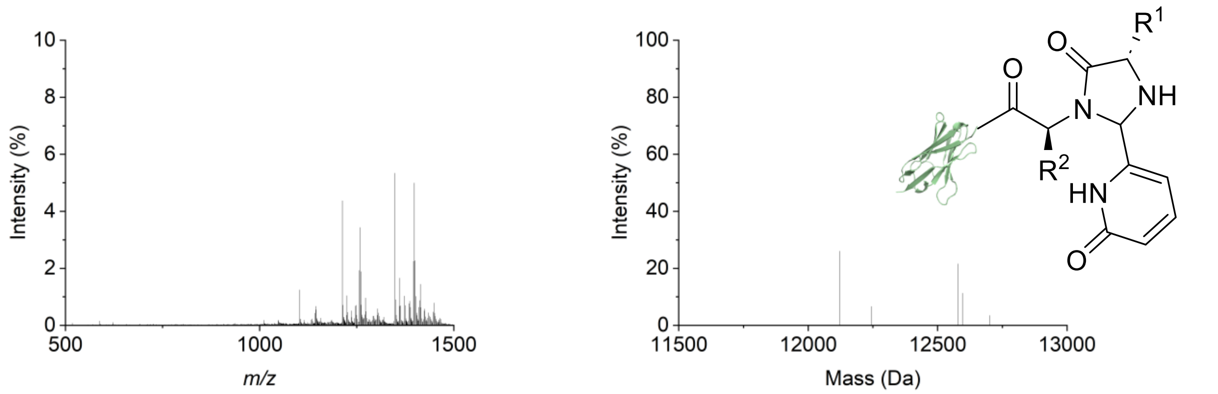

PCA 7

-

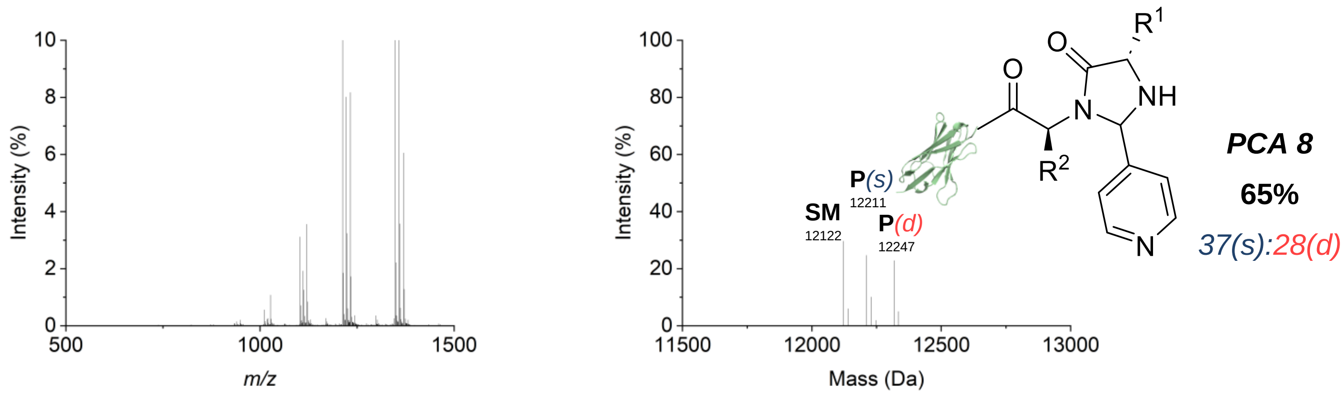

PCA 8

65%

37(s):28(d)

MS (ESI+) [**SM**+H]+ found 12122, calculated 12124; [**SM**+H_2O+H]+ found 12139, calculated 12142; [**P**(s)+H]+ found 12211, calculated 12213; [**P**(s)+H_2O+H]+ found 12228, calculated 12231; [**P**(s)+2H_2O+H]+ found 12247, calculated 12249; [**P**(d)+H_2O+H]+ found 12318, calculated 12320; [**P**(d)+2H_2O+H]+ found 12335, calculated 12338.

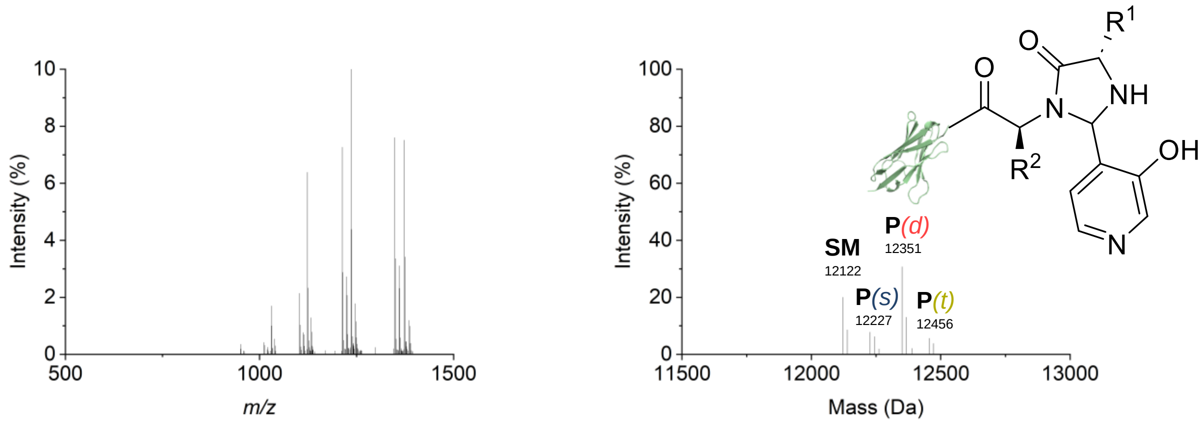

PCA 9

67%

16(s):

46(d):5(t)

MS (ESI$^+$) [**SM**+H]$^+$ found 12122, calculated 12124; [**SM**+H_2O+H]$^+$ found 12139, calculated 12142; [**P**(s)+H]$^+$ found 12227, calculated 12229; [**P**(s)+H_2O+H]$^+$ found 12245, calculated 12247; [**P**(s)+2H_2O+H]$^+$ found 12262, calculated 12265; [**P**(d)+H_2O+H]$^+$ found 12351, calculated 12352; [**P**(d)+2H_2O+H]$^+$ found 12367, calculated 12370; [**P**(d)+H_2O+K]$^+$ found 12390, calculated 12390; [**P**(t)+H_2O+H]$^+$ found 12456, calculated 12457; [**P**(t)+2H_2O+H]$^+$ found 12473, calculated 12475.

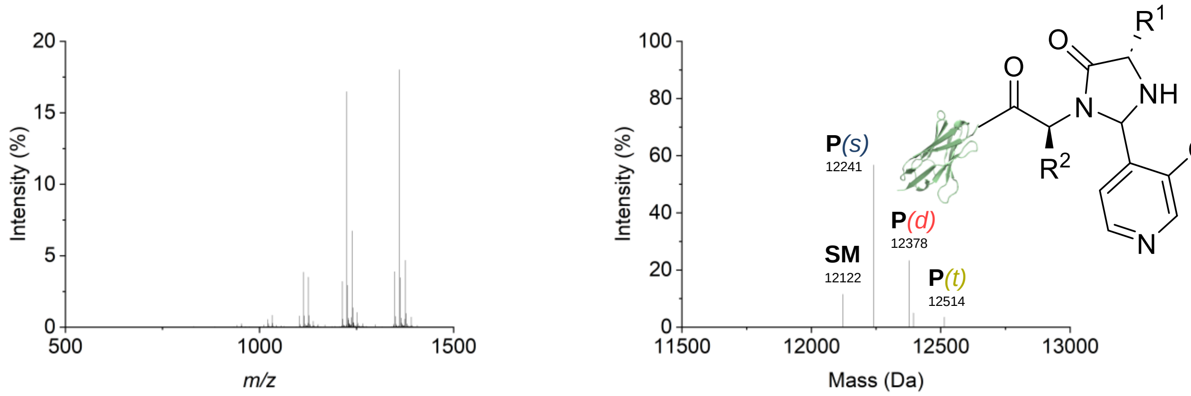

PCA 10

89%

57(s):

28(d):4(t)

MS (ESI$^+$) [**SM**+H]$^+$ found 12122, calculated 12124; [**P**(s)+H]$^+$ found 12241, calculated 12243; [**P**(d)+H_2O+H]$^+$ found 12378, calculated 12380; [**P**(d)+2H_2O+H]$^+$ found 12395, calculated 12398; [**P**(t)+2H_2O+H]$^+$ found 12514, calculated 12517.

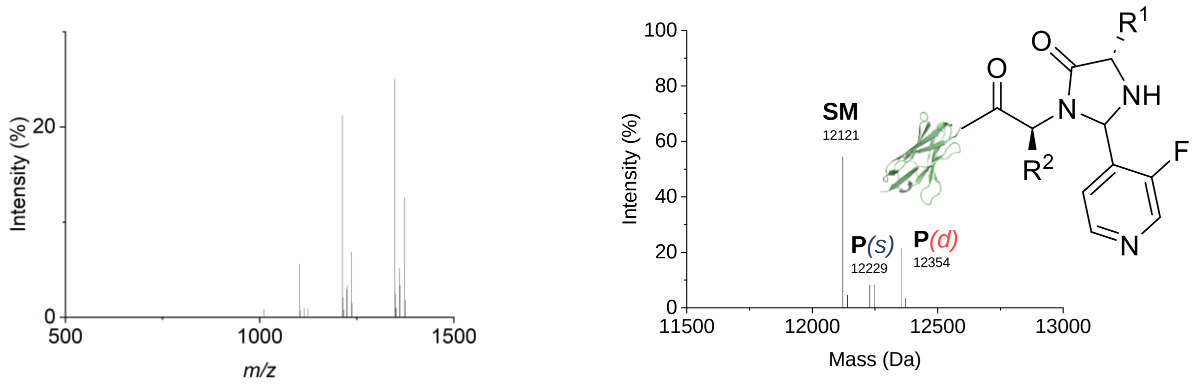

PCA 11

40%

16(s): 24(d)

MS (ESI$^+$) [**SM**+H]$^+$ found 12121, calculated 12124; [**P**(s)+H]$^+$ found 12229, calculated 12231; [**P**(s)+H_2O+H]$^+$ found 12247, calculated 12249; [**P**(d)+H_2O+H]$^+$ found 12354, calculated 12356.

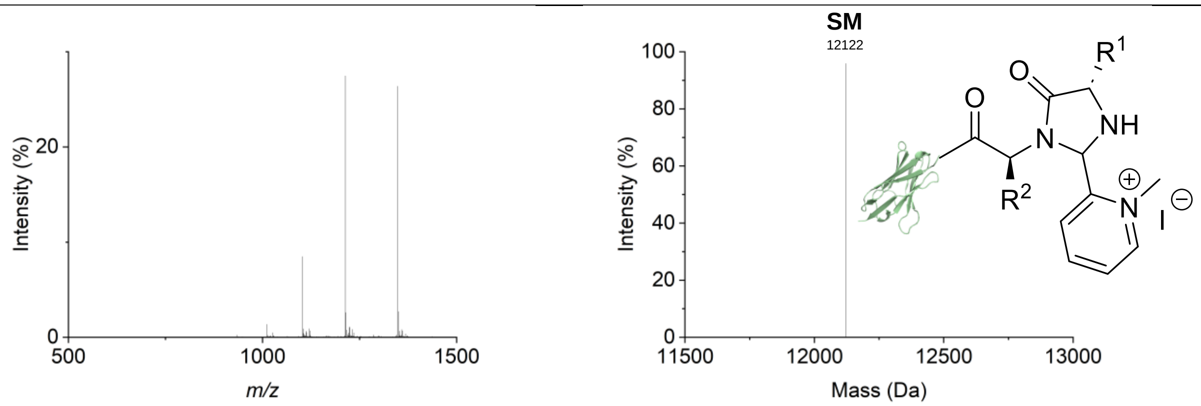

PCA 12

0%

MS (ESI$^+$) [SM+H]$^+$ found 12122, calculated 12124.

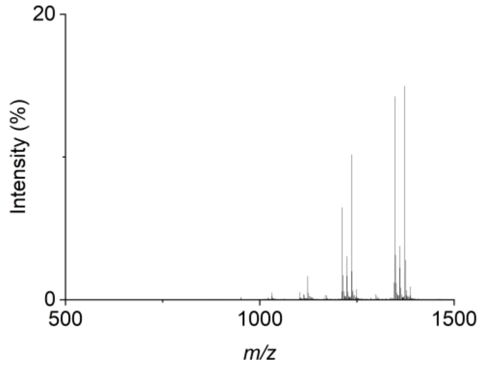

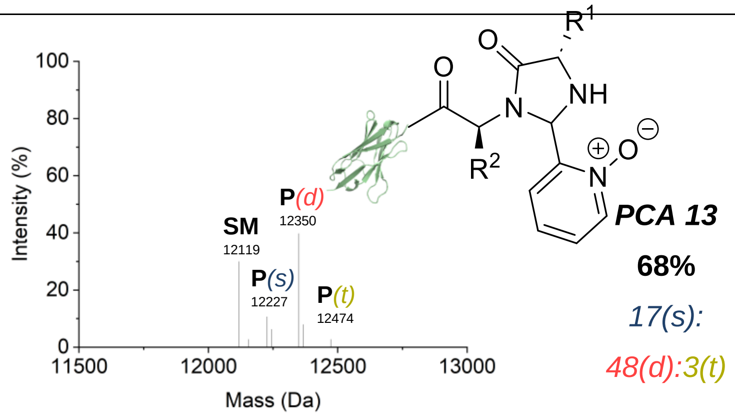

MS (ESI$^+$) [**SM**+H]$^+$ found 12119, calculated 12124; [**SM**+MeOH+H]$^+$ found 12155, calculated 12159; [**P**(s)+H]$^+$ found 12227, calculated 12229; [**P**(s)+H_2O+H]$^+$ found 12244, calculated 12247; [**P**(d)+H_2O+H]$^+$ found 12350, calculated 12352; [**P**(d)+2H_2O+H]$^+$ found 12368, calculated 12370; [**P**(t)+2H_2O+H]$^+$ found 12474, calculated 12475.

JVZ-007 *(purified)*

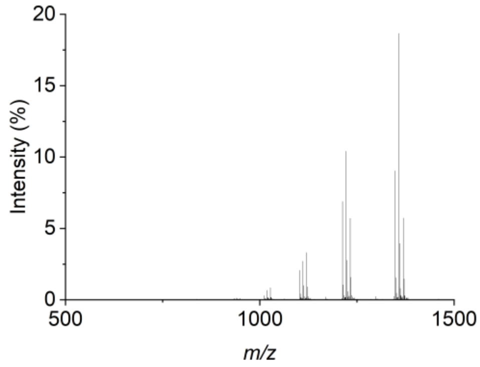

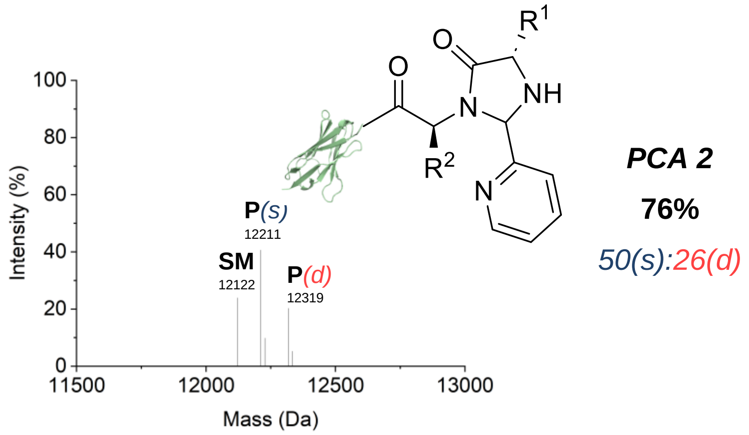

MS (ESI$^+$) [**SM**+H]$^+$ found 12122, calculated 12124; [**P**(s)+H]$^+$ found 12211, calculated 12213; [**P**(s)+H_2O+H]$^+$ found 12228, calculated 12231; [**P**(d)+H_2O]$^+$ found 12319, calculated 12320; [**P**(d)+2H_2O]$^+$ found 12335, calculated 12338.

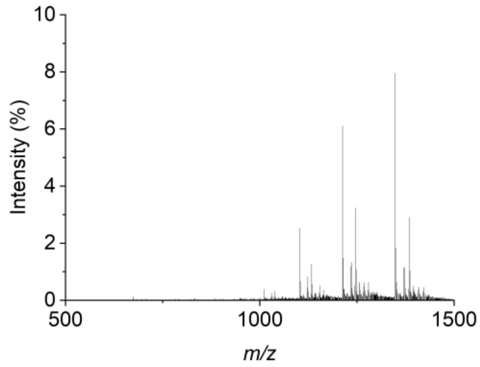

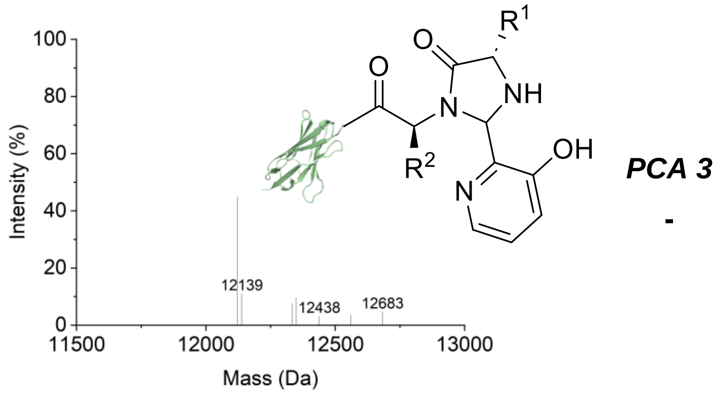

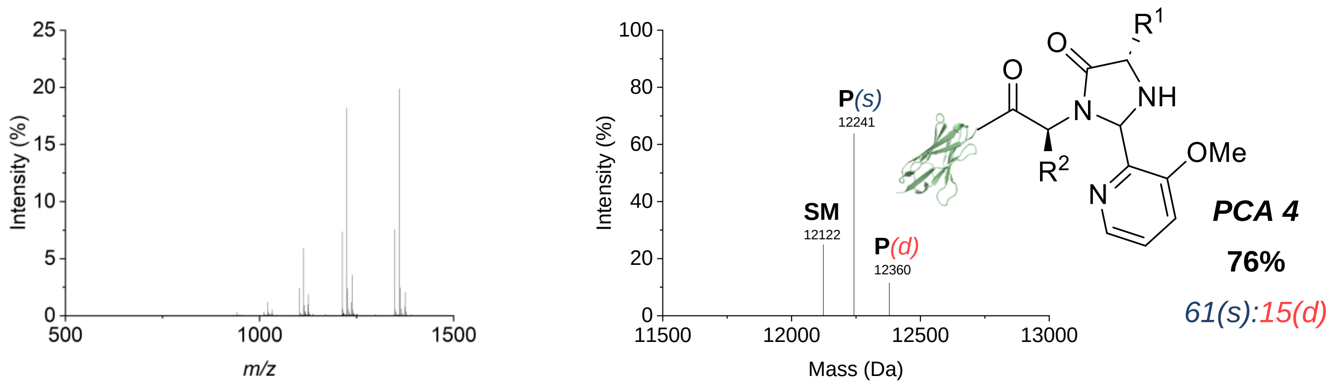

MS (ESI$^+$) [**SM**+H]$^+$ found 12122, calculated 12124; [**P**(s)+H]$^+$ found 12241, calculated 12243; [**P**(d)+H]$^+$ found 12360, calculated 12362; [**P**(d)+H+H_2O]$^+$ found 12378, calculated 12380.

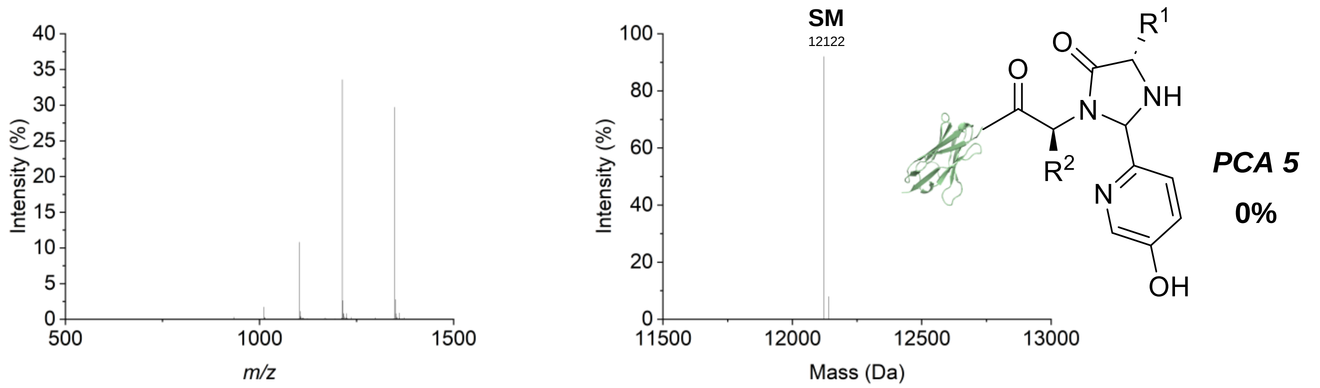

MS (ESI$^+$) [**SM**+H]$^+$ found 12122, calculated 12124; [**SM**+H_2O+H]$^+$ found 12140, calculated 12142.

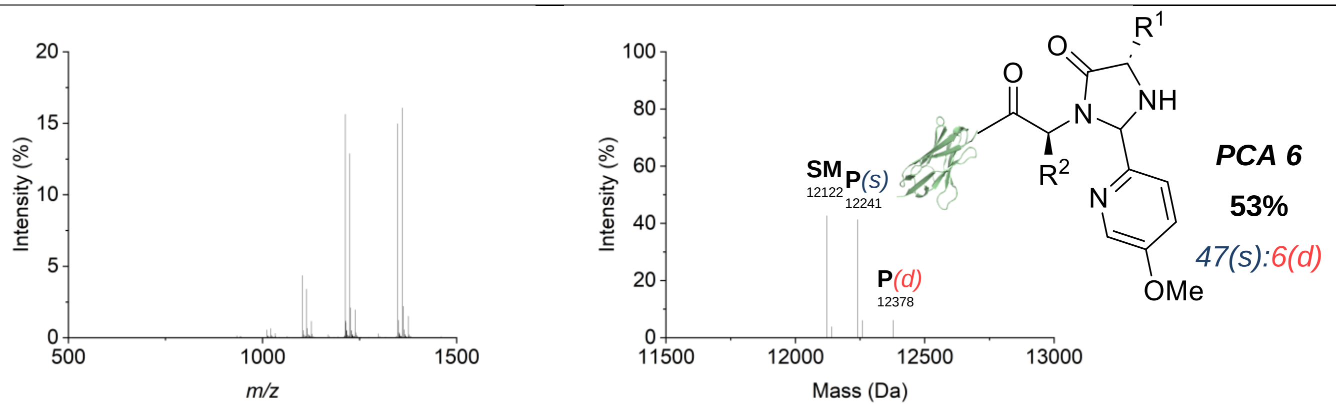

MS (ESI$^+$) [**SM**+H]$^+$ found 12122, calculated 12124; [**SM**+H_2O+H]$^+$ found 12140, calculated 12142; [**P**(s)+H]$^+$ found 12241, calculated 12243; [**P**(s)+H_2O+H]$^+$ found 12258, calculated 12261; [**P**(d)+H_2O+H]$^+$ found 12378, calculated 12380.

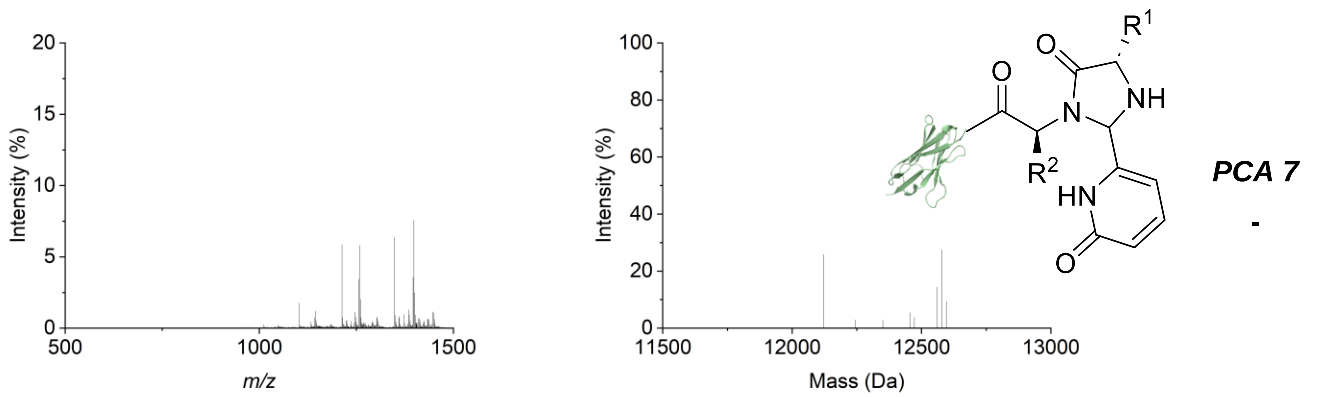

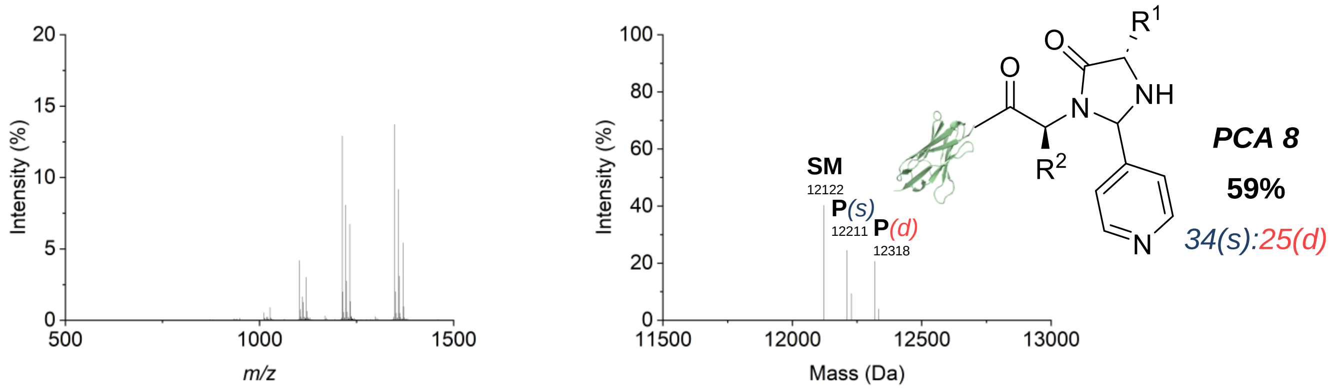

MS (ESI$^+$) [**SM**+H]$^+$ found 12122, calculated 12124; [**P**(s)+H]$^+$ found 12211, calculated 12213; [**P**(s)+H_2O+H]$^+$ found 12228, calculated 12231; [**P**(d)+H_2O+H]$^+$ found 12318, calculated 12320; [**P**(d)+2H_2O+H]$^+$ found 12335, calculated 12338.

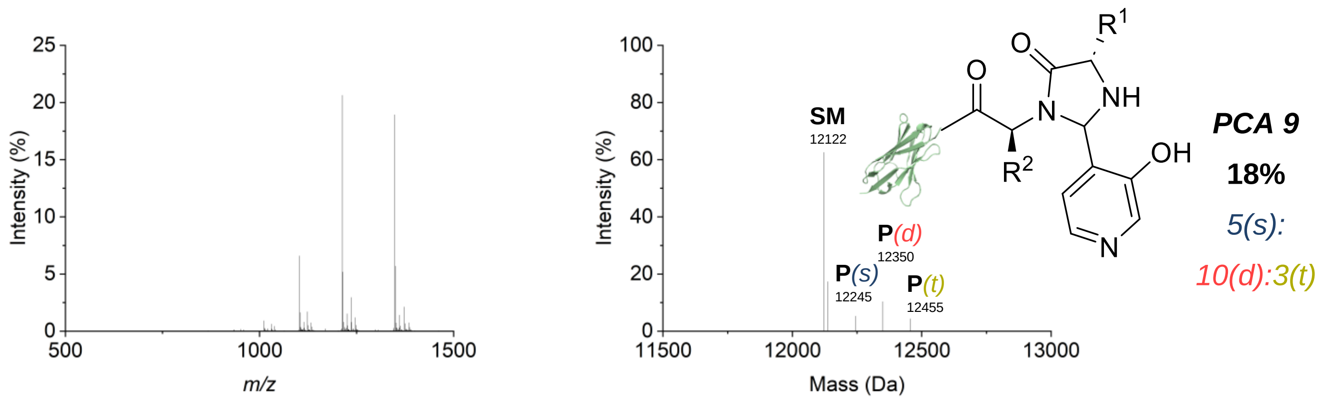

MS (ESI$^+$) [**SM**+H]$^+$ found 12122, calculated 12124; [**SM**+H_2O+H]$^+$ found 12138, calculated 12142; [**P**(s)+H_2O+H]$^+$ found 12245, calculated 12247; [**P**(d)+H_2O+H]$^+$ found 12350, calculated 12352; [**P**(t)+H_2O+H]$^+$ found 12455, calculated 12457.

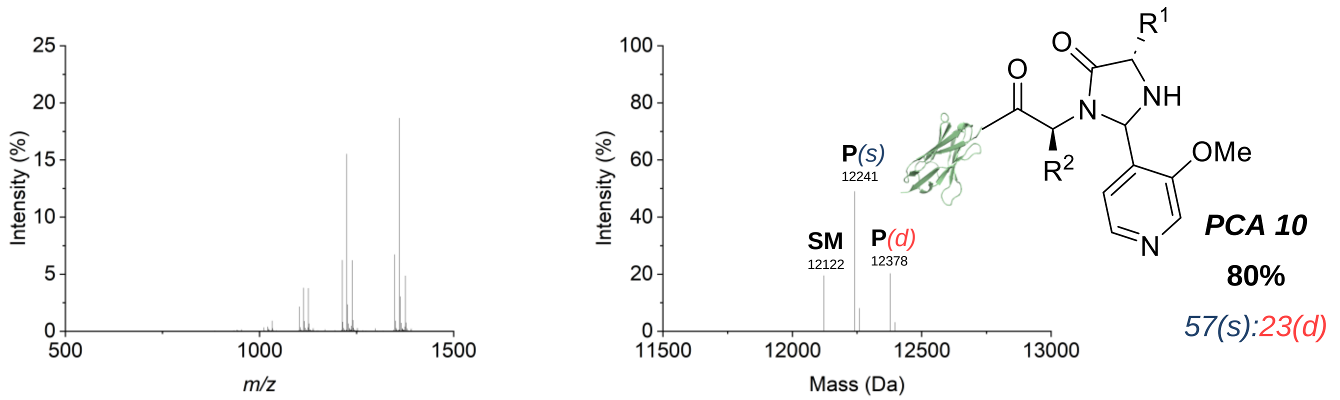

MS (ESI$^+$) [**SM**+H]$^+$ found 12122, calculated 12124; [**P**(s)+H]$^+$ found 12241, calculated 12243; [**P**(s)+H_2O+H]$^+$ found 12259, calculated 12261; [**P**(d)+H_2O+H]$^+$ found 12378, calculated 12380; [**P**(d)+2H_2O+H]$^+$ found 12396, calculated 12398.

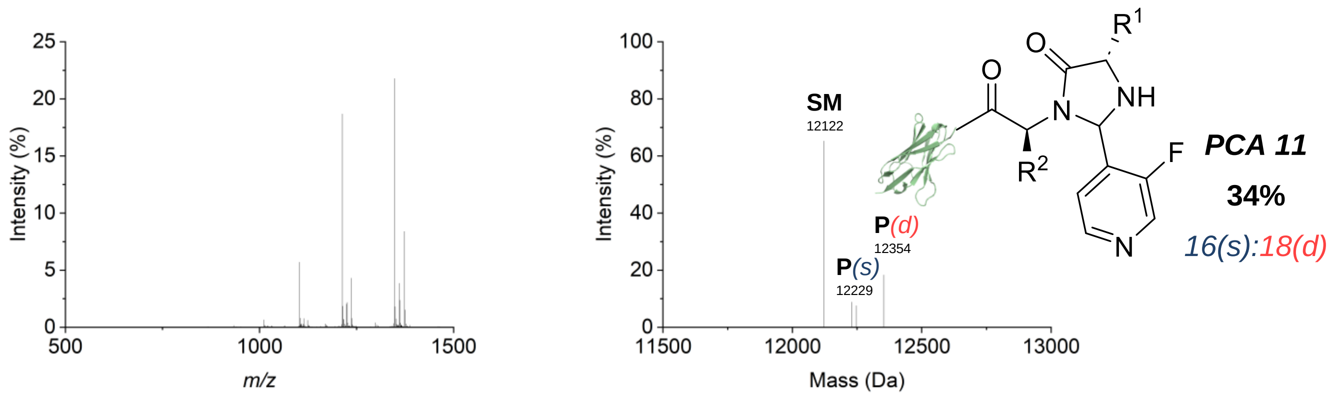

MS (ESI$^+$) [**SM**+H]$^+$ found 12122, calculated 12124; [**P**(s)+H]$^+$ found 12229, calculated 12231; [**P**(s)+H_2O+H]$^+$ found 12247, calculated 12249; [**P**(d)+H_2O+H]$^+$ found 12354, calculated 12356.

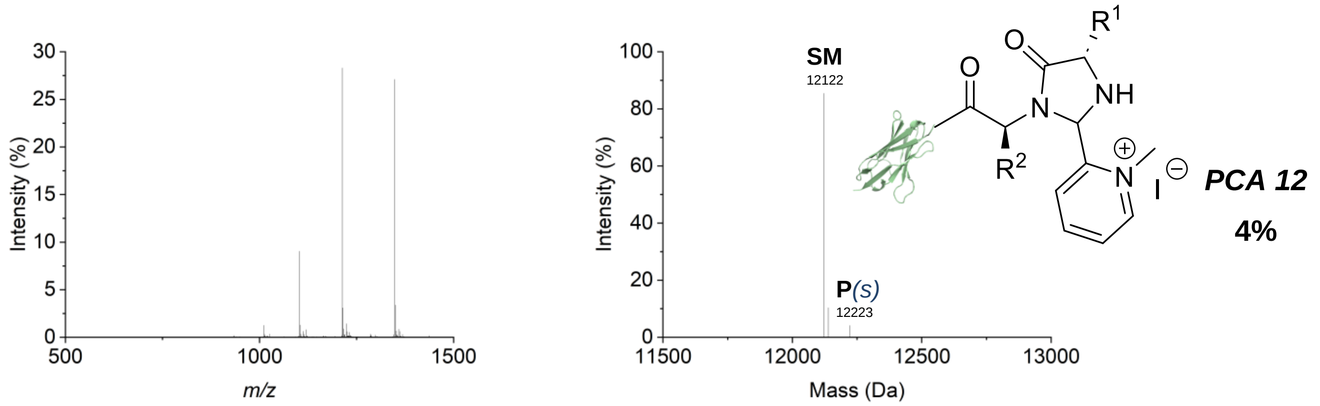

MS (ESI$^+$) [**SM**+H]$^+$ found 12122, calculated 12124; [**SM**+H_2O+H]$^+$ found 12139, calculated 12142; [**P**(s)+H]$^+$ found 12223, calculated 12228.

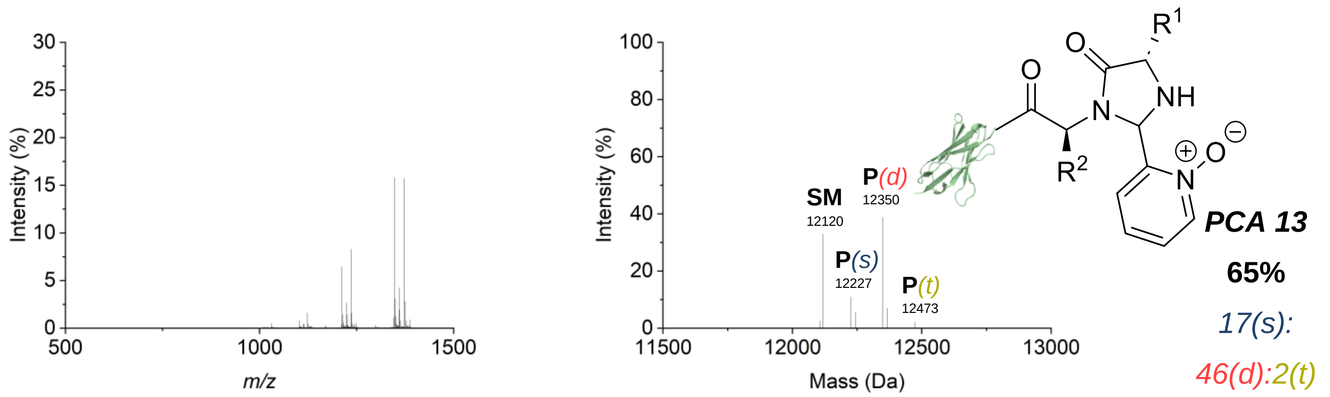

MS (ESI$^+$) [**SM**-H_2O+H]$^+$ found 12107, calculated 12106; [**SM**+H]$^+$ found 12120, calculated 12124; [**P**(s)+H]$^+$ found 12227, calculated 12229; [**P**(s)+H_2O+H]$^+$ found 12244, calculated 12247; [**P**(d)+H_2O+H]$^+$ found 12350, calculated 12352; [**P**(d)+2H_2O+H]$^+$ found 12368, calculated 12370; [**P**(t)+2H_2O+H]$^+$ found 12473, calculated 12475.

5. References

(1) Keenan, T.; Spears, R. J.; Akkad, S.; Mahon, C. S.; Hatton, N. E.; Walton, J.; Noble, A.; Yates, N. D.; Baumann, C. G.; Parkin, A.; Signoret, N.; Fascione, M. A. A Tale of Two Bioconjugations: pH Controlled Divergent Reactivity of Protein α-Oxo-Aldehydes in Competing α-Oxo-Mannich and Catalyst-Free Aldol Ligations. *ACS Chem. Biol.* **2021**, *16* (11), 2387–2400. https://doi.org/10.1021/acschembio.1c00531.

(2) Chatani, E.; Hayashi, R.; Moriyama, H.; Ueki, T. Conformational Strictness Required for Maximum Activity and Stability of Bovine Pancreatic Ribonuclease A as Revealed by Crystallographic Study of Three Phe120 Mutants at 1.4 Å Resolution. *Protein Sci.* **2002**, *11* (1), 72–81. https://doi.org/10.1110/ps.31102.

(3) Maurus, R.; Overall, C. M.; Bogumil, R.; Luo, Y.; Mauk, A. G.; Smith, M.; Brayer, G. D. A Myoglobin Variant with a Polar Substitution in a Conserved Hydrophobic Cluster in the Heme Binding Pocket. *Biochim. Biophys. Acta BBA - Protein Struct. Mol. Enzymol.* **1997**, *1341* (1), 1–13. https://doi.org/10.1016/S0167-4838(97)00064-2.

(4) Andersen, J. F.; Sanders, D. A. R.; Gasdaska, J. R.; Weichsel, A.; Powis, G.; Montfort, W. R. Human Thioredoxin Homodimers: Regulation by pH, Role of Aspartate 60, and Crystal Structure of the Aspartate 60 → Asparagine Mutant,. *Biochemistry* **1997**, *36* (46), 13979–13988. https://doi.org/10.1021/bi971004s.

(5) Wang, R.; Yan, F.; Qiu, D.; Jeong, J. S.; Jin, Q.; Kim, T. Y.; Chen, L. Traceless Cross-Linker for Photocleavable Bioconjugation. *Bioconjug. Chem.* **2012**, *23* (4), 705–713. https://doi.org/10.1021/bc200343u.

(6) Zaia, J.; Annan, R. S.; Biemann, K. The Correct Molecular Weight of Myoglobin, a Common Calibrant for Mass Spectrometry. *Rapid Commun. Mass Spectrom.* **1992**, *6* (1), 32–36. https://doi.org/10.1002/rcm.1290060108.

(7) Macdonald, J. I.; Munch, H. K.; Moore, T.; Francis, M. B. One-Step Site-Specific Modification of Native Proteins with 2-Pyridinecarboxyaldehydes. *Nat. Chem. Biol.* **2015**, *11* (5), 326–331. https://doi.org/10.1038/nchembio.1792.
(8) Dodin, G.; Bourliataud, B.; Cordier, C.; Blais, J.-C. Coupling of 2‘-Deoxyribonucleotides with 2-Pyridinealdehyde Methiodides. *J. Org. Chem.* **1996**, *61* (7), 2561–2563. https://doi.org/10.1021/jo9521312.
(9) Chemistry A European J - 2013 - Limnios - 2 2 2-Trifluoroacetophenone as an Organocatalyst for the Oxidation of Tertiary.Pdf.
(10) Chemistry A European J - 2006 - Landa - Bis Oxazoline Lewis Acid Catalyzed Aldol Reactions of Pyridine N-Oxide Aldehydes.Pdf.
(11) Roudesly, F.; Veiros, L. F.; Oble, J.; Poli, G. Pd-Catalyzed Direct C–H Alkenylation and Allylation of Azine *N* -Oxides. *Org. Lett.* **2018**, *20* (8), 2346–2350. https://doi.org/10.1021/acs.orglett.8b00689.
(12) Pérez-Faginas, P.; Aranda, M. T.; García-López, M. T.; Snoeck, R.; Andrei, G.; Balzarini, J.; González-Muñiz, R. Synthesis and SAR Studies on Azetidine-Containing Dipeptides as HCMV Inhibitors. *Bioorg. Med. Chem.* **2011**, *19* (3), 1155–1161. https://doi.org/10.1016/j.bmc.2010.12.052.
